# Local authority variation in school-recorded special educational needs and disability provision in Year 1 among children born in England, 2003–13

**DOI:** 10.1093/eurpub/ckaf116

**Published:** 2025-02-07

**Authors:** Kate M Lewis, Vincent G Nguyen, Ruth Gilbert, Bianca De Stavola, Lorraine Dearden, Ayana Cant, Johnny Downs, Laura Gimeno, William Farr, Tamsin Ford, Katie Harron, Matthew Lilliman, Stuart Logan, Jacob Matthews, Jugnoo Rahi, Jennifer Saxton, Isaac Winterburn We thank Ruth Blackburn, Milagros Ruiz, Matthew Jay, Antony Stone, Farzan Ramzan

**Affiliations:** Great Ormond Street Institute of Child Health, University College London, London, United Kingdom; Great Ormond Street Institute of Child Health, University College London, London, United Kingdom; Great Ormond Street Institute of Child Health, University College London, London, United Kingdom; Great Ormond Street Institute of Child Health, University College London, London, United Kingdom; Social Research Institute, University College London, London, United Kingdom

## Abstract

Evidence of disparities in special educational needs and disability (SEND) provision at local authority (LA) level in England is needed to guide policies for equitable provision. We described LA-level variation in recorded SEND provision using linked health-education records. We used linked hospital-primary school records (ECHILD – Education and Child Health Insights from Linked Data) to create a cohort of 3 729 265 children born in England between 2003/04-2012/13. LA of pupil’s residential address and SEND provision [SEND support or Educational Health and Care Plan (EHCP)] were defined at Year 1 (5/6 years old). We compared single-level and multilevel logistic models, adjusting for individual-level sociodemographic, health indicators, and school governance, and stratifying by gestational age. In further multilevel models, we added LA characteristics. After accounting for individual-level characteristics, there was between 2.0% (SEND support compared with no SEND provision) and 5.8% (EHCPs compared with SEND support) residual unexplained variation between LAs across gestational age groups. Adding LA-level income deprivation reduced the between-LA variance for EHCPs by 14%–24% across gestational age groups; less so for other LA characteristics. Under 6% of the differences in school-recorded SEND provision in Year 1 between 2009/10 and 2018/19 was associated with the LA context. We need to carefully disentangle structural factors at the school and individual level to understand inequities in recorded SEND provision.

## Introduction

In England, children are entitled to additional support with their learning, referred to as Special Educational Needs and Disability (SEND) provision if ‘he or she has a significantly greater difficulty in learning than the majority of others of the same age, or has a disability which prevents them from making use of facilities generally provided by mainstream schools’ [1]. Provision consists of two categories: SEND support, arranged by the school, which may include different educational materials or small group support; and Education, Health and Care Plans (EHCP), arranged by local authorities (LAs) for children whose needs cannot be met by the school and may include one-to-one support in the classroom and therapies outside school. Even with evolving legislation governing SEND provision, concerns persistently surface about under-and-over-identification of pupils requiring SEND provision across different populations in England [2–5]. In particular, SEND provision across England is described as a ‘postcode lottery’ [2–4]. In January 2022, recorded SEND support among pupils in state-funded schools in England ranged across LAs from 8.2% to 17.6% and EHCPs from 1.7% to 5.9% [6].

The extent to which these two- to three-fold differences represent inequities in service provision or appropriate differences given the underlying needs of populations living within LAs is unclear. The National Audit Office reported that ‘The Department [of Education] believes that the variation reflects local context and practice, but has not investigated the reasons’ [7]. Understanding the extent of local variation in SEND provision, and the reasons behind this, could guide prioritization of policies to improve equitable provision. Previous studies examining geographic variation and drivers of SEND provision relied solely on data from schools, without external measures of the level of need in the underlying population and were not able to account for pupil-level health characteristics [3]. In this study we used linked education and health records for a national cohort of children born in England to describe LA-level variation in recorded SEND provision at start of primary school, accounting for pupil and school-level factors, and quantify the contribution of LA-level factors to this variation. We separately analysed children grouped by gestational age at birth to investigate LA variation within groups with differing levels of need for SEND provision [8].

## Methods

### Data sources

We used the ‘Education and Child Health Insights from Linked Data’ (ECHILD) dataset [9]. ECHILD is a whole population-based cohort of children and young people in England, linking their administrative educational data (National Pupil Database [NPD]) to hospital (Hospital Episode Statistics [HES]) and social care records. We further linked opensource school characteristic data published by the Department for Education and geographical boundary data published by the Office for National Statistics (ONS) to ECHILD [10–13]. Dataset details and linkage are provided in Supplementary Table S1.

### Study population

Our cohort was defined as singleton children (excluding multiple births, e.g. twins, triplets) born in NHS-funded hospitals in England between 1 September 2003 and 31 August 2013 attending state-funded school in Year 1 (age 5 years at entry, 1 September 2009–18). Births were detected in HES APC records by a mixture of diagnostic and procedure codes and administrative variables [14]. We included children born at 34 to <42 weeks’ gestation, who had a linked NPD record, appeared in any school census in Year 1 and had residential information recorded in NPD.

### Key variables

Our outcome was recording of SEND provision for each pupil during Year 1 January School Census or pupil referral unit census (and includes both mainstream and specialist provision) coded as EHCP (EHCP or statement of SEN), SEND support (SEND support, school action or school action plus) or none. Importantly, a record of SEND provision in the educational data does not necessarily indicate that SEND provision was received, and there is no information about the precise elements of support received (hence our label of ‘recorded’ SEND provision).

LA was defined by the middle layer super output area 2001 or 2011 of each child’s residential address reported in the pupil-level school censuses (NPD) at entry into Year 1 and mapped to one of the 150 upper tier LAs in England as defined in 2021 (excluding Isle of Scilly and City of London due to small pupil number). LAs are local government organizations that are responsible for services in a defined area and its resident population, such as education, transport, and social care [15]. LAs are responsible for allocating funding for SEND support to pupils in LA-maintained schools (see governance, below), and for assessing and funding EHCPs for all pupils [16]. We stratified the analysis by gestational age, which was defined by completed weeks of gestation at birth in each child’s birth record and categorized as full term (39–<42 weeks), early term (37–<39 weeks) and late preterm (34–<37 weeks). We stratify our analysis by these groups to assess the results in groups with increasingly reduced heterogeneity in terms of the likelihood of requiring SEND provision [8]. We do not present results for preterm groups <34 weeks of gestation given small numbers in these groups at the LA level.

Several variables specific to each pupil (‘individual-level variables’) were selected *a priori* as likely associated with the underlying need for, and allocation of, SEND provision [3]: year of birth, maternal age, gender, racial-ethnic group, English as an additional language (EAL), income deprivation affecting children index (IDACI), free school meal (FSM) eligibility, chronic conditions (Supplementary Table S2) [17], hospital presentation rate, age at start of Year 1, governance of the school attended in Year 1, and early years foundation stage profile scores (EYFSP; Supplementary Table S3). Types of school governance can be split into schools that are primarily LA-maintained (community, voluntary aided, and voluntary controlled schools) and those which run independently to the LA (sponsor led academies, converter led academies, and free schools). We use the phrase ‘individual-level’ to indicate that these covariates are measured at the pupil level; however, the relationship between many of these covariates and SEND provision operates at the societal/structural level (see our framework; Supplementary Fig. S1).

Additional, contextual LA-specific variables were considered, as they may further explain variation in recorded SEND provision at the LA level: the size of the pupil population: the proportion of children attending special schools; the proportion of children attending maintained schools; the proportion of children attending academies; the proportion of children eligible for free school meals; and the modal IDACI of pupils’ home residence.

### Statistical methods

We plotted the annual proportion of children in each gestational age group against published statistics [18]. To understand variation in recorded SEND provision across LAs for each gestational age group we focussed on three comparisons: (i) SEND support vs. no SEND provision; (ii) EHCP vs. SEND support; and (iii) EHCP vs. no SEND provision. We did this because of known different pathways to allocation of provision. These comparisons were quantified by applying a sequence of models recommended by Merlo *et al.* [19].

In **step one** [19], for each of the pairwise comparisons, single-level logistic regression models were fitted including individual-level covariates. Predicted probabilities from these models were used to plot the receiving operator characteristic (ROC) curve and calculate the area under the ROC (AUC). In **step two**, these models were extended to include random intercepts that capture any additional heterogeneity across LAs, quantified using intraclass correlation coefficients (ICC). ICCs give the relative variation in (log) odds across LAs that is not explained by the covariates, ranging from 0 (indicating no clustering within LAs) to 1 (complete clustering within LAs). Caterpillar plots of the ranked predicted residuals for each LA with 95% confidence intervals (CIs) were produced to visualize their variation relative to the overall average. In **step three**, LA-level covariates were added, with changes in the estimated random intercept variance reported.

Akaike information criterion and Bayesian information criterion were calculated in each step to compare models, with smaller values indicating improved fit. Mutually adjusted (conditional) odds ratios (ORs) are reported for each model, with those for the LA-level covariates in step three capturing associations with the outcome, once individual-level characteristics and LA random intercepts are held constant. The missing covariate indicator method was used to deal with missing values (IDACI, racial-ethnic group, EAL and EYFSP) [20]. Analyses for the strata of children born early- and full-term were restricted to random subsamples (respectively, 20% and 5%) due to computational complexity. The potential impact of clustering due to a child’s school could not be examined because of the perfect collinearity between one group of schools (special schools, where an EHCP is a prerequisite for entry) and the outcome [1].

Due to high levels of missingness in gestational age prior to 2008/09, we replicated the analyses in birth years with low missingness (2008/09-2012/13). Analyses also replicated the main analyses using a minimal adjustment set (year of birth and maternal age, Supplementary Fig. S1) to assess the impact of including covariates that could plausibly be influenced by features of the child’s LA and, therefore, mediate the relationship between LA and SEND provision. Lastly, we replicated the analyses for children born early and full term with a different random sample. Analyses were conducted in the ONS Secure Research Service using Stata v18 (code available at https://github.com/UCL-CHIG/HOPE_study_LA_variation). Field names and cleaning rules can be found in Supplementary Table S3. Supplementary Box S1 outlines changes to the study since publishing our protocol [21].

## Results

There were 6 192 118 singleton live births in our dataset between 1 September 2003 and 31 August 2013, of which 4 498 991 (72.7%) had a recorded gestational age value between 34 and 41 weeks, inclusive. Missing gestational age information was associated with year of birth (26.4% in 2009/10 vs. 10.1% in 2018/19, Supplementary Fig. S2), although the proportion of live births identified as premature each year in our study is comparable to rates published elsewhere (Supplementary Fig. S3). 3 976 619 (85.9%) children had a linked NPD record and further exclusions were applied to 247 354 children (4.0% of the initial singleton births, Supplementary Fig. S4).

### Cohort characteristics

The final cohort included 3 729 265 children, of whom 138 840 (3.7%) children were born late preterm, 730 912 (19.6%) early term and 2 859 513 (76.7%) full term (Table 1). Overall, just over half of children were male (2 560 625, 51.3%), 13.5% (501 937) had a hospital-record identified chronic condition, 17.0% (632 840) were FSM eligible and three quarters of children were from a White racial-ethnic group (2 858 836, 76.7%). Almost all children (99.4%, 3 705 630) attended a mainstream school and 82.9% (3 092 202) were in a LA-maintained school (community, voluntary aided and voluntary controlled schools). In total, 14.2% (531 022) children had SEND provision recorded in Year 1, including 12.6% (468 937) with SEND support and 1.7% (62 085) with an EHCP. The distribution of characteristics differed among children with recorded SEND provision, including higher proportions of early term and late preterm births, males, chronic conditions, FSM eligibility, and community school attendance among those with SEND support and EHCPs compared with no SEND provision.

**Table 1.** Cohort characteristics, overall and by category of recorded SEND provision

		All children	Recorded SEND provision		
			None	SEND support	EHCP
		*N* (%)	*N* (%)	*N* (%)	*N* (%)
***Child-level***					
	**Total**	3 729 265 (100)	3 198 243 (100)	468 937 (100)	62 085 (100)
**Gestational age group**	**Late preterm (34–<37 weeks)**	138 840 (3.7)	111 836 (3.5)	23 058 (4.9)	3946 (6.4)
	**Early term (37–<39 weeks)**	730 912 (19.6)	610 678 (19.1)	104 551 (22.3)	15 683 (25.3)
	**Term (39–<42 weeks)**	2 859 513 (76.7)	2 475 729 (77.4)	341 328 (72.8)	42 456 (68.4)
**Year of birth (1 September to 31 August)**	**2003/04**	285 769 (7.7)	234 676 (7.3)	46 979 (10.0)	4114 (6.6)
	**2004/05**	291 924 (7.8)	241 729 (7.6)	45 893 (9.8)	4302 (6.9)
	**2005/06**	278 121 (7.5)	232 643 (7.3)	41 404 (8.8)	4074 (6.6)
	**2006/07**	275 642 (7.4)	233 264 (7.3)	38 218 (8.1)	4160 (6.7)
	**2007/08**	323 847 (8.7)	273 981 (8.6)	44 720 (9.5)	5146 (8.3)
	**2008/09**	406 697 (10.9)	351 778 (11.0)	48 092 (10.3)	6827 (11.0)
	**2009/10**	456 766 (12.2)	399 201 (12.5)	49 888 (10.6)	7677 (12.4)
	**2010/11**	470 889 (12.6)	411 465 (12.9)	51 457 (11.0)	7967 (12.8)
	**2011/12**	479 639 (12.9)	418 562 (13.1)	52 146 (11.1)	8931 (14.4)
	**2012/13**	459 971 (12.3)	400 944 (12.5)	50 140 (10.7)	8887 (14.3)
**Maternal age at birth**	**Median (IQR)**	29 (24, 33)	29 (25, 33)	27 (23, 32)	29 (24, 33)
**Gender**	**Female**	1 817 309 (48.7)	1 653 058 (51.7)	147 652 (31.5)	16 599 (26.7)
	**Male**	1 911 956 (51.3)	1 545 185 (48.3)	321 285 (68.5)	45 486 (73.3)
**Chronic condition**	**No**	3 227 328 (86.5)	2 824 412 (88.3)	373 391 (79.6)	29 525 (47.6)
	**Yes**	501 937 (13.5)	373 831 (11.7)	95 546 (20.4)	32 560 (52.4)
**IDACI**	**1 Most deprived 20%**	994 012 (26.7)	801 008 (25.0)	173 459 (37.0)	19 545 (31.5)
	**2**	792 716 (21.3)	664 515 (20.8)	113 580 (24.2)	14 621 (23.5)
	**3**	691 764 (18.5)	602 018 (18.8)	78 642 (16.8)	11 104 (17.9)
	**4**	650 624 (17.4)	582 158 (18.2)	59 331 (12.7)	9135 (14.7)
	**5 Least deprived 20%**	599 703 (16.1)	548 193 (17.1)	43 850 (9.4)	7660 (12.3)
	**Missing**	446 (<0.01)	351 (<0.01)	75 (<0.01)	20 (<0.01)
**FSM eligible**	**No**	3 096 425 (83.0)	2 725 795 (85.2)	325 454 (69.4)	45 176 (72.8)
	**Yes**	632 840 (17.0)	472 448 (14.8)	143 483 (30.6)	16 909 (27.2)
**Racial-ethnic group**	**Asian**	379 069 (10.2)	321 742 (10.1)	49 446 (10.5)	7881 (12.7)
	**Black**	185 327 (5.0)	152 161 (4.8)	28 255 (6.0)	4911 (7.9)
	**Chinese**	15 988 (0.4)	14 250 (0.4)	1445 (0.3)	293 (0.5)
	**Mixed**	224 302 (6.0)	192 900 (6.0)	27 461 (5.9)	3941 (6.3)
	**Other**	54 070 (1.4)	45 471 (1.4)	7419 (1.6)	1180 (1.9)
	**White**	2 858 836 (76.7)	2 461 498 (77.0)	353 711 (75.4)	43 627 (70.3)
	**Missing**	11 673 (0.3)	10 221 (0.3)	1200 (0.3)	252 (0.4)
**EAL**	**No**	3 168 500 (85.0)	2 722 768 (85.1)	394 777 (84.2)	50 955 (82.1)
	**Yes**	551 573 (14.8)	467 880 (14.6)	72 747 (15.5)	10 946 (17.6)
	**Unclear or missing**	9192 (0.2)	7595 (0.2)	1413 (0.3)	184 (0.3)
**Rate of hospitalization**	**per 1000 person-days (95% CI)**	2 (1, 5)	2 (1, 5)	3 (1, 6)	5 (2, 5)
**Age at Year 1 start**	**Median (IQR)**	5.49 (5.24, 5.75)	5.51 (5.25, 5.76)	5.40 (5.18, 5.67)	5.49 (5.24, 5.75)
**School governance**	**Community**	2176 722 (58.4)	1 837 953 (57.5)	295 113 (62.9)	43 656 (70.3)
	**Sponsor led academy**	174 393 (4.7)	147 727 (4.6)	24 280 (5.2)	2386 (3.8)
	**Converter led academy**	440 000 (11.8)	385 429 (12.1)	47 131 (10.1)	7440 (12.0)
	**Free school**	22 670 (0.6)	20 043 (0.6)	2239 (0.5)	388 (0.6)
	**Voluntary aided**	603 422 (16.2)	531 108 (16.6)	66 953 (14.3)	5361 (8.6)
	**Voluntary controlled**	312 058 (8.4)	275 983 (8.6)	33 221 (7.1)	2854 (4.6)
**Incomplete/missing EYFSP score**	**No**	35 371 (0.9)	26 484 (0.8)	5532 (1.2)	3355 (5.4)
	**Yes**	3 693 894 (99.1)	3 171 759 (99.2)	463 405 (98.8)	58 730 (94.6)
**Standardized EYFSP score**	**Median (IQR)**	0.03 (−0.39, 0.60)	0.09 (−0.11, 0.70)	−0.99 (−1.77, −0.27)	−2.28 (−2.31, 0.61)
***LA-level***					
**Pupil population^b^**	**Median (IQR)**	3725 (2635, 6915)	3750 (2640, 6920)	3590 (2600, 6835)	3520 (2585, 6920)
**Special school attendance (%)**	**Median (IQR)**	0.66 (0.46, 0.87)	0.66 (0.46, 0.87)	0.66 (0.45, 0.88)	0.71 (0.52, 0.87)
**Maintained school attendance (%)**	**Median (IQR)**	88.1 (73.2, 97.5)	87.9 (72.9, 97.2)	90.3 (75.7, 98.8)	86.9 (72.2, 97.5)
**Academy attendance (%)**	**Median (IQR)**	10.9 (2.3, 26.0)	11.3 (2.4, 26.3)	8.6 (1.0, 23.8)	11.9 (3.4, 26.2)
**FSM eligible (%)**	**Median (IQR)**	15.6 (12.1, 21.4)	15.5 (12.1, 21.1)	16.8 (12.8, 23.1)	15.6 (12.1, 21.2)
**IDACI mode**	**1 most deprived 20%**	1 430 511 (38.4)	1 203 282 (37.6)	202 841 (43.3)	24 388 (39.3)
	**2**	519 814 (13.9)	446 210 (14.0)	64 488 (13.8)	9116 (14.7)
	**3**	549 345 (14.7)	475 709 (14.9)	66 010 (14.1)	7626 (12.3)
	**4**	463 126 (12.4)	402 937 (12.6)	51 561 (11.0)	8628 (13.9)
	**5 least deprived 20%**	766 469 (20.6)	670 105 (21.0)	84 037 (17.9)	12327 (19.9)

EAL, English as an additional language; EHCP, education, health and care place; EYFSP, early years foundation stage profile; FSM, free school meals; IDACI, income deprivation affecting children index; IQR, interquartile range; LA, local authority; SEND, special educational needs and disability.

a Split into nine subgroups for main analyses (see Supplementary Table S3).

b Rounded to the nearest 5 as per statistical disclosure rules.

### Step one—single-level logistic regression

The late preterm group had the best model fit in terms of AUCs, 0.84 (95% CI 0.84, 0.85) for SEND support vs. no SEND provision, 0.81 (95% CI 0.80, 0.81) for EHCPs vs. SEND support, and 0.87 (95% CI 0.86, 0.87) for EHCPs vs. no SEND provision, indicating medium-high prediction of outcome classifications based on the individual-level covariates (Table 2, Supplementary Table S4). AUCs were slightly lower for early term and full-term. Conditional ORs for individual level covariates were similar across gestational age groups, Supplementary Tables S4–S6, with particularly elevated ORs for children with a hospital-record defined chronic mental health/developmental or neurological condition, compared to children without the relevant condition (OR 35.0, 95% CI 30.1,40.7, and OR 9.37, 95% CI 8.56,10.26, respectively, for EHCP vs. no SEND provision in children born late preterm). Higher socioeconomic deprivation (measured by IDACI and FSM eligibility) was associated with higher odds of SEND support vs. no provision and EHCPs vs. no provision, but lower odds of EHCP vs. SEND support. Children from White racial-ethnic groups had the lowest odds of EHCP vs. SEND support and EHCP vs. no provision. Attending a community school was associated with higher odds of SEND provision across all comparison groups. A one standard deviation increase in EYFSP score was associated with 73%–75% lower odds of SEND support vs. no provision (OR 0.25, 95% CI 0.24, 0.25, late preterm group).

**Table 2.** Between-LA effects, step one and two logistic regression models, by outcome: stratified by gestational age group

	SEND support vs. no provision		EHCP vs. SEND support		EHCP vs. no provision	
	Step one (single-level)	Step two (multilevel)	Step one (single-level)	Step two (multilevel)	Step one (single-level)	Step two (multilevel)
**Late preterm (34–<37 weeks)**						
LA variance^a^ (95% CI)		0.08 (0.06, 0.10)		0.20 (0.15, 0.28)		0.14 (0.10, 0.20)
AUC (95% CI)	0.84 (0.84, 0.85)	0.85 (0.85, 0.85)	0.81 (0.80, 0.81)	0.83 (0.82, 0.83)	0.87 (0.86, 0.87)	0.88 (0.87, 0.89)
AUC change^b^		0.004		0.02		0.01
ICC % (95% CI)		2.26 (1.73, 2.95)		5.84 (4.26, 7.97)		4.07 (2.86, 5.77)
**Early term (37–<39 weeks)**						
LA variance^a^ (95% CI)		0.07 (0.06, 0.10)		0.17 (0.11, 0.25)		0.14 (0.09, 0.20)
AUC (95% CI)	0.85 (0.85, 0.85)	0.85 (0.85, 0.86)	0.78 (0.77, 0.79)	0.80 (0.79, 0.81)	0.85 (0.84, 0.85)	0.86 (0.85, 0.87)
AUC change^b^		0.004		0.02		0.02
ICC % (95% CI)		2.17 (1.65, 2.85)		4.86 (3.37, 6.97)		3.98 (2.69, 5.85)
**Full term (39–<42 weeks)**						
LA variance^a^ (95% CI)		0.07 (0.05, 0.09)		0.15 (0.09, 0.23)		0.13 (0.08, 0.21)
AUC (95% CI)	0.86 (0.85, 0.86)	0.86 (0.86, 0.86)	0.75 (0.74, 0.76)	0.78 (0.77, 0.79)	0.82 (0.81, 0.83)	0.84 (0.83, 0.85)
AUC change^b^		0.004		0.02		0.02
ICC % (95% CI)		2.03 (1.53, 2.70)		4.22 (2.76, 6.41)		3.79 (2.42, 5.88)

AUC, area under the receiving operator characteristic curve; CI, confidence interval; EHCP, education, health and care place; LA, local authority; SEND, special educational needs and disability.

a Random intercept variance from multilevel logistic regression models.

b Change in relation to the step one, with positive values indicating an increase in the discriminatory accuracy of the model.

### Step two—multilevel logistic regression

**Figure 1.**
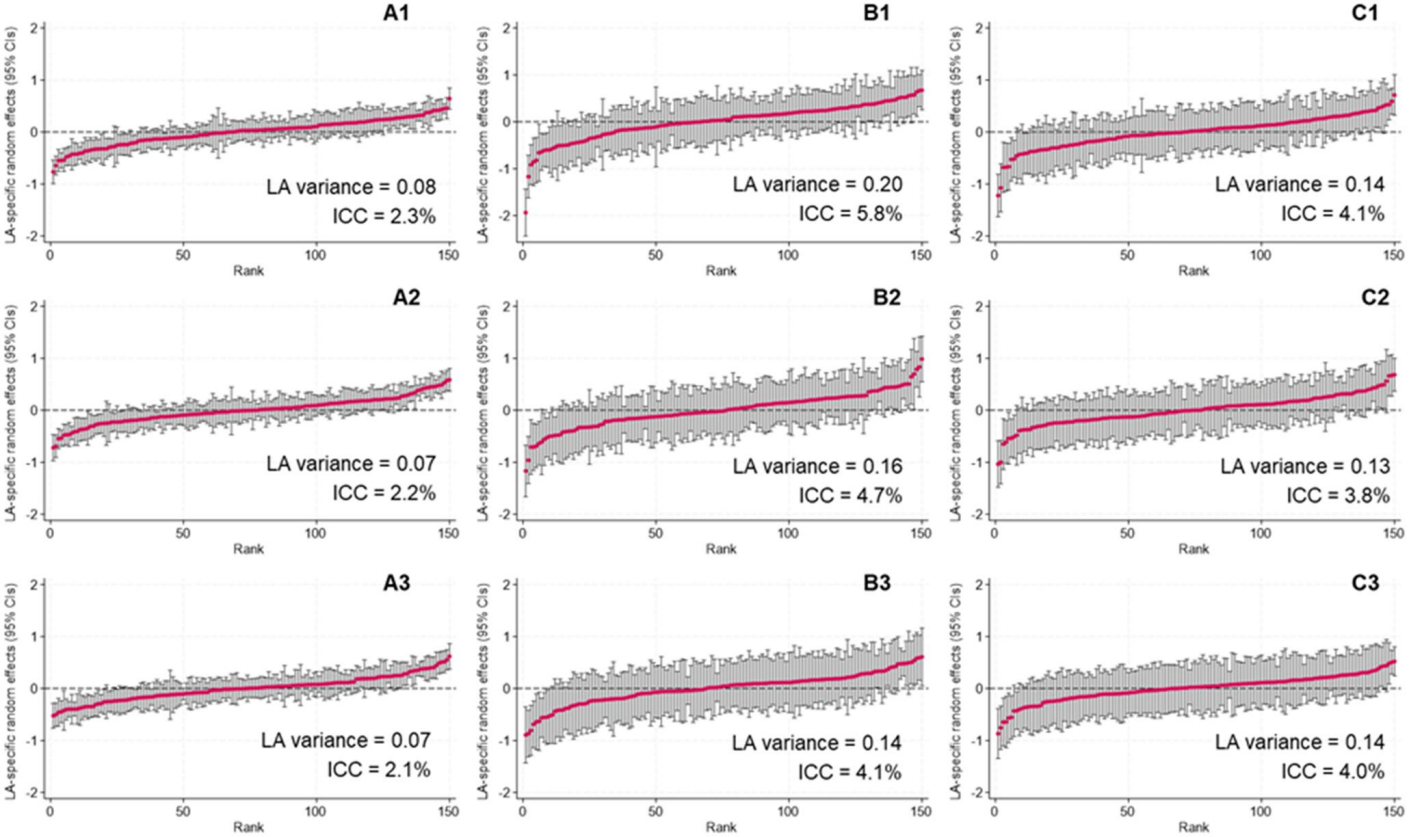
LA-specific random effects from step 2 (multilevel) logistic regression models of (A) SEND support vs. no SEND provision, (B) EHCP vs. SEND support, and (C) EHCP vs. no SEND provision with 95% CIs vs. LA rank: stratified by gestational age groups (1) late preterm, (2) early term, and (3) full term. EHCP = education health and care plan; ICC, intraclass correlation coefficient; SEND, special educational needs and disability.

Across LAs, the median proportion of SEND support was 12.5% (interquartile range 11.0%–14.3%) and 1.7% for EHCPs (interquartile range 1.5%–2.0%). The inclusion of a random LA effect had minimal effect on the AUCs or the conditional ORs for individual level covariates, with ROCs for step one and two models overlapping for all analyses (Table 2; Supplementary Tables S4–S6; Supplementary Fig. S5). The ICCs in the late preterm group models were 2.26 (95% CI 1.73, 2.95) for SEND support vs. none, 5.84 (95% CI 4.26, 7.97) for EHCPs vs. SEND support and 4.07 (95% CI 2.86, 5.77) for EHCPs vs. none. The same pattern, at lower values, was estimated for the early term and full-term groups. Most LAs have CIs for the random effects that cross zero, signifying no evidence of statistical difference across LAs (Fig. 1).

### Step three

**Figure 2.**
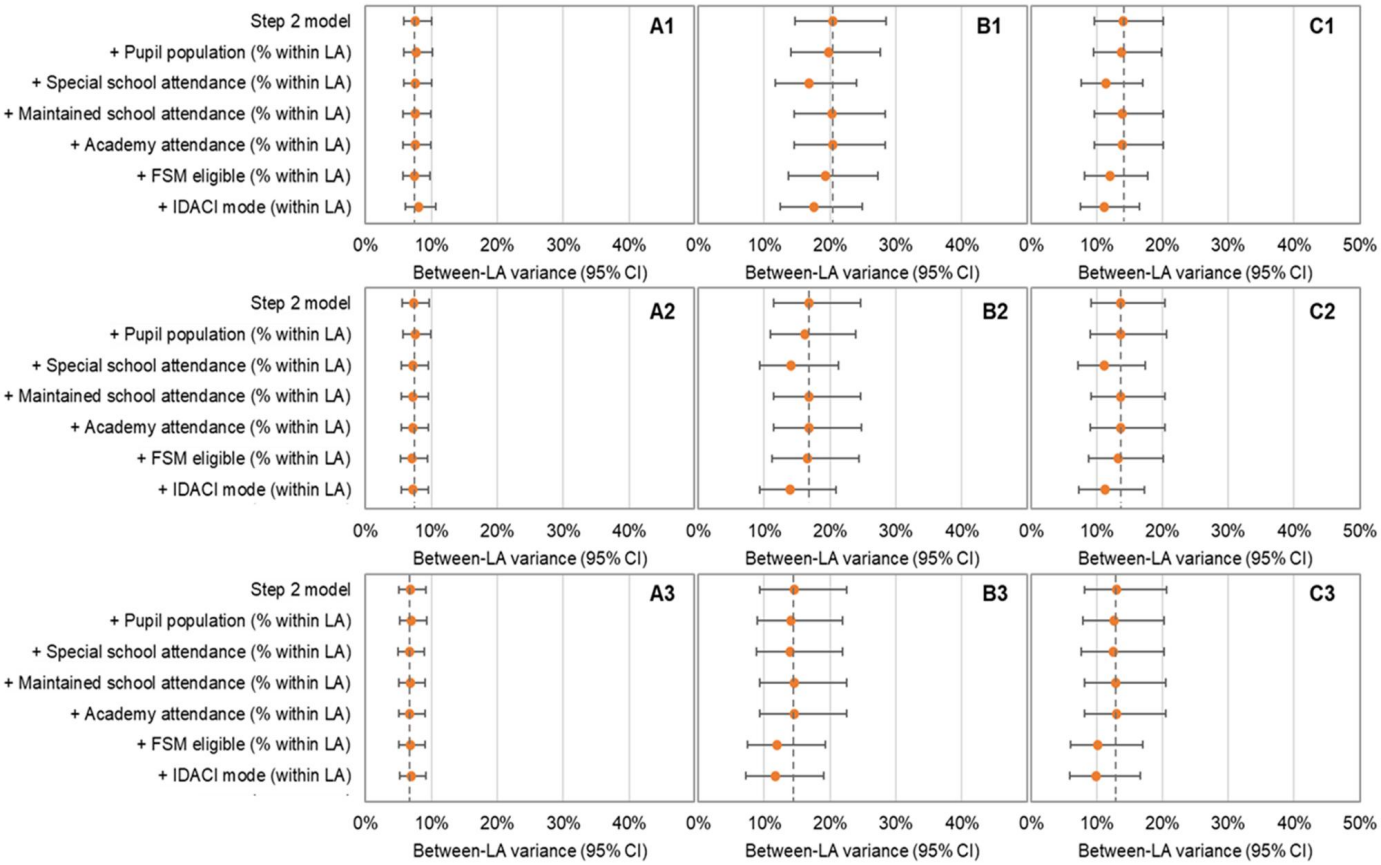
Between- LA variance (95% CIs) from multilevel logistic regression models of (A) SEND support vs. no SEND provision, (B) EHCP vs. SEND support, and (C) EHCP vs. no SEND provision with 95% CIs, including various LA-specific characteristics: stratified by gestational age groups (1) late preterm, (2) early term, and (3) full term. Dashed line indicates variance from step 2 model. CI, confidence interval; FSM, free school meals; IDACI, income deprivation affecting children index; SEND, special educational needs and disability.

Adding LA-level covariates reduced between-LA variance by <3% for all comparisons of SEND support vs. no SEND provision (Fig. 2; Supplementary Tables S4–S6). For comparisons of EHCP vs. SEND Support and EHCPs vs. no SEND provision, inclusion of modal IDACI led to a 14.1%–23.8% reduction in between-LA variance explained across gestational age groups. Children in LAs with the most vs. the least deprived modal IDACI had lower odds of EHCPs in both pairwise comparisons, conditional ORs 0.81 (95% CI 0.65, 1.00) and 0.78 (95% CI 0.64, 0.94), respectively, for the late preterm group. Inclusion of the percentage of pupils attending special school led to 16.3%–18.6% reduction in between-LA variance for EHCP comparisons in the late preterm and early term groups.

### Sensitivity analyses

Replication of the analyses on births from 2008/09–2012/13 did not change the main results (sensitivity 1, Supplementary Table S7–S9). Using a minimal adjustment set led to a drop in the AUC (0.54–0.59 across all step-one analyses; sensitivity 2, Supplementary Table S7–S9), indicating very low discriminatory accuracy based on individual-level covariates. Adding random LA intercepts increased AUC values by at least 0.05 (to between 0.59 and 0.64), but at the highest, the accompanying ICC was 2.98 (95% CI 1.99, 4.43, for ECHP vs. SEND support in the early preterm group). Selecting a different random sample for the early term and full-term groups also did not change the main results (sensitivity 3, Supplementary Table S7–S9).

## Discussion

Using linked health-education records to account for characteristics of the underlying population, we found minimal difference in the likelihood of recorded SEND provision in Year 1 between pupils living in different LAs. We grouped children by gestational age, finding the highest proportion of LA-level variation in recorded EHCPs among children born late preterm (the most homogeneous group of children in terms of underlying need); however, at most, we estimate that 5.8% of variation in the odds of an EHCP compared with SEND support was associated with LA of residence. LA-level income deprivation and special school attendance (for late preterm and early term groups only) attenuated the, markedly small, proportion of LA variation in EHCPs compared with SEND support or no SEND provision. Overall, these results suggest that the variation in recorded SEND provision in Year 1 between LAs operated, for the most part, at the individual and school level.

This study benefits from using the first national dataset of linked state-funded hospital and school records in England [9]. Through the inclusion of health records, we were able to use gestational age as a proxy for the need for SEND provision, improving the interpretability of our results, as well as other health-related indicators. Despite high population coverage, our cohort was limited to those born in an NHS hospital in England with linked state-school records. Nonetheless, similarity between our results and research from the Education Policy Institute (using all pupils) gives us confidence in the representativeness of our main findings. We had no missing records of SEND provision, showing the value of administratively captured data. However, a record of SEND provision in educational data is an acknowledgement of assignment of SEND provision and not evidence of receipt or indeed an accurate indicator of need in the population. Research analysing the content of EHCPs and local offers—mandated online information about local SEND provision—point to the low and varying quality of information to inform pathways once EHCPs are in place [20, 22]. Our analysis captures prevalent recorded SEND provision at one-time point only. We used Year 1 as it is the first full year that all children must be in education; however, the pathways to EHCPs can begin from birth [1]. While this study does not capture the timeliness nor quality of SEND provision, it nonetheless enables us to study differences across administrative areas at a key point in a child’s educational journey.

Within a cohort of children who started Reception in 2010/11, the Education Policy Institute similarly estimated that 4% of variation for EHCPs (compared with no EHCP) and 2% for SEND support (compared with no SEND support) during Years 1–4 were explained at the LA level [3]. We extend this work by incorporating health information and splitting the outcome into three comparison groups, whilst focusing on a specific school Year. By presenting results separately by gestational age group, we find a slightly higher level of variation explained at the LA level (6%) for children born late preterm with a recorded EHCP compared with SEND support (a comparison not previously assessed). This was likely driven, in part, by Newham (an LA in East London), which is an outlier in the comparison of EHCPs and SEND support (see the lowest ranked LA in Fig. 2, panel 1B). Newham had a different model of SEND provision over the period of our study, funding SEND provision through high needs funding rather than statutorily assessed EHCPs [23]. However, following ‘significant areas of weakness in the area’s practice’ identified during a joint inspection from Ofsted and the Care Quality Commission, Newham received a Written Statement of Action in December 2021 [24]. The proportion of children with EHCPs has since tripled, from 0.8% in 2019/20–2.5% in 2022/23, but remains far lower than the national average (4.0% in 2022) [23].

Echoing previous research [3], we found that pupils were less likely to have an EHCP recorded in LAs with a higher concentration of children living in income deprivation than those with fewer deprived children, pointing to the likely impact of constrained resource allocation. As recommended elsewhere [25], government oversight and targeted investment is needed to ensure that provision is not rationed across the country. The association with special school attendance is circular to some extent, as an EHCP is required to attend a special school. However, the importance of special school availability within the wider debate around SEND provision is illustrated by Newham, where policy decisions around inclusive education have led to limited special school places and more children requiring out placement outside Newham [26]. Owing to this overlap between special school and SEND provision, we did not include schools as a cluster in our models. However, other studies from England have recorded ICC values of 60%–69% for variation in the assignment of SEND provision at the school-level [25], highlighting the far greater contribution of school attended within the LA, compared to the LA itself. In contrast, a similar study from Wales estimated that only 5% of the variation was explained by the school level [27]. This cross-country variation may be explained by differences in school governance; there are no state-funded schools that operate independently of the LA in Wales [27]. We found that the odds of both recorded EHCPs and SEND support were consistently higher among children attending community (the only school type fully maintained by the LA). From our study, we cannot infer whether attendance at non-community schools leads to a lower allocation of SEND provision or that these schools are less likely to enrol pupils with, or likely to need, SEND provision. However, research on academies in a secondary school population using a quasi-experimental approach indicates that a combination of both reasons is likely [28]. Future research could leverage differences in SEND assignment across school types to quantify the causal effects of SEND provision on future educational outcomes.

Overall, for the period and school Year studied, we found, on average, minimal difference in the likelihood of recorded SEND provision between pupils living in different LAs. We demonstrated that presenting caterpillar plots, alongside a single measure of variation (such as the ICC), can help to elucidate the level of uncertainty when ranking LAs [19], rather than just presenting point estimates. However, a lack of variation across LAs does not imply that SEND provision is equitable *within* LAs. Indeed, our findings highlight differences in the likelihood of recorded SEND provision by sociodemographic and school attended, even among children with similar health profiles. Further research is needed to untangle these patterns to guide policy. This needs concise and targeted research questions that exploit the strengths of longitudinal administrative records, which, on the downside, do not capture the receipt or quality of SEND provision. Better measures of need among all children, along with detailed recording of the components of received SEND provision, would enable more accurate identification of inequities in provision using administrative data.

## Supplementary Material

ckaf116_Supplementary_Data

## Data Availability

The ECHILD database is made available for free for approved research based in the UK, via the ONS Secure Research Service. Enquiries to access the ECHILD database can be made by emailing ich.echild@ucl.ac.uk. Researchers will need to be approved and submit a successful application to the ECHILD Data Access Committee and ONS Research Accreditation Panel to access the data, with strict statistical disclosure controls of all outputs of analyses.
