# Supplementary material for: Local authority variation in school-recorded special educational needs and disability provision in Year 1 among children born in England, 2003–13
Source: Eur J Public Health. 2025 Feb 7;36(2):ckaf116. doi: 10.1093/eurpub/ckaf116 (PMC13064508; doi:10.1093/eurpub/ckaf116)
Supplement: ckaf116_Supplementary_Data [file ckaf116_supplementary_data.zip › Supplementary_file_LA variationEURPUB.docx]

**Table S1.** Datasets used in this study

| Dataset | Common acronym | Data provider | Description | In ECHILD?* |
| --- | --- | --- | --- | --- |
| HES admitted patient care | HES APC | NHS England | Episode level data on inpatient and day case discharges from English NHS hospitals and English NHS commissioned activity in the independent sector. Captures approximately 97% of births (the child's “birth record”),^10^ with the majority linkable to their mother’s delivery record using a mixture of deterministic and probabilistic methods applied to non-disclosive variables available in both sets of records.^11^ | Yes (linkage between birth and delivery records occurred prior to ECHILD linkage) |
| HES accident and emergency | HES A&E | NHS England | Attendances at A&E in English NHS hospitals and English NHS commissioned activity in the independent sector | Yes |
| NPD school censuses pupil-level |  | Department for Education | Pupil-level information for pupils in state-maintained educational settings in England, including the termly “school census”, and pupil referral unit census | Yes |
| NPD early years foundation stage profile | EYFSP | Department for Education | Pupil-level results from the statutory assessment of children in reception year in state schools in England | Yes |
| Get information about schools database | GIAS | Department for Education | Opensource information about schools and colleges in England, including names, type, establishment group and governance (formerly Edubase) | No – linked via school URN |
| School and College performance measures |  | Department for Education | Opensource data on results of exams and other performance measures by school and college in England | No – linked via school URN |
| Schools, pupils and their characteristics |  | Department for Education | Opensource school-level data, including pupil numbers and their characteristics | No – linked via school URN |
| National Statistics Postcode Lookup | NSPL | ONS | Opensource geographical information mapping Census Output Areas to a range of higher statistical geographies | No – linked via child’s residential MSOA11 |
| ONS Postcode Directory | ONSPD | ONS | Opensource geographical information mapping all current and terminated UK postcodes to a wide range of administrative, health and other geographic areas | No – linked via child’s residential MSOA11 |

HES = hospital episode statistics; MSOA11 = middle super output area 2011; NHS = National Health Service; ONS = Office for National Statistics; URN = unique reference number; *There is no common pseudonymised identifier across individuals in HES and NPD, therefore records are linked by NHS England using deterministic linking algorithms based on name, date of birth, sex and postcode

**Table S2.** International Classification of Diseases version 10 (ICD-10) codes used to identify children with chronic conditions, as defined using the Hardelid list (Hardelid, Dattani & Gilbert, 2024)

| Category | Subcategory | ICD-10 codes (in any diagnostic position) |
| --- | --- | --- |
| Cancer/ blood disorders | Anaemia and other blood disorders | D50*, D56.0, D56.1, D56.2, D56.4, D56.8, D56.9, D57.0, D57.1, D57.2, D57.8, D58, D61.0, D61.9, D64*, D66, D67, D68.0, D68.1, D68.2, D68.4, D68.5, D68.6, D68.8, D68.9, D69, D70, D71, D72, D73, D74, D75, D76, M36.2, M36.3, M36.4, M90.4, N08.2, Z86.2 |
|  | Immunological disorders | D80, D81, D82, D83, D84, G53.2, Q98.0 |
|  | Neoplasms | C00, C01, C02, C03, C04, C05, C06, C07, C08, C09, C10, C11, C12, C13, C14, C15, C16, C17, C18, C19, C20, C21, C22, C23, C24, C25, C26, C30, C31, C32, C33, C34, C37, C38, C39, C40, C41, C43, C44, C45, C46, C47, C48, C49, C50, C51, C52, C53, C54, C55, C56, C57, C58, C60, C61, C62, C63, C64, C65, C66, C67, C68, C69, C70, C71, C72, C73, C74, C75, C76, C77, C78, C79, C80, C81, C82, C83, C84, C85, C86, C88, C90, C91, C92, C93, C94, C95, C96, C97, D00, D01, D02, D05, D06, D07, D09, D12, D13, D14.1, D14.2, D14.3, D14.4, D15, D20, D32, D33, D34, D35, D37, D38, D39, D40, D41, D42, D43, D44, D45, D46, D47, D48, D63.0, E34.0, E88.3, G13.0, G13.1, G53.3, G55.0, G63.1, G73.1, G73.2, G94.1, M36.0, M36.1, M49.5, M82.0, M90.6, M90.7, N08.1, N16.1, Y43.1, Y43.2, Y43.3, Y84.2, Z08, Z51.0, Z51.1, Z51.2, Z54.1, Z54.2, Z85, Z86.0, Z92.3 |
| Cardiovascular | Congenital heart disease | Q20, Q21, Q22, Q23, Q24, Q25, Q26, Q89.3 |
|  | Other | I00*, I01*, I02*, I05*, I06*, I07*, I08*, I09*, I10*, I11*, I12*, I13*, I15*, I20*, I21*, I22*, I23*, I24*, I25*, I26*, I27*, I28*, I31*, I32*, I33*, I34*, I35*, I36*, I37*, I38*, I39*, I41*, I42.0*, I42.1*, I42.2*, I42.3*, I42.4*, I42.5*, I42.7*, I42.8*, I42.9*, I43.0*, I43.1*, I43.2*, I43.8*, I44.1*, I44.2*, I44.3*, I44.4*, I44.5*, I44.6*, I44.7*, I45.1*, I45.2*, I45.3*, I45.4*, I45.5*, I45.6*, I45.8*, I45.9*, I46*, I47*, I48*, I49*, I50*, I51*, I52.8*, I70*, I71*, I72.1*, I72.2*, I72.3*, I72.4*, I72.8*, I72.9*, I73*, I74*, I77*, I79.0*, I79.1*, I79.8*, I81*, I82*, I98*, I99*, M03.6, N08.8, Q27, Q28, S26*, T82.0, T82.1, T82.2, T82.3, T82.5, T82.6, T82.7, T82.8, T82.9, T86.2, Y60.5, Y61.5, Y62.5, Y84.0, Z45.0, Z50.0, Z94.1, Z95 |
| Chronic Infections | HIV | B20, B21, B22, B23, B24, F02.4, R75, Z21 |
|  | Other | A50, A81, B18, B37.1, B37.5, B37.6, B37.7, B38.1, B39.1, B40.1, B44.0, B44.7, B45, B46, B48.7, B50.0, B50.8*, B51.0, B51.8*, B52.0, B52.8*, B55, B57.2, B57.3, B57.4, B57.5, B58.0, B59, B67, B69, B73, B74, B78.7, B90, B91, B92, B94, F02.1, K23.1, K93.1, M00, N33.0, P35.0, P35.1, P35.2, P35.8, P35.9, P37.1 |
|  | Tuberculosis | A15, A16, A17, A18, A19, E35.0, K23.0, K67.3, K93.0, M01.1, M49.0, P37.0 |
| Mental health/ developmental | Developmental disorders | F70, F71, F72, F73, F78, F79, F80.0, F80.1, F80.2, F80.8, F80.9, F81, F82, F83, F84, F88, F89, F90, F91, F92, F93, F94, F95, F98 |
|  | Other mental health problems | F00, F01, F02.8, F03, F04, F05, F06, F07, F09, F20, F21, F22, F23, F24, F25, F28, F29, F50, F53, F54, F59*, F60, F61, F62, F63, F64, F65, F66, F68, F69, F99*, Z09.3*, Z50.4*, Z86.5, Z91.4* |
|  | Self-harm | X60, X61, X62, X63, X64, X65, X66, X67, X68, X69, X70, X71, X72, X73, X74, X75, X76, X77, X78, X80, X81, X82, X83, X84, ~~Y10**, Y11**, Y12**, Y13**, Y14**, Y15**, Y16**, Y17**, Y18**, Y19**, Y20**, Y21**, Y22**, Y23**, Y24**, Y25**, Y26**, Y27**, Y28**, Y30**, Y31**, Y32**, Y33**, Y34**, Y87.0**, Y87.2~~**, Z91.5 |
|  | Substance abuse | E24.4, F10, F11, F12, F13, F14, F15, F16, F17, F18, F19, F55*, G24.0*, G31.2, G40.5, G62.1, G72.0, G72.1, I42.6, K29.2, K70, K85.2, K85.3, K86.0, O35.4, R78.1*, R78.2*, R78.3*, R78.4*, R78.5*, Y47, Y49, Z50.2, Z50.3, Z71.4, Z71.5, Z72.2*, Z86.4 |
| Metabolic/ endocrine/ digestive/ renal/ genitourinary | Congenital anomalies of the digestive/ renal/ GU system | Q38.0, Q38.3, Q38.4, Q38.6, Q38.7, Q38.8, Q39, Q40.2, Q40.3, Q40.8, Q40.9, Q41, Q42, Q43.1, Q43.3, Q43.4, Q43.5, Q43.6, Q43.7, Q43.9, Q44, Q45, Q50.0, Q51, Q52.0, Q52.1, Q52.2, Q52.4, Q54.0, Q54.1, Q54.2, Q54.3, Q54.8, Q54.9, Q55.0, Q55.5, Q56, Q60.1, Q60.2, Q60.4, Q60.5, Q60.6, Q61, Q62.0, Q62.1, Q62.2, Q62.3, Q62.4, Q62.5, Q62.6, Q62.8, Q63.0, Q63.1, Q63.2, Q63.8, Q63.9, Q64, Q79.2, Q79.3, Q79.4, Q79.5, Q87.8, Q89.1, Q89.2 |
|  | Diabetes | E10, E11, E12, E13, E14, G59.0, G63.2, I79.2, M14.2, N08.3, O24, Y42.3 |
|  | Digestive | K20, K21.0, K22, K23.8, K25, K26, K27, K28, K29.0, K29.1, K29.3, K29.4, K29.5, K29.6, K29.7, K29.8, K29.9, K31, K50, K51, K52, K55, K57, K59.2, K63.0, K63.1, K63.2, K63.3, K66, K72, K73, K74, K75, K76, K80, K81, K82, K83, K85.0, K85.1, K85.8, K85.9, K86.1, K86.2, K86.3, K86.8, K86.9, K87.0, K90, M07.4, M07.5, M09.1, M09.2, T86.4, Z43.2, Z43.3, Z43.4, Z46.5, Z90.3, Z90.4, Z93.2, Z93.3, Z93.4, Z93.5 |
|  | Injuries | S36*, S37*, S38*, S39.6*, S39.7*, T06.5*, T28*, T91.5* |
|  | Metabolic | D55, E70, E71, E72, E74, E75, E76, E77, E78, E79.1, E79.8, E79.9, E80.0, E80.1, E80.2, E80.3, E80.5, E80.7, E83, E85, E88.0, E88.1, E88.2*, E88.8, E88.9, G73.6, L99.0, M14.3, M14.4, N16.3 |
|  | Other endocrine | E00, E03.0, E03.1, E07.1, E22.0, E23.0, E25, E26.8, E29.1, E31, E34.1, E34.2, E34.5, E34.8, G13.2, G73.5, Y42.1 |
|  | Other/ unspecific | E66, G63.3, G99.0, M14.5, N92*, Z86.3, Z93.8 |
|  | Renal/ GU | D63.8, G63.8, G99.8, I68.8, M90.8, N00, N01, N02, N03, N04, N05, N07, N08.4, N11, N12, N13, N14, N15, N16.0, N16.2, N16.4, N16.5, N16.8, N18, N19, N20, N21, N22, N23, N25, N26, N28, N29, N31, N32, N33.8, N35, N36, N39.1, N39.3, N39.4, N40, N41, N42, N70, N71, N72, N73, N74, N80, N81, N82, N85, N86*, N87, N88, P96.0, T82.4, T83.1, T83.2, T83.4, T83.5, T83.6, T83.8, T83.9, T85.5, T86.1, Y60.2, Y61.2, Y62.2, Y84.1, Z49, Z93.6, Z94.0, Z99.2 |
| Musculoskeletal/ skin | Chronic skin disorders | L10, L11.0, L11.8, L11.9, L12, L13, L14, L28, L40, L41, L42, L43, L44, L45, L57, L58.1, L59, L87, L88, L90, L92, L93, L95, L98.5, M09.0, Q80, Q81, Q87.0, Q87.1, Q87.2, Q87.3, Q87.4, Q87.5, Q89.4 |
|  | Congenital anomalies | Q18.8, Q65.0, Q65.1, Q65.2, Q65.8, Q65.9, Q67.5, Q68.2, Q68.3*, Q68.4*, Q68.5*, Q71, Q72, Q73, Q74, Q75.3, Q75.4, Q75.5, Q75.8, Q75.9, Q76.1, Q76.2, Q76.3, Q76.4, Q77, Q78, Q79.6, Q79.8, Q82.0, Q82.1, Q82.2, Q82.3, Q82.4, Q82.9, Q86.2, Q89.7, Q89.8, Q89.9 |
|  | Musculoskeletal/ connective tissue | G55.1, G55.2, G55.3, G63.5, G63.6, G73.7, J99.0, J99.1, L62.0, M05, M06, M07.0, M07.1, M07.2, M07.3, M07.6, M08, M09.8, M10, M11, M12, M13, M14.0, M14.6, M14.8, M30, M31, M32, M33, M34, M35, M40, M41, M42, M43, M45, M46, M47, M48, M50, M51, M53, M54, M60, M61, M62, M63.8, M80.1, M80.2, M80.3, M80.4, M80.5, M80.8, M80.9, M81.1, M81.2, M81.3, M81.4, M81.5, M81.6, M81.8, M81.9, M82.1, M82.8, M84.0, M84.1, M84.2, M84.8, M84.9, M85, M86.3, M86.4, M86.5, M86.6, M89, M90.0, M91, M92, M93, M94, N08.5, Y45.4 |
|  | Skeletal injuries/ amputations | S13*, S22.0*, S22.1*, S22.2*, S22.5*, S23*, S32*, S33*, S68.3*, S68.4*, S68.8*, S77*, S78*, S87*, S88*, S97*, S98.0*, S98.2*, S98.3*, S98.4*, T02*, T04*, T05*, T20.3*, T20.7*, T21.3*, T21.7*, T22.3*, T22.7*, T23.2*, T23.3*, T23.6*, T23.7*, T24.3*, T24.7*, T25.2*, T25.3*, T25.6*, T25.7*, T29.3*, T29.7*, T30.3*, T30.7*, T31.2*, T31.3*, T31.4*, T31.5*, T31.6*, T31.7*, T31.8*, T31.9*, T32.2*, T32.3*, T32.4*, T32.5*, T32.6*, T32.7*, T32.8*, T32.9*, T87.3*, T87.4*, T87.5*, T87.6*, T91.2*, T91.8*, T92.6*, T93.1*, T93.4*, T93.6*, T94.0*, T94.1*, T95.0*, T95.1*, T95.4*, T95.8*, T95.9*, Y83.5, Z89.1, Z89.2, Z89.5, Z89.6, Z89.7, Z89.8, Z97.1 |
| Neurological | Cerebral palsy | G80, G81, G82, G83 |
|  | Chronic ear conditions | H60.2, H65.2, H65.3, H65.4, H66.1, H66.2, H66.3, H69.0, H70.1, H73.1, H74.0, H74.1, H74.2, H74.3, H75.0, H80, H81.0, H81.4, H83.0, H83.2, H90.0, H90.3, H90.5, H90.6, H91, Z45.3 |
|  | Chronic eye conditions | H05.1, H05.2, H05.3, H05.4, H05.5, H05.8, H05.9, H13.3, H17, H18, H19.3, H19.8, H21, H26, H27, H28.0, H28.1, H28.2, H31, H32.8, H33, H34, H35, H40, H42.0, H43, H44, H47, H54.0, H54.1, H54.2, H54.4, T85.2, T85.3, Z44.2 |
|  | Congenital anomalies of neurological or sensory systems | Q00, Q01, Q02, Q03, Q04, Q05, Q06, Q07, Q10.4, Q10.7, Q11, Q12, Q13.0, Q13.1, Q13.2, Q13.3, Q13.4, Q13.8, Q13.9, Q14, Q15, Q16, Q75.0, Q75.1, Q85, Q86.0, Q86.1, Q86.8, Q90, Q91, Q92, Q93, Q95.2, Q95.3, Q97, Q99 |
|  | Epilepsy | F80.3, G40.0, G40.1, G40.2, G40.3, G40.4, G40.6, G40.7, G40.8, G40.9, G41, R56.8, Y46.0, Y46.1, Y46.2, Y46.3, Y46.4, Y46.5, Y46.6 |
|  | Injuries of brain, nerves, eyes or ears | S05*, S06*, S07*, S08*, S12*, S14*, S24*, S34*, S44*, S54*, S64*, S74*, S84*, S94*, T06.0*, T06.1*, T06.2*, T26*, T90.4*, T90.5*, T91.1*, T91.3*, T92.4* |
|  | Other | F02.2, F02.3, G00, G01, G02, G03, G04, G05, G06, G07, G08, G09, G10, G11, G12, G13.8, G14, G20, G21, G22, G23, G24.1, G24.2, G24.3, G24.4, G24.5, G24.8, G24.9, G25, G26, G30, G31.0, G31.1, G31.8, G31.9, G32, G35, G36, G37, G43, G44, G45, G46, G47.0, G47.1, G47.2, G47.4, G47.8, G47.9, G50, G51, G52, G53.0, G53.1, G53.8, G54, G55.8, G56, G57, G58, G59.8, G60, G61, G62.0, G62.2, G62.8, G62.9, G64, G70, G71, G72.2, G72.3, G72.4, G72.8, G72.9, G73.0, G73.3, G90, G91, G92, G93, G94.2, G94.8, G95, G96, G98, G99.1, G99.2, I60, I61, I62, I63, I64, I65, I66, I67, I68.0, I68.2, I69, I72.0, I72.5, T85.0, T85.1, Y46.7, Y46.8, Z98.2 |
|  | Perinatal conditions | P10, P21.0, P52, P57, P90, P91.1, P91.2, P91.6 |
| Non-specific | Non-specific | R62, R63.3, Z43.1, Z51.5, Z75.5, Z93.1, Z99.3 |
| Respiratory | Asthma | J41, J42, J43, J44, J45, J46, J47 |
|  | Congenital anomalies | Q30, Q31, Q32, Q33, Q34, Q35, Q36, Q37, Q79.0 |
|  | Cystic fibrosis | E84, P75 |
|  | Injuries | S17*, S27*, S28*, T27*, T91.4* |
|  | Other | G47.3, J60, J61, J62, J63, J64, J65, J66, J67, J68, J69, J70, J80, J81, J82, J84, J85, J86, J96.1, J98, P27, Y55.6, Z43.0, Z93.0, Z94.2 |

*Additional inclusion criteria: length of stay of hospital admission mentioning code >3 days; **Codes excluded in this study as requires age at admission to be 10 years or older

**Table S3.** Field names, descriptions and cleaning rules for key variables in this study

| Variable assignment in this study | Variable name in manuscript / code | Definition (values) | Origin dataset; variable name(s) in origin dataset | Cleaning rules |
| --- | --- | --- | --- | --- |
| Outcome | SEND provision / SEN_provision | Categorical indicator of assigned SEND provision split into no SEND provision (value N), SEND support (including School Action School Action Plus and SEND Support, values A, P & K), EHCP (including Statement of SEND and EHCP, values S, E or e) | NPD school censuses (pupil level); SENprovision/ SENprovision |  |
| Inclusion criteria | Year of school / NCyearActual | Categorical | NPD school censuses (pupil level); NCyearActual* AgeAtStartOfAcademicYear* | Record kept if NCyearActual="1" OR AgeAtStartOfAcademicYear>=5 & AgeAtStartOfAcademicYear <=6 & NCyearActual=="X” |
| Clustering variable | Local authority / LEA_code | Categorical indicator of local authority of residence in Year 1. We used residential MSOA rather than school MSOA because the LA where the pupil resides that is responsible for securing provision and providing top up funding associated with SEND provision.^16^ We used 2021 LA classifications to ensure geographical consistency in cases of LA mergers and boundary redrawing. | NPD school censuses; MSOA* | The equivalent LA in 2021 created from MSOA01 and MSOA11 |
| Covariate– individual level | Gestational age / gestat | Ordinal (groups specified in manuscript) | HES APC (birth record enhanced from maternal delivery record where missing); gestat_1 | Set to missing if <24 or >43 weeks |
| Covariate– individual level | Chronic conditions / CC_cardio CC_chronic_infections CC_mental_health CC_metabolic_endocrine_etc CC_musculoskeletal_skin  CC_neurological CC_non_specific CC_respiratory | Set of binary variables (yes, no) indicating presence of a chronic condition by subtype. Defined by the ICD-10 codes listed in **Table S3** in any diagnosis field in a hospital admission record before the age of 5 years (i.e. entry into school year 1) | HES APC (birth record and readmissions); diag_01-diag_20 |  |
| Covariate– individual level | Year of birth / ydob | Categorical year of child’s birth split into academic years, i.e. from 1 September to 31 August | HES APC (birth record), NPD; admidate  YearOfBirth*, MonthOfBirth* | Excluded if year or month of birth not matching between HES APC and NPD. |
| Covariate– individual level | Age at year 1 start / age_year1 | Age at entry into Year 1 (continuous) | HES APC (birth record), NPD; admidate, AcademicYear* | Excluded if year or month of birth not matching between HES APC and NPD. Calculated as (1 September at year 1 start minus admission date of birth record (proxy for date of birth)) divided by 365.25, rounded to 2 decimal places. |
| Covariate– individual level | Maternal age / matage | Continuous maternal age at delivery (in whole years) | HES APC (birth record enhanced from maternal delivery record)**; matage | Pre-derived outside ECHILD via mother-baby linkage. Set to missing if <10 or >65 |
| Covariate– individual level | Child’s gender / gender | Binary child’s parent/self-reported gender in January census of Year 1 (only female or male options available) | NPD school censuses; Gender* |  |
| Covariate– individual level | IDACI | Ordinal (6 groups including missing) IDACI quintile of child’s residential address in January census of Year 1. IDACI is calculated as the proportion of all children aged 0 to 15 in an area living in income deprived families (defined by benefit and tax credit receipt) | NPD school censuses; idaci_2007_rank, idaci_2010_rank | Either 2007 or 2010 is used depending on what is available; we cannot convert to the same IDACI as geographical information at the lower layer super output area-level (or smaller) is unavailable in the dataset |
| Covariate– individual level | Free school meals eligibility / FSMeligible | Binary entitlement to free school meals (yes or no) in January census of Year 1, with eligibility based on benefits/low-earnings criteria | NPD school censuses; FSMeligible* |  |
| Covariate– individual level | Racial-ethnic*** group | Child’s racial-ethnic group aggregated from parent/self-reported ethnicity in school census records (Asian, Black, Chinese, mixed or multiple, White, other, missing) | NPD school censuses; EthnicGroupMajor* | Modal non-missing major ethnic group across all available school census records |
| Covariate– individual level | EAL | Categorical EAL (yes, no or unknown) in January census of Year 1 | NPD school censuses ; LanguageGroupMajor* |  |
| Covariate– individual level | Hospital presentations | Combined number of days of hospital admissions and emergency department attendances from birth to 31 August before entry into Year 1 (rate per 100,000 person-days) | HES APC, HES Accident & Emergency, NPD; admidate, disdate, arrivaldate_ae, AcademicYear | Log((days in contact with a hospital/follow-up time)+1)*100000,  where days in contact with a hospital includes an admission or emergency department attendance between birth and year 1 school start |
| Covariate– individual level | School governance / school_gov | Categorical type of school governance in January census of Year 1 (community, sponsor led academy, converter led academy, free school, voluntary aided, voluntary controlled) | GIAS/ opensource DfE data; Nftype, school_type, typeofestablishmentname | Harmonised over time to create consistent categories (the code used to clean opensource data be published in a subsequent paper) |
| Covariate– individual level | EYFSP score | Continuous combined score for English and mathematics | NPD EYFSP dataset | Standardised z-score within academic year |
| Covariate– LA level | Pupil population / headcount_census | Continuous number of pupils in the LA in Year 1 (within LA and year) | NPD school censuses |  |
| Covariate– LA level | Special school attendance % / school_school_per_LA | Continuous (percentage) | GIAS/ opensource DfE data/ NPD school censuses; instype, headcount_census | Calculated as the number of pupils in Year 1 in special school divided by headcount of all pupils in Year 1 (within LA and year) |
| Covariate– LA level | Maintained school attendance % / maintained_per_LA | Continuous (percentage) | GIAS/ opensource DfE data/ NPD school censuses; instype, headcount_census | Calculated as the number of pupils in Year 1 in maintained schools divided by headcount of all pupils in Year 1 (within LA and year) |
| Covariate– LA level | Academy school attendance % / academy_per_LA | Continuous (percentage) | GIAS/ opensource DfE data/ NPD school censuses; instype, headcount_census | Calculated as the number of pupils in Year 1 in academy schools divided by headcount of all pupils in Year 1 (within LA and year) |
| Covariate– LA level | FSM eligible % / FSMeligble | Continuous (percentage) | NPD school censuses; FSMeligible*, headcount_census | Calculated as the number of pupils in Year 1 eligible for FSMs divided by headcount of all pupils in Year 1 (within LA and year) |
| Covariate– LA level | IDACI mode / IDACI_mode | Ordinal (5 groups) | NPD school censuses; idaci_2007_rank, idaci_2010_rank, headcount_census | Calculated as the modal IDACI quintile of pupils in Year 1 (within LA and year). Linear interpolation was used to fill in missing values for two data points (using data in the years before and after the missing value occurred) |

DfE= Department for Education; EHCP=Education health and care plan, EAL= English as an additional language, EYFSP = Early years foundation stage profile, FSM = free school meals, GIAS=get information about schools, HES APC = hospital episode statistics admitted patient care, IDACI=income deprivation affecting children index, LA=local authority, SEND=special educational needs and disability, MSOA=middle layer super output area, NPD=national pupil database; *these variables are either prefixed “PRU_” or suffixed “_SPRYY”, where YY=year of school attendance depending on whether information is from the pupil referral unit or main school census; **Linkage between birth and delivery records occurred prior to ECHILD linkage; ***we use the term “ethnic-racial” group to highlight that this construct includes both race-based (e.g. White) and ethnicity-based (e.g. Chinese) identifiers; †only included in analyses comparing SEND support with no provision due to the collinear relationship between incomplete or missing EYFSP record and EHCPs


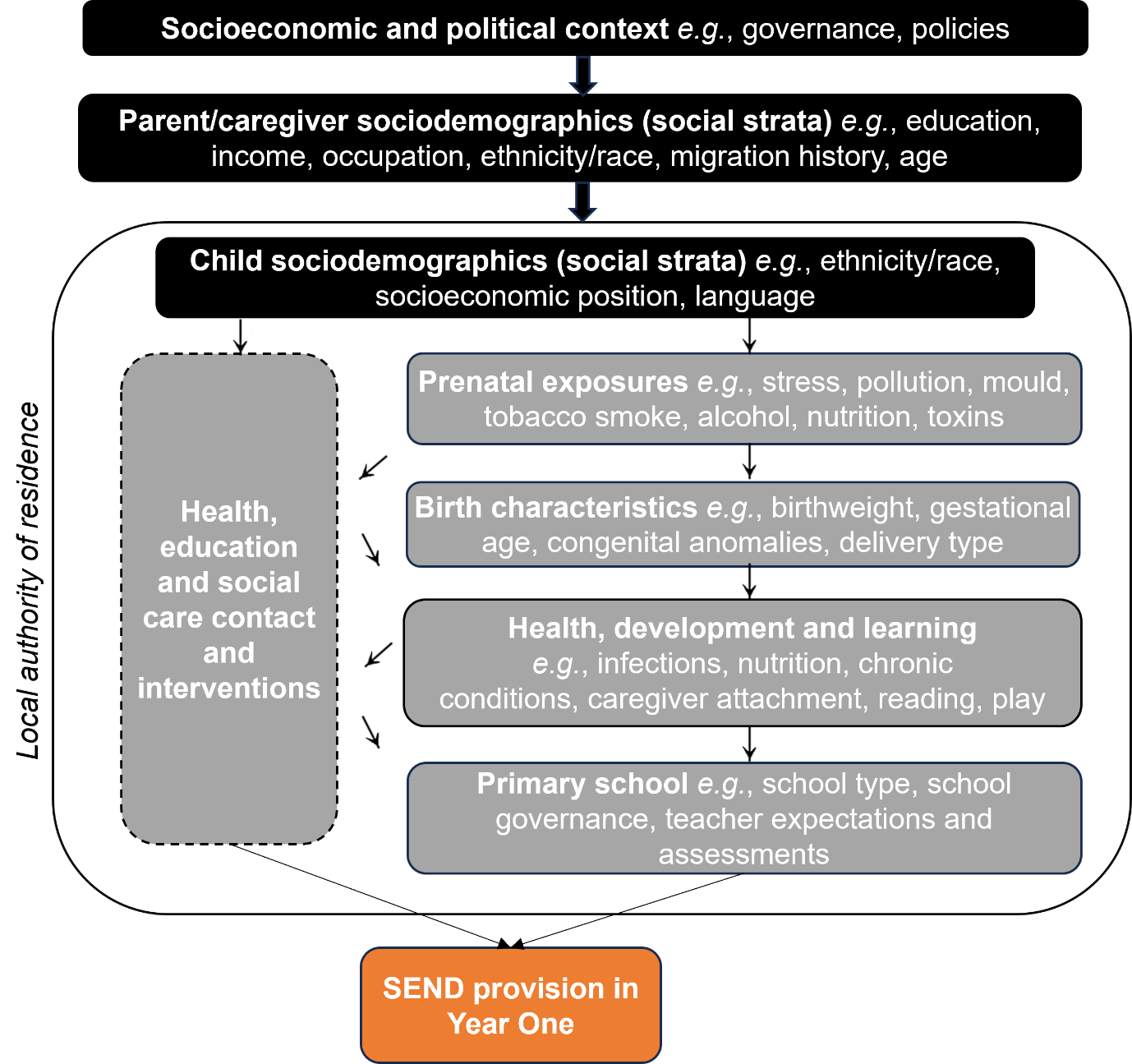


**Figure S1.** Conceptual framework: the association between local authority of residence and SEND provision in Year 1. Nodes represent variables and arrows represent associations; arrows indicate effect on all downstream variables; black boxes represent structural determinants; grey boxes are factors downstream from structural determinants; orange box is the outcome

**Box S1.** Changes to the analyses compared with the protocol

| Dataset  We did not use data from the alternative provision census because the Unique Reference Number (URN) is not available in this dataset. This did not affect the number of children in the dataset (i.e. all children with records in the alternative provision census at Year 1 also had a record in the school Census).  Subgroups  In response to reviewer comments on the assumption of children with major congenital anomalies requiring special educational needs and disability (SEND) provision and exploratory analysis showing small numbers of children in subtype specific major congenital anomaly groups (a method we planned to use to increase homogeneity of the population), we changed our population of interest. Instead of focusing on children with major congenital anomalies, we now look at all children, stratified by gestational age at birth and adjusted for early chronic conditions (alongside other factors). This change was made with the aim of reducing heterogeneity in our analyses, particularly at the lower end of gestational age, whilst being able to provide more complete population coverage.  Outcome  We changed the outcome to be SEND provision recorded at Year 1 only instead of reception to Year 2. By changing SEND provision to one point only, we aim to be more specific in our results (“early” identification). Relatedly, we can now include children born in 2012/13 (as enough follow up time before the COVID-19 pandemic is now available)  Covariates/other variables  We made a number of changes to the covariate selection based on exploratory analysis of the dataset (post protocol submission):   - We did not use child protection plan flag as an explanatory factor in our analyses because is not reliably available before 2013 - We combined special schools and alternative provision due to low numbers in alternative provision; did not include in final analyses due to collinearity with the outcome (almost all children in special school/alternative provision have recorded SEND provision) - We did not use Early Years Foundation Stage Profile (EYSFP) scores as a covariate in models as missing EYSFP leads to model overfitting (i.e. predicts SEND provision too accurately) and is temporally after education, health and care plan (EHCP) assignment in most cases - We did not use school type (type of school attended by child (special school, alternative provision or mainstream school) as it is co linear with the outcome. Almost all children in special school/alternative provision have recorded SEND provision - We added the English as an additional language (EAL) variable as an individual specific covariate - We added the percentage of pupils attending maintained schools as a local authority-level covariate - We reconsidered our covariate selection, guided by a conceptual diagram (Figure S1), leading to an additional sensitivity analysis where the analyses were rerun with a minimally adjusted covariate set.   Analysis  We changed our analysis plan to match the methods proposed by Merlo et al.(2016). These are more appropriate for describing contextual factors and for use with a binary outcome. |
| --- |


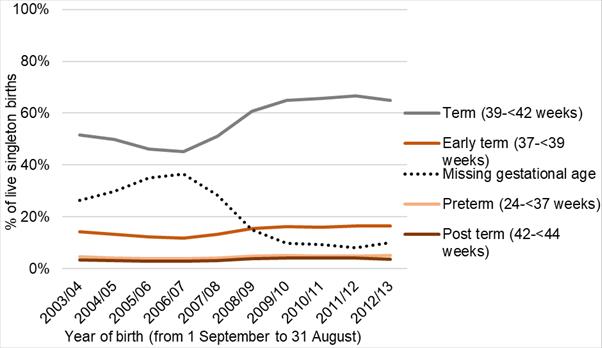


**Figure S2.** The proportion of live singleton births in hospital episode statistics with different gestational age groups, by year of birth


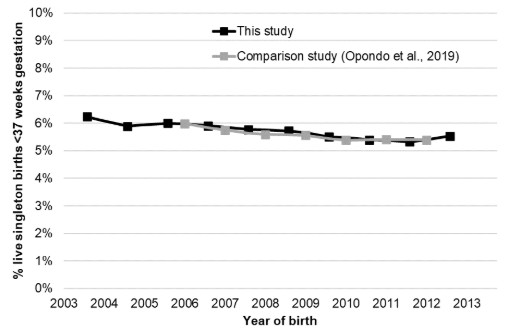


## **Figure S3.** Comparison between live singleton births <37 weeks gestation in this study* and comparison study**, by year of birth. *This study includes singleton births/deliveries in NHS hospitals in England between 2003/04 and 2012/13 (with years defined from 1 September to 31 August 2013) recorded in hospital episode statistics (HES); **Comparison study includes singleton births in England and Wales between 1 January 2005 and 31 December 2011 reported into National Health Service Numbers for Babies (NN4B) birth notifications system by the ONS (sourced from Opondo et al. (2019; <https://jech.bmj.com/content/74/4/336>) and licensed under CC BY 4.0)


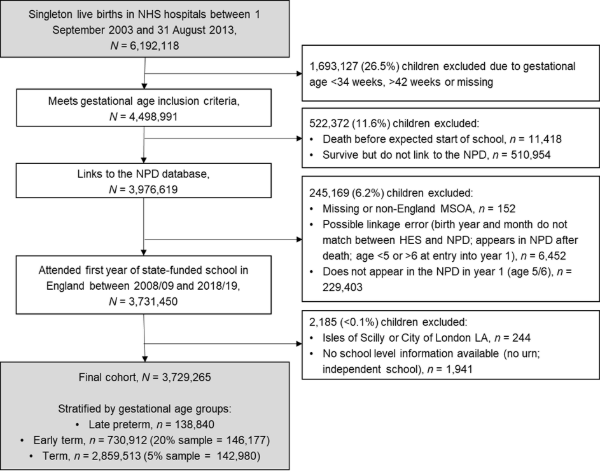


**Figure S4.** Flow chart of cohort derivation. LA = local authority; MSOA = middle layer super output area; NPD = national pupil database

**Table S4.** Single- and multi-level logistic regression analysis of (a) SEND support vs. no SEND provision, (b) EHCP vs. SEND support and (c) EHCP vs. no SEND provision: children born late preterm

| **(a) SEND support vs. no SEND provision** |  | **Step one (single level)** | **Step two (multilevel)** | **Step three (multilevel, + pupil headcount)** | **Step three (multilevel, + special school attendance %)** | **Step three (multilevel, + maintained school attendance %)** | **Step three (multilevel, + academy school attendance %)** | **Step three (multilevel, + FSM eligible %)** | **Step three (multilevel, + IDACI mode)** |
| --- | --- | --- | --- | --- | --- | --- | --- | --- | --- |
|  |  | **cOR (95% CI)** | **cOR (95% CI)** | **cOR (95% CI)** | **cOR (95% CI)** | **cOR (95% CI)** | **cOR (95% CI)** | **cOR (95% CI)** | **cOR (95% CI)** |
| *Child-specific associations* |  |  |  |  |  |  |  |  |  |
| Year of birth (1 September to 31 August) | 2003/04 | Reference | Reference | Reference | Reference | Reference | Reference | Reference | Reference |
|  | 2004/05 | 1.00 (0.93, 1.08) | 0.99 (0.92, 1.07) | 0.99 (0.92, 1.07) | 0.99 (0.91, 1.07) | 0.99 (0.92, 1.07) | 0.99 (0.92, 1.07) | 0.99 (0.92, 1.07) | 0.99 (0.92, 1.07) |
|  | 2005/06 | 0.87 (0.80, 0.93) | 0.87 (0.80, 0.94) | 0.87 (0.80, 0.94) | 0.87 (0.80, 0.94) | 0.87 (0.81, 0.95) | 0.87 (0.81, 0.95) | 0.87 (0.81, 0.94) | 0.87 (0.81, 0.94) |
|  | 2006/07 | 0.79 (0.73, 0.86) | 0.80 (0.74, 0.86) | 0.80 (0.74, 0.87) | 0.80 (0.73, 0.86) | 0.81 (0.75, 0.88) | 0.81 (0.75, 0.88) | 0.80 (0.74, 0.87) | 0.80 (0.74, 0.87) |
|  | 2007/08 | 0.70 (0.65, 0.76) | 0.71 (0.66, 0.77) | 0.71 (0.66, 0.77) | 0.70 (0.65, 0.76) | 0.73 (0.68, 0.79) | 0.73 (0.68, 0.79) | 0.71 (0.66, 0.77) | 0.71 (0.66, 0.77) |
|  | 2008/09 | 0.56 (0.52, 0.60) | 0.56 (0.52, 0.60) | 0.56 (0.52, 0.61) | 0.55 (0.51, 0.60) | 0.59 (0.54, 0.64) | 0.59 (0.54, 0.64) | 0.56 (0.52, 0.60) | 0.57 (0.52, 0.61) |
|  | 2009/10 | 0.53 (0.49, 0.57) | 0.54 (0.50, 0.58) | 0.54 (0.50, 0.58) | 0.53 (0.49, 0.57) | 0.56 (0.52, 0.61) | 0.56 (0.52, 0.61) | 0.53 (0.49, 0.57) | 0.54 (0.50, 0.58) |
|  | 2010/11 | 0.56 (0.52, 0.60) | 0.56 (0.52, 0.61) | 0.57 (0.53, 0.61) | 0.55 (0.51, 0.60) | 0.60 (0.55, 0.66) | 0.60 (0.55, 0.66) | 0.56 (0.51, 0.60) | 0.57 (0.53, 0.61) |
|  | 2011/12 | 0.52 (0.48, 0.56) | 0.51 (0.48, 0.56) | 0.52 (0.48, 0.56) | 0.50 (0.46, 0.55) | 0.56 (0.51, 0.61) | 0.56 (0.51, 0.61) | 0.51 (0.47, 0.55) | 0.52 (0.48, 0.56) |
|  | 2012/13 | 0.53 (0.50, 0.58) | 0.53 (0.49, 0.57) | 0.53 (0.49, 0.58) | 0.52 (0.48, 0.56) | 0.58 (0.53, 0.64) | 0.58 (0.53, 0.64) | 0.53 (0.49, 0.57) | 0.53 (0.50, 0.58) |
| Maternal age | Linear term | 1.00 (0.98, 1.02) | 1.00 (0.98, 1.02) | 1.00 (0.98, 1.02) | 1.00 (0.98, 1.02) | 1.00 (0.98, 1.02) | 1.00 (0.98, 1.02) | 1.00 (0.98, 1.02) | 1.00 (0.98, 1.02) |
|  | Quadratic term | 1.00 (1.00, 1.00) | 1.00 (1.00, 1.00) | 1.00 (1.00, 1.00) | 1.00 (1.00, 1.00) | 1.00 (1.00, 1.00) | 1.00 (1.00, 1.00) | 1.00 (1.00, 1.00) | 1.00 (1.00, 1.00) |
|  | Missing | 0.87 (0.61, 1.25) | 0.87 (0.60, 1.25) | 0.87 (0.60, 1.26) | 0.87 (0.60, 1.26) | 0.87 (0.60, 1.26) | 0.87 (0.60, 1.26) | 0.87 (0.60, 1.25) | 0.87 (0.61, 1.26) |
| Gender | Male vs. female (ref.) | 1.69 (1.64, 1.75) | 1.69 (1.63, 1.75) | 1.69 (1.63, 1.75) | 1.69 (1.63, 1.75) | 1.69 (1.63, 1.75) | 1.69 (1.63, 1.75) | 1.69 (1.63, 1.75) | 1.69 (1.63, 1.75) |
| Chronic condition (cancer/blood) | Yes vs. no (ref.) | 1.06 (0.92, 1.22) | 1.05 (0.91, 1.22) | 1.05 (0.91, 1.22) | 1.05 (0.91, 1.22) | 1.06 (0.91, 1.22) | 1.06 (0.91, 1.22) | 1.05 (0.91, 1.22) | 1.05 (0.91, 1.22) |
| Chronic condition (cardiovascular) | Yes vs. no (ref.) | 1.12 (1.00, 1.25) | 1.12 (1.00, 1.26) | 1.12 (1.00, 1.26) | 1.12 (1.00, 1.26) | 1.12 (1.00, 1.26) | 1.12 (1.00, 1.26) | 1.12 (1.00, 1.26) | 1.12 (1.00, 1.26) |
| Chronic condition (chronic infection) | Yes vs. no (ref.) | 1.30 (0.86, 1.97) | 1.24 (0.82, 1.88) | 1.24 (0.82, 1.88) | 1.24 (0.82, 1.88) | 1.24 (0.82, 1.88) | 1.24 (0.82, 1.88) | 1.24 (0.82, 1.88) | 1.24 (0.82, 1.88) |
| Chronic condition (mental health/developmental) | Yes vs. no (ref.) | 3.82 (3.27, 4.47) | 3.87 (3.31, 4.53) | 3.87 (3.31, 4.53) | 3.87 (3.31, 4.53) | 3.87 (3.31, 4.53) | 3.87 (3.31, 4.53) | 3.87 (3.31, 4.53) | 3.87 (3.31, 4.53) |
| Chronic condition (metabolic endocrine) | Yes vs. no (ref.) | 1.09 (1.02, 1.17) | 1.09 (1.02, 1.17) | 1.09 (1.02, 1.17) | 1.09 (1.02, 1.17) | 1.10 (1.02, 1.17) | 1.10 (1.02, 1.17) | 1.09 (1.02, 1.17) | 1.09 (1.02, 1.17) |
| Chronic condition (musculoskeletal skin) | Yes vs. no (ref.) | 1.42 (1.25, 1.60) | 1.45 (1.28, 1.64) | 1.45 (1.28, 1.64) | 1.45 (1.28, 1.64) | 1.45 (1.28, 1.64) | 1.45 (1.28, 1.64) | 1.45 (1.28, 1.64) | 1.45 (1.27, 1.64) |
| Chronic condition (neurological) | Yes vs. no (ref.) | 1.82 (1.70, 1.95) | 1.83 (1.71, 1.96) | 1.83 (1.71, 1.96) | 1.83 (1.71, 1.96) | 1.83 (1.71, 1.95) | 1.83 (1.70, 1.95) | 1.83 (1.71, 1.96) | 1.83 (1.71, 1.96) |
| Chronic condition (non-specific) | Yes vs. no (ref.) | 1.38 (1.25, 1.51) | 1.39 (1.26, 1.53) | 1.39 (1.26, 1.53) | 1.39 (1.26, 1.53) | 1.39 (1.26, 1.53) | 1.39 (1.27, 1.53) | 1.39 (1.26, 1.53) | 1.39 (1.26, 1.53) |
| Chronic condition (respiratory) | Yes vs. no (ref.) | 1.19 (1.11, 1.27) | 1.19 (1.11, 1.28) | 1.19 (1.11, 1.28) | 1.19 (1.11, 1.28) | 1.19 (1.11, 1.28) | 1.19 (1.11, 1.28) | 1.19 (1.11, 1.28) | 1.19 (1.11, 1.28) |
| IDACI | 1 Most deprived 20% | 1.29 (1.21, 1.38) | 1.32 (1.23, 1.41) | 1.32 (1.23, 1.41) | 1.32 (1.23, 1.41) | 1.32 (1.23, 1.41) | 1.32 (1.23, 1.41) | 1.33 (1.24, 1.42) | 1.33 (1.24, 1.42) |
|  | 2 | 1.22 (1.14, 1.30) | 1.23 (1.15, 1.31) | 1.23 (1.15, 1.31) | 1.23 (1.15, 1.31) | 1.23 (1.15, 1.31) | 1.23 (1.15, 1.31) | 1.23 (1.15, 1.31) | 1.23 (1.15, 1.32) |
|  | 3 | 1.15 (1.08, 1.23) | 1.13 (1.06, 1.21) | 1.13 (1.06, 1.21) | 1.13 (1.06, 1.21) | 1.13 (1.06, 1.22) | 1.13 (1.06, 1.22) | 1.14 (1.06, 1.22) | 1.14 (1.06, 1.22) |
|  | 4 | 1.03 (0.96, 1.11) | 1.03 (0.96, 1.11) | 1.03 (0.96, 1.11) | 1.03 (0.96, 1.11) | 1.03 (0.96, 1.11) | 1.03 (0.96, 1.11) | 1.03 (0.96, 1.11) | 1.03 (0.96, 1.11) |
|  | 5 Least deprived 20% | Reference | Reference | Reference | Reference | Reference | Reference | Reference | Reference |
|  | Missing | 1.19 (0.32, 4.43) | 1.33 (0.35, 5.05) | 1.33 (0.35, 5.05) | 1.33 (0.35, 5.08) | 1.33 (0.35, 5.02) | 1.33 (0.35, 5.02) | 1.33 (0.35, 5.06) | 1.34 (0.35, 5.09) |
| FSM eligible | Yes vs. no (ref.) | 1.35 (1.30, 1.41) | 1.35 (1.30, 1.41) | 1.35 (1.30, 1.41) | 1.35 (1.30, 1.41) | 1.35 (1.30, 1.41) | 1.35 (1.30, 1.41) | 1.35 (1.30, 1.41) | 1.35 (1.30, 1.41) |
| Racial-ethnic group | Asian | 0.83 (0.78, 0.89) | 0.82 (0.76, 0.88) | 0.82 (0.76, 0.88) | 0.82 (0.76, 0.88) | 0.82 (0.76, 0.88) | 0.82 (0.76, 0.88) | 0.82 (0.76, 0.88) | 0.82 (0.76, 0.88) |
|  | Black | 1.06 (0.98, 1.15) | 0.95 (0.88, 1.04) | 0.95 (0.88, 1.04) | 0.95 (0.88, 1.04) | 0.95 (0.88, 1.03) | 0.95 (0.88, 1.03) | 0.95 (0.88, 1.04) | 0.95 (0.88, 1.04) |
|  | Chinese | 0.68 (0.47, 0.97) | 0.66 (0.46, 0.94) | 0.66 (0.46, 0.94) | 0.66 (0.46, 0.94) | 0.66 (0.46, 0.94) | 0.65 (0.46, 0.94) | 0.66 (0.46, 0.94) | 0.66 (0.46, 0.94) |
|  | Mixed | 0.95 (0.88, 1.02) | 0.90 (0.84, 0.97) | 0.90 (0.84, 0.97) | 0.90 (0.84, 0.97) | 0.90 (0.84, 0.97) | 0.90 (0.84, 0.97) | 0.90 (0.84, 0.97) | 0.90 (0.84, 0.97) |
|  | Other | 0.98 (0.85, 1.14) | 0.87 (0.75, 1.02) | 0.87 (0.75, 1.02) | 0.87 (0.75, 1.02) | 0.87 (0.75, 1.02) | 0.87 (0.75, 1.02) | 0.87 (0.75, 1.02) | 0.87 (0.75, 1.02) |
|  | White | Reference | Reference | Reference | Reference | Reference | Reference | Reference | Reference |
|  | Missing | 0.99 (0.71, 1.39) | 0.96 (0.68, 1.34) | 0.96 (0.68, 1.34) | 0.96 (0.68, 1.34) | 0.96 (0.68, 1.34) | 0.96 (0.68, 1.34) | 0.96 (0.68, 1.34) | 0.96 (0.68, 1.34) |
| EAL | No | Reference | Reference | Reference | Reference | Reference | Reference | Reference | Reference |
|  | Yes | 0.72 (0.68, 0.77) | 0.69 (0.65, 0.74) | 0.69 (0.65, 0.74) | 0.69 (0.65, 0.74) | 0.69 (0.65, 0.74) | 0.69 (0.65, 0.74) | 0.69 (0.65, 0.74) | 0.69 (0.65, 0.74) |
|  | Unclear or missing | 0.73 (0.53, 1.01) | 0.70 (0.51, 0.97) | 0.70 (0.51, 0.97) | 0.70 (0.51, 0.97) | 0.71 (0.51, 0.97) | 0.71 (0.51, 0.97) | 0.71 (0.51, 0.97) | 0.70 (0.51, 0.97) |
| Rate of hospitalisation (pre year 1) |  | 1.01 (1.01, 1.01) | 1.01 (1.01, 1.01) | 1.01 (1.01, 1.01) | 1.01 (1.01, 1.01) | 1.01 (1.01, 1.01) | 1.01 (1.01, 1.01) | 1.01 (1.01, 1.01) | 1.01 (1.01, 1.01) |
| Age at Year One start |  | 1.21 (1.14, 1.28) | 1.23 (1.16, 1.31) | 1.23 (1.16, 1.31) | 1.23 (1.16, 1.31) | 1.23 (1.16, 1.31) | 1.23 (1.16, 1.31) | 1.23 (1.16, 1.31) | 1.23 (1.16, 1.31) |
| School governance | Community | Reference | Reference | Reference | Reference | Reference | Reference | Reference | Reference |
|  | Sponsor led academy | 0.90 (0.84, 0.98) | 0.91 (0.84, 0.99) | 0.91 (0.84, 0.99) | 0.91 (0.84, 0.99) | 0.93 (0.85, 1.00) | 0.93 (0.85, 1.01) | 0.91 (0.84, 0.99) | 0.91 (0.84, 0.99) |
|  | Converter led academy | 0.97 (0.91, 1.03) | 0.99 (0.93, 1.05) | 0.99 (0.93, 1.05) | 0.99 (0.93, 1.05) | 1.01 (0.95, 1.08) | 1.01 (0.95, 1.08) | 0.99 (0.93, 1.05) | 0.99 (0.93, 1.05) |
|  | Free school | 0.89 (0.69, 1.15) | 0.87 (0.67, 1.12) | 0.87 (0.67, 1.12) | 0.87 (0.67, 1.12) | 0.87 (0.68, 1.12) | 0.87 (0.67, 1.12) | 0.87 (0.67, 1.12) | 0.87 (0.67, 1.12) |
|  | Voluntary aided | 0.89 (0.84, 0.93) | 0.89 (0.85, 0.94) | 0.89 (0.85, 0.94) | 0.89 (0.85, 0.94) | 0.89 (0.85, 0.94) | 0.89 (0.85, 0.94) | 0.89 (0.85, 0.94) | 0.89 (0.85, 0.94) |
|  | Voluntary controlled | 0.89 (0.83, 0.95) | 0.89 (0.83, 0.95) | 0.89 (0.83, 0.95) | 0.89 (0.83, 0.95) | 0.89 (0.83, 0.95) | 0.89 (0.83, 0.95) | 0.89 (0.83, 0.95) | 0.89 (0.83, 0.95) |
| Missing EYSFSP score | Yes vs. no (ref.) | 2.63 (2.29, 3.02) | 2.64 (2.30, 3.04) | 2.64 (2.30, 3.04) | 2.64 (2.30, 3.04) | 2.65 (2.30, 3.04) | 2.65 (2.30, 3.04) | 2.64 (2.30, 3.04) | 2.65 (2.30, 3.04) |
| Standardised EYSFP score |  | 0.25 (0.24, 0.26) | 0.24 (0.24, 0.25) | 0.24 (0.24, 0.25) | 0.24 (0.24, 0.25) | 0.24 (0.24, 0.25) | 0.24 (0.24, 0.25) | 0.24 (0.24, 0.25) | 0.24 (0.24, 0.25) |
| _cons |  | 0.02 (0.01, 0.03) | 0.02 (0.01, 0.03) | 0.02 (0.01, 0.03) | 0.02 (0.01, 0.03) | 0.01 (0.01, 0.02) | 0.02 (0.01, 0.03) | 0.02 (0.01, 0.03) | 0.02 (0.01, 0.03) |
| *LA-specific associations* | |  |  |  |  |  |  |  |  |
| Pupil population | Linear term |  |  | 1.00 (1.00, 1.00) |  |  |  |  |  |
|  | Quadratic term |  |  | 1.00 (1.00, 1.00) |  |  |  |  |  |
| Special school attendance | Linear term |  |  |  | 0.98 (0.74, 1.31) |  |  |  |  |
|  | Quadratic term |  |  |  | 1.07 (0.91, 1.27) |  |  |  |  |
| Maintained school attendance | Linear term |  |  |  |  | 1.00 (1.00, 1.01) |  |  |  |
| Academy attendance | Linear term |  |  |  |  |  | 1.00 (0.99, 1.00) |  |  |
| FSM eligible | Linear term |  |  |  |  |  |  | 0.99 (0.97, 1.01) |  |
|  | Quadratic term |  |  |  |  |  |  | 1.00 (1.00, 1.00) |  |
| IDACI mode groups | 1 Most deprived 20% |  |  |  |  |  |  |  | 0.89 (0.79, 1.00) |
|  | 2 |  |  |  |  |  |  |  | 0.88 (0.77, 0.99) |
|  | 3 |  |  |  |  |  |  |  | 0.88 (0.76, 1.02) |
|  | 4 |  |  |  |  |  |  |  | 0.88 (0.77, 1.01) |
|  | 5 Least deprived 20% |  |  |  |  |  |  |  | Reference |
| *LA-general effects* |  |  |  |  |  |  |  |  |  |
| LA variance (95% CI) |  |  | 0.08 (0.06, 0.10) | 0.08 (0.06, 0.10) | 0.08 (0.06, 0.10) | 0.08 (0.06, 0.10) | 0.08 (0.06, 0.10) | 0.07 (0.06, 0.10) | 0.08 (0.06, 0.11) |
| PCV* |  |  |  | -0.90% | 0.31% | 0.77% | 1.13% | 1.75% | -5.26% |
| AUC (95% CI) |  | 0.84 (0.84, 0.85) | 0.85 (0.85, 0.85) | 0.85 (0.85, 0.85) | 0.85 (0.85, 0.85) | 0.85 (0.85, 0.85) | 0.85 (0.85, 0.85) | 0.85 (0.85, 0.85) | 0.85 (0.85, 0.85) |
| AUC change* |  |  | 0.004 | 0.000 | 0.000 | 0.000 | 0.000 | 0.000 | 0.000 |
| ICC % (95% CI) |  |  | 2.26 (1.73, 2.95) | 2.28 (1.74, 2.98) | 2.26 (1.72, 2.95) | 2.24 (1.72, 2.93) | 2.24 (1.71, 2.92) | 2.22 (1.70, 2.90) | 2.38 (1.81, 3.12) |
| *Model parameters/goodness of fit* |  |  |  |  |  |  |  |  |  |
| N |  | 134894 | 134894 | 134894 | 134894 | 134894 | 134894 | 134894 | 134894 |
| Log likelihood |  | -45302.2 | -44926.1 | -44925.9 | -44924.0 | -44922.0 | -44921.6 | -44925.4 | -44923.0 |
| Degrees of freedom |  | 46 | 47 | 49 | 49 | 48 | 48 | 49 | 51 |
| AIC |  | 90696.5 | 89946.3 | 89949.7 | 89946.0 | 89940.0 | 89939.2 | 89948.9 | 89947.9 |
| AIC change* |  |  | -750.2 | 3.4 | -0.3 | -6.3 | -7.1 | 2.6 | 1.6 |
| BIC |  | 91147.9 | 90407.4 | 90430.5 | 90426.8 | 90411.0 | 90410.2 | 90429.7 | 90448.3 |
| BIC change* |  | Reference | Reference | Reference | Reference | Reference | Reference | Reference | Reference |
| **(b) EHCP vs. SEND support** |  | **Step one (single level** | **Step two (multilevel)** | **Step three (multilevel, + pupil headcount)** | **Step three (multilevel, + special school attendance %)** | **Step three (multilevel, + maintained school attendance %)** | **Step three (multilevel, + academy school attendance %)** | **Step three (multilevel, + FSM eligible %)** | **Step three (multilevel, + IDACI mode)** |
|  |  | **cOR (95% CI)** | **cOR (95% CI)** | **cOR (95% CI)** | **cOR (95% CI)** | **cOR (95% CI)** | **cOR (95% CI)** | **cOR (95% CI)** | **cOR (95% CI)** |
| *Child-specific associations* |  |  |  |  |  |  |  |  |  |
| Year of birth (1 September to 31 August) | 2003/04 | Reference | Reference | Reference | Reference | Reference | Reference | Reference | Reference |
|  | 2004/05 | 1.10 (0.91, 1.33) | 1.08 (0.89, 1.31) | 1.09 (0.90, 1.32) | 1.06 (0.87, 1.29) | 1.08 (0.89, 1.31) | 1.08 (0.89, 1.31) | 1.10 (0.91, 1.34) | 1.09 (0.90, 1.33) |
|  | 2005/06 | 1.10 (0.90, 1.33) | 1.09 (0.89, 1.32) | 1.09 (0.90, 1.33) | 1.07 (0.88, 1.31) | 1.08 (0.88, 1.31) | 1.08 (0.88, 1.31) | 1.12 (0.92, 1.36) | 1.10 (0.90, 1.34) |
|  | 2006/07 | 1.06 (0.87, 1.30) | 1.05 (0.86, 1.28) | 1.06 (0.86, 1.29) | 1.04 (0.85, 1.27) | 1.02 (0.83, 1.25) | 1.02 (0.84, 1.25) | 1.07 (0.88, 1.31) | 1.06 (0.87, 1.30) |
|  | 2007/08 | 1.23 (1.02, 1.49) | 1.22 (1.01, 1.47) | 1.23 (1.01, 1.48) | 1.13 (0.93, 1.37) | 1.16 (0.95, 1.41) | 1.17 (0.96, 1.42) | 1.22 (1.01, 1.48) | 1.24 (1.02, 1.50) |
|  | 2008/09 | 1.30 (1.08, 1.56) | 1.31 (1.09, 1.57) | 1.32 (1.09, 1.59) | 1.18 (0.98, 1.42) | 1.22 (1.01, 1.49) | 1.23 (1.01, 1.50) | 1.29 (1.07, 1.56) | 1.32 (1.10, 1.60) |
|  | 2009/10 | 1.22 (1.02, 1.47) | 1.23 (1.02, 1.47) | 1.24 (1.03, 1.49) | 1.09 (0.90, 1.32) | 1.13 (0.93, 1.39) | 1.14 (0.94, 1.40) | 1.15 (0.95, 1.40) | 1.25 (1.04, 1.50) |
|  | 2010/11 | 1.24 (1.04, 1.49) | 1.23 (1.03, 1.48) | 1.24 (1.03, 1.50) | 1.09 (0.90, 1.31) | 1.12 (0.91, 1.38) | 1.13 (0.92, 1.39) | 1.15 (0.95, 1.39) | 1.25 (1.04, 1.50) |
|  | 2011/12 | 1.40 (1.17, 1.67) | 1.40 (1.17, 1.69) | 1.41 (1.17, 1.70) | 1.20 (0.99, 1.45) | 1.25 (1.00, 1.55) | 1.26 (1.02, 1.57) | 1.31 (1.08, 1.58) | 1.42 (1.18, 1.71) |
|  | 2012/13 | 1.45 (1.22, 1.74) | 1.47 (1.23, 1.77) | 1.48 (1.23, 1.78) | 1.24 (1.03, 1.51) | 1.29 (1.02, 1.62) | 1.31 (1.04, 1.64) | 1.44 (1.20, 1.74) | 1.49 (1.24, 1.79) |
| Maternal age | Linear term | 0.98 (0.93, 1.03) | 0.99 (0.94, 1.04) | 0.99 (0.94, 1.04) | 0.98 (0.94, 1.03) | 0.99 (0.94, 1.04) | 0.99 (0.94, 1.04) | 0.99 (0.94, 1.04) | 0.99 (0.94, 1.04) |
|  | Quadratic term | 1.00 (1.00, 1.00) | 1.00 (1.00, 1.00) | 1.00 (1.00, 1.00) | 1.00 (1.00, 1.00) | 1.00 (1.00, 1.00) | 1.00 (1.00, 1.00) | 1.00 (1.00, 1.00) | 1.00 (1.00, 1.00) |
|  | Missing | 1.42 (0.62, 3.28) | 1.48 (0.63, 3.46) | 1.48 (0.63, 3.47) | 1.48 (0.63, 3.48) | 1.47 (0.63, 3.45) | 1.47 (0.63, 3.45) | 1.46 (0.62, 3.42) | 1.45 (0.62, 3.40) |
| Gender | Male vs. female (ref.) | 1.33 (1.22, 1.45) | 1.33 (1.22, 1.46) | 1.33 (1.22, 1.46) | 1.33 (1.22, 1.46) | 1.33 (1.22, 1.46) | 1.33 (1.22, 1.46) | 1.33 (1.22, 1.46) | 1.33 (1.22, 1.46) |
| Chronic condition (cancer/blood) | Yes vs. no (ref.) | 0.88 (0.72, 1.09) | 0.86 (0.69, 1.06) | 0.86 (0.69, 1.06) | 0.86 (0.69, 1.07) | 0.86 (0.69, 1.06) | 0.86 (0.69, 1.06) | 0.86 (0.69, 1.06) | 0.86 (0.69, 1.06) |
| Chronic condition (cardiovascular) | Yes vs. no (ref.) | 2.30 (1.99, 2.65) | 2.40 (2.08, 2.77) | 2.40 (2.08, 2.78) | 2.40 (2.08, 2.78) | 2.40 (2.07, 2.77) | 2.40 (2.07, 2.77) | 2.40 (2.07, 2.77) | 2.40 (2.07, 2.77) |
| Chronic condition (chronic infection) | Yes vs. no (ref.) | 1.15 (0.67, 1.98) | 1.12 (0.65, 1.95) | 1.13 (0.65, 1.95) | 1.12 (0.65, 1.94) | 1.12 (0.65, 1.94) | 1.12 (0.65, 1.94) | 1.13 (0.65, 1.95) | 1.13 (0.65, 1.95) |
| Chronic condition (mental health/developmental) | Yes vs. no (ref.) | 5.66 (5.01, 6.40) | 5.97 (5.27, 6.77) | 5.96 (5.26, 6.76) | 5.98 (5.28, 6.78) | 5.97 (5.27, 6.77) | 5.97 (5.27, 6.77) | 5.98 (5.28, 6.78) | 5.98 (5.27, 6.77) |
| Chronic condition (metabolic endocrine) | Yes vs. no (ref.) | 1.07 (0.95, 1.21) | 1.07 (0.94, 1.21) | 1.07 (0.94, 1.21) | 1.07 (0.94, 1.21) | 1.07 (0.94, 1.21) | 1.07 (0.94, 1.21) | 1.07 (0.94, 1.21) | 1.07 (0.94, 1.21) |
| Chronic condition (musculoskeletal skin) | Yes vs. no (ref.) | 1.47 (1.24, 1.74) | 1.48 (1.25, 1.76) | 1.48 (1.25, 1.76) | 1.48 (1.25, 1.76) | 1.48 (1.25, 1.76) | 1.48 (1.25, 1.76) | 1.48 (1.25, 1.76) | 1.48 (1.25, 1.76) |
| Chronic condition (neurological) | Yes vs. no (ref.) | 4.02 (3.67, 4.41) | 4.22 (3.84, 4.64) | 4.22 (3.84, 4.64) | 4.23 (3.84, 4.65) | 4.22 (3.84, 4.64) | 4.22 (3.84, 4.64) | 4.22 (3.84, 4.64) | 4.22 (3.84, 4.64) |
| Chronic condition (non-specific) | Yes vs. no (ref.) | 2.44 (2.17, 2.75) | 2.52 (2.23, 2.85) | 2.52 (2.24, 2.85) | 2.52 (2.23, 2.85) | 2.52 (2.23, 2.85) | 2.52 (2.23, 2.85) | 2.52 (2.23, 2.85) | 2.52 (2.23, 2.84) |
| Chronic condition (respiratory) | Yes vs. no (ref.) | 0.75 (0.66, 0.85) | 0.74 (0.65, 0.84) | 0.74 (0.65, 0.84) | 0.74 (0.65, 0.84) | 0.74 (0.65, 0.84) | 0.74 (0.65, 0.84) | 0.74 (0.65, 0.84) | 0.74 (0.65, 0.84) |
| IDACI | 1 Most deprived 20% | 0.64 (0.55, 0.74) | 0.72 (0.61, 0.84) | 0.71 (0.61, 0.83) | 0.70 (0.60, 0.82) | 0.72 (0.61, 0.84) | 0.72 (0.61, 0.84) | 0.74 (0.63, 0.87) | 0.73 (0.63, 0.86) |
|  | 2 | 0.79 (0.68, 0.91) | 0.83 (0.71, 0.96) | 0.82 (0.71, 0.96) | 0.82 (0.70, 0.96) | 0.83 (0.71, 0.96) | 0.83 (0.71, 0.96) | 0.84 (0.72, 0.98) | 0.84 (0.72, 0.98) |
|  | 3 | 0.84 (0.72, 0.98) | 0.86 (0.73, 1.01) | 0.86 (0.73, 1.01) | 0.86 (0.73, 1.01) | 0.86 (0.73, 1.01) | 0.86 (0.73, 1.01) | 0.87 (0.74, 1.01) | 0.87 (0.74, 1.02) |
|  | 4 | 1.00 (0.85, 1.17) | 1.01 (0.86, 1.19) | 1.01 (0.86, 1.19) | 1.01 (0.86, 1.19) | 1.01 (0.86, 1.19) | 1.01 (0.86, 1.19) | 1.02 (0.86, 1.20) | 1.02 (0.86, 1.20) |
|  | 5 Least deprived 20% | Reference | Reference | Reference | Reference | Reference | Reference | Reference | Reference |
|  | Missing | 1.60 (0.18, 14.51) | 2.26 (0.25, 20.55) | 2.19 (0.24, 19.94) | 2.26 (0.25, 20.83) | 2.24 (0.25, 20.40) | 2.24 (0.25, 20.42) | 2.29 (0.25, 20.85) | 2.24 (0.25, 20.40) |
| FSM eligible | Yes vs. no (ref.) | 0.95 (0.86, 1.03) | 0.94 (0.86, 1.03) | 0.94 (0.86, 1.03) | 0.94 (0.86, 1.03) | 0.94 (0.86, 1.03) | 0.94 (0.86, 1.03) | 0.95 (0.86, 1.04) | 0.94 (0.86, 1.03) |
| Racial-ethnic group | Asian | 1.46 (1.25, 1.70) | 1.52 (1.29, 1.79) | 1.52 (1.29, 1.79) | 1.50 (1.28, 1.77) | 1.52 (1.29, 1.79) | 1.52 (1.29, 1.79) | 1.53 (1.30, 1.80) | 1.52 (1.29, 1.79) |
|  | Black | 1.73 (1.46, 2.05) | 1.84 (1.53, 2.21) | 1.83 (1.53, 2.20) | 1.84 (1.53, 2.20) | 1.84 (1.54, 2.21) | 1.85 (1.54, 2.22) | 1.87 (1.56, 2.25) | 1.86 (1.55, 2.23) |
|  | Chinese | 4.22 (2.32, 7.69) | 4.10 (2.23, 7.55) | 4.11 (2.23, 7.56) | 4.09 (2.22, 7.54) | 4.12 (2.24, 7.58) | 4.11 (2.23, 7.57) | 4.15 (2.25, 7.64) | 4.09 (2.22, 7.54) |
|  | Mixed | 1.31 (1.11, 1.55) | 1.32 (1.11, 1.56) | 1.32 (1.11, 1.56) | 1.32 (1.12, 1.56) | 1.32 (1.12, 1.56) | 1.32 (1.12, 1.56) | 1.32 (1.12, 1.57) | 1.32 (1.12, 1.56) |
|  | Other | 1.11 (0.77, 1.58) | 1.06 (0.73, 1.53) | 1.05 (0.73, 1.52) | 1.05 (0.73, 1.52) | 1.06 (0.73, 1.53) | 1.06 (0.73, 1.53) | 1.07 (0.74, 1.55) | 1.07 (0.74, 1.55) |
|  | White | Reference | Reference | Reference | Reference | Reference | Reference | Reference | Reference |
|  | Missing | 1.91 (1.06, 3.45) | 1.85 (1.02, 3.36) | 1.84 (1.01, 3.34) | 1.88 (1.03, 3.41) | 1.87 (1.03, 3.39) | 1.86 (1.03, 3.38) | 1.86 (1.02, 3.37) | 1.85 (1.02, 3.36) |
| EAL | No | Reference | Reference | Reference | Reference | Reference | Reference | Reference | Reference |
|  | Yes | 0.99 (0.86, 1.14) | 0.97 (0.84, 1.13) | 0.97 (0.84, 1.13) | 0.98 (0.85, 1.13) | 0.98 (0.84, 1.13) | 0.98 (0.84, 1.13) | 0.97 (0.84, 1.13) | 0.98 (0.85, 1.13) |
|  | Unclear or missing | 1.03 (0.50, 2.13) | 1.18 (0.58, 2.41) | 1.18 (0.58, 2.40) | 1.19 (0.59, 2.41) | 1.19 (0.58, 2.41) | 1.19 (0.58, 2.41) | 1.19 (0.59, 2.43) | 1.19 (0.59, 2.43) |
| Rate of hospitalisation (pre year 1) |  | 1.00 (1.00, 1.01) | 1.01 (1.00, 1.01) | 1.01 (1.00, 1.01) | 1.01 (1.00, 1.01) | 1.01 (1.00, 1.01) | 1.01 (1.00, 1.01) | 1.01 (1.00, 1.01) | 1.01 (1.00, 1.01) |
| Age at Year One start |  | 1.77 (1.54, 2.03) | 1.81 (1.58, 2.08) | 1.81 (1.58, 2.08) | 1.81 (1.57, 2.07) | 1.81 (1.58, 2.08) | 1.81 (1.58, 2.08) | 1.81 (1.58, 2.08) | 1.81 (1.57, 2.08) |
| School governance | Community | Reference | Reference | Reference | Reference | Reference | Reference | Reference | Reference |
|  | Sponsor led academy | 0.59 (0.48, 0.73) | 0.56 (0.45, 0.69) | 0.56 (0.45, 0.69) | 0.57 (0.46, 0.70) | 0.54 (0.44, 0.67) | 0.54 (0.44, 0.68) | 0.56 (0.45, 0.69) | 0.56 (0.45, 0.69) |
|  | Converter led academy | 0.85 (0.74, 0.97) | 0.81 (0.71, 0.94) | 0.82 (0.71, 0.94) | 0.82 (0.71, 0.94) | 0.79 (0.68, 0.91) | 0.79 (0.68, 0.91) | 0.81 (0.71, 0.94) | 0.81 (0.71, 0.94) |
|  | Free school | 1.06 (0.62, 1.81) | 1.04 (0.60, 1.79) | 1.03 (0.60, 1.79) | 1.04 (0.60, 1.80) | 1.03 (0.59, 1.78) | 1.03 (0.60, 1.78) | 1.04 (0.60, 1.79) | 1.04 (0.60, 1.80) |
|  | Voluntary aided | 0.57 (0.49, 0.65) | 0.53 (0.46, 0.61) | 0.53 (0.46, 0.61) | 0.53 (0.46, 0.61) | 0.53 (0.46, 0.61) | 0.53 (0.46, 0.61) | 0.53 (0.46, 0.61) | 0.53 (0.46, 0.61) |
|  | Voluntary controlled | 0.71 (0.60, 0.85) | 0.69 (0.58, 0.82) | 0.69 (0.58, 0.83) | 0.70 (0.58, 0.83) | 0.69 (0.57, 0.82) | 0.69 (0.57, 0.82) | 0.68 (0.57, 0.81) | 0.68 (0.57, 0.81) |
| Missing EYFSP score | Yes vs. no (ref.) |  |  |  |  |  |  |  |  |
| Standardised EYFSP score |  |  |  |  |  |  |  |  |  |
| _cons |  | 0.00 (0.00, 0.01) | 0.00 (0.00, 0.00) | 0.00 (0.00, 0.00) | 0.00 (0.00, 0.00) | 0.00 (0.00, 0.01) | 0.00 (0.00, 0.00) | 0.00 (0.00, 0.00) | 0.00 (0.00, 0.00) |
| *LA-specific associations* | |  |  |  |  |  |  |  |  |
| Pupil population | Linear term |  |  | 1.00 (1.00, 1.00) |  |  |  |  |  |
|  | Quadratic term |  |  | 1.00 (1.00, 1.00) |  |  |  |  |  |
| Special school attendance | Linear term |  |  |  | 2.97 (1.54, 5.73) |  |  |  |  |
|  | Quadratic term |  |  |  | 0.72 (0.49, 1.06) |  |  |  |  |
| Maintained school attendance | Linear term |  |  |  |  | 1.00 (0.99, 1.00) |  |  |  |
| Academy attendance | Linear term |  |  |  |  |  | 1.00 (1.00, 1.01) |  |  |
| FSM eligible | Linear term |  |  |  |  |  |  | 0.96 (0.93, 0.99) |  |
|  | Quadratic term |  |  |  |  |  |  | 1.00 (1.00, 1.00) |  |
| IDACI mode groups | 1 Most deprived 20% |  |  |  |  |  |  |  | 0.81 (0.65, 1.00) |
|  | 2 |  |  |  |  |  |  |  | 0.86 (0.67, 1.10) |
|  | 3 |  |  |  |  |  |  |  | 0.71 (0.54, 0.94) |
|  | 4 |  |  |  |  |  |  |  | 1.19 (0.92, 1.53) |
|  | 5 Least deprived 20% |  |  |  |  |  |  |  | Reference |
| *LA-general effects* |  |  |  |  |  |  |  |  |  |
| LA variance (95% CI) |  |  | 0.20 (0.15, 0.28) | 0.20 (0.14, 0.28) | 0.17 (0.12, 0.24) | 0.20 (0.15, 0.28) | 0.20 (0.15, 0.28) | 0.19 (0.14, 0.27) | 0.18 (0.12, 0.25) |
| PCV* |  |  |  | 3.38% | 17.81% | 0.48% | 0.27% | 5.30% | 14.11% |
| AUC (95% CI) |  | 0.81 (0.80, 0.81) | 0.83 (0.82, 0.83) | 0.83 (0.82, 0.83) | 0.83 (0.82, 0.83) | 0.83 (0.82, 0.83) | 0.83 (0.82, 0.83) | 0.83 (0.82, 0.83) | 0.83 (0.82, 0.83) |
| AUC change* |  |  | 0.02 | 0.00 | 0.00 | 0.00 | 0.00 | 0.00 | 0.00 |
| ICC % (95% CI) |  |  | 5.84 (4.26, 7.97) | 5.66 (4.11, 7.74) | 4.85 (3.45, 6.79) | 5.82 (4.24, 7.94) | 5.83 (4.25, 7.95) | 5.55 (4.00, 7.65) | 5.06 (3.63, 7.02) |
| *Model parameters/goodness of fit* |  |  |  |  |  |  |  |  |  |
| N |  | 27004 | 27004 | 27004 | 27004 | 27004 | 27004 | 27004 | 27004 |
| Log likelihood |  | -8615.4 | -8498.2 | -8496.3 | -8482.8 | -8496.4 | -8496.6 | -8493.7 | -8491.1 |
| Degrees of freedom |  | 44 | 45 | 47 | 47 | 46 | 46 | 47 | 49 |
| AIC |  | 17318.7 | 17086.4 | 17086.7 | 17059.7 | 17084.7 | 17085.2 | 17081.4 | 17080.2 |
| AIC change* |  |  | -232.3 | 0.3 | -26.7 | -1.7 | -1.2 | -5.0 | -6.2 |
| BIC |  | 17679.7 | 17455.6 | 17472.2 | 17445.2 | 17462.1 | 17462.6 | 17466.9 | 17482.2 |
| BIC change* |  |  | -224.1 | 16.6 | -10.4 | 6.5 | 7.0 | 11.3 | 26.6 |
| **(c) EHCP vs. no SEND provision** |  | **Step one (single level** | **Step two (multilevel)** | **Step three (multilevel, + pupil headcount)** | **Step three (multilevel, + special school attendance %)** | **Step three (multilevel, + maintained school attendance %)** | **Step three (multilevel, + academy school attendance %)** | **Step three (multilevel, + FSM eligible %)** | **Step three (multilevel, + IDACI mode)** |
|  |  | **cOR (95% CI)** | **cOR (95% CI)** | **cOR (95% CI)** | **cOR (95% CI)** | **cOR (95% CI)** | **cOR (95% CI)** | **cOR (95% CI)** | **cOR (95% CI)** |
| *Child-specific associations* |  |  |  |  |  |  |  |  |  |
| Year of birth (1 September to 31 August) | 2003/04 | Reference | Reference | Reference | Reference | Reference | Reference | Reference | Reference |
|  | 2004/05 | 1.07 (0.89, 1.30) | 1.06 (0.88, 1.29) | 1.06 (0.88, 1.29) | 1.04 (0.86, 1.27) | 1.06 (0.87, 1.29) | 1.06 (0.87, 1.29) | 1.09 (0.90, 1.32) | 1.07 (0.88, 1.30) |
|  | 2005/06 | 0.93 (0.76, 1.13) | 0.92 (0.76, 1.12) | 0.92 (0.76, 1.13) | 0.92 (0.75, 1.12) | 0.92 (0.75, 1.12) | 0.92 (0.75, 1.12) | 0.96 (0.79, 1.17) | 0.94 (0.77, 1.14) |
|  | 2006/07 | 0.94 (0.77, 1.14) | 0.93 (0.76, 1.13) | 0.93 (0.76, 1.13) | 0.92 (0.75, 1.12) | 0.91 (0.74, 1.12) | 0.91 (0.75, 1.12) | 0.95 (0.78, 1.17) | 0.93 (0.76, 1.14) |
|  | 2007/08 | 0.98 (0.81, 1.19) | 0.97 (0.80, 1.18) | 0.98 (0.80, 1.18) | 0.92 (0.76, 1.11) | 0.95 (0.78, 1.16) | 0.95 (0.78, 1.16) | 0.99 (0.81, 1.20) | 0.99 (0.82, 1.20) |
|  | 2008/09 | 0.87 (0.73, 1.05) | 0.87 (0.72, 1.05) | 0.87 (0.72, 1.05) | 0.80 (0.66, 0.97) | 0.84 (0.69, 1.02) | 0.85 (0.70, 1.03) | 0.86 (0.71, 1.04) | 0.88 (0.73, 1.06) |
|  | 2009/10 | 0.88 (0.73, 1.05) | 0.88 (0.73, 1.05) | 0.88 (0.73, 1.06) | 0.79 (0.66, 0.96) | 0.84 (0.69, 1.03) | 0.85 (0.70, 1.03) | 0.82 (0.68, 0.99) | 0.89 (0.74, 1.07) |
|  | 2010/11 | 0.83 (0.69, 0.99) | 0.82 (0.69, 0.99) | 0.82 (0.68, 0.99) | 0.74 (0.61, 0.89) | 0.79 (0.64, 0.97) | 0.79 (0.64, 0.97) | 0.76 (0.63, 0.92) | 0.84 (0.70, 1.01) |
|  | 2011/12 | 0.95 (0.79, 1.14) | 0.95 (0.79, 1.14) | 0.95 (0.79, 1.14) | 0.82 (0.68, 1.00) | 0.89 (0.72, 1.11) | 0.90 (0.73, 1.11) | 0.88 (0.73, 1.06) | 0.96 (0.80, 1.15) |
|  | 2012/13 | 1.00 (0.84, 1.20) | 1.00 (0.83, 1.19) | 0.99 (0.83, 1.19) | 0.86 (0.71, 1.04) | 0.93 (0.74, 1.16) | 0.94 (0.75, 1.17) | 0.98 (0.82, 1.18) | 1.01 (0.84, 1.21) |
| Maternal age | Linear term | 0.94 (0.89, 0.98) | 0.94 (0.89, 0.98) | 0.94 (0.89, 0.98) | 0.94 (0.89, 0.98) | 0.94 (0.89, 0.98) | 0.94 (0.89, 0.98) | 0.94 (0.89, 0.98) | 0.94 (0.89, 0.98) |
|  | Quadratic term | 1.00 (1.00, 1.00) | 1.00 (1.00, 1.00) | 1.00 (1.00, 1.00) | 1.00 (1.00, 1.00) | 1.00 (1.00, 1.00) | 1.00 (1.00, 1.00) | 1.00 (1.00, 1.00) | 1.00 (1.00, 1.00) |
|  | Missing | 0.59 (0.26, 1.34) | 0.60 (0.26, 1.39) | 0.60 (0.26, 1.39) | 0.60 (0.26, 1.38) | 0.60 (0.26, 1.39) | 0.60 (0.26, 1.39) | 0.59 (0.26, 1.37) | 0.59 (0.26, 1.37) |
| Gender | Male vs. female (ref.) | 2.64 (2.42, 2.88) | 2.65 (2.43, 2.89) | 2.65 (2.43, 2.89) | 2.65 (2.43, 2.89) | 2.65 (2.43, 2.89) | 2.65 (2.43, 2.89) | 2.65 (2.43, 2.89) | 2.65 (2.43, 2.89) |
| Chronic condition (cancer/blood) | Yes vs. no (ref.) | 0.94 (0.75, 1.18) | 0.95 (0.75, 1.20) | 0.95 (0.75, 1.20) | 0.95 (0.75, 1.20) | 0.95 (0.75, 1.20) | 0.95 (0.75, 1.20) | 0.95 (0.75, 1.19) | 0.95 (0.75, 1.19) |
| Chronic condition (cardiovascular) | Yes vs. no (ref.) | 3.59 (3.11, 4.14) | 3.65 (3.15, 4.21) | 3.65 (3.16, 4.22) | 3.65 (3.16, 4.22) | 3.64 (3.15, 4.21) | 3.64 (3.15, 4.21) | 3.65 (3.16, 4.22) | 3.65 (3.16, 4.22) |
| Chronic condition (chronic infection) | Yes vs. no (ref.) | 1.34 (0.73, 2.47) | 1.40 (0.76, 2.57) | 1.40 (0.76, 2.58) | 1.39 (0.75, 2.56) | 1.40 (0.76, 2.58) | 1.40 (0.76, 2.58) | 1.40 (0.76, 2.58) | 1.40 (0.76, 2.58) |
| Chronic condition (mental health/developmental) | Yes vs. no (ref.) | 34.98 (30.06, 40.72) | 36.24 (31.06, 42.28) | 36.22 (31.05, 42.26) | 36.31 (31.12, 42.36) | 36.22 (31.04, 42.25) | 36.22 (31.05, 42.26) | 36.36 (31.16, 42.42) | 36.30 (31.11, 42.35) |
| Chronic condition (metabolic endocrine) | Yes vs. no (ref.) | 1.15 (1.01, 1.31) | 1.14 (1.00, 1.30) | 1.14 (1.00, 1.30) | 1.14 (1.00, 1.30) | 1.14 (1.00, 1.30) | 1.14 (1.00, 1.30) | 1.14 (1.00, 1.30) | 1.14 (1.00, 1.30) |
| Chronic condition (musculoskeletal skin) | Yes vs. no (ref.) | 1.90 (1.57, 2.29) | 1.91 (1.58, 2.32) | 1.91 (1.58, 2.31) | 1.92 (1.59, 2.32) | 1.91 (1.58, 2.31) | 1.91 (1.58, 2.31) | 1.91 (1.58, 2.31) | 1.91 (1.58, 2.31) |
| Chronic condition (neurological) | Yes vs. no (ref.) | 9.37 (8.56, 10.26) | 9.57 (8.73, 10.49) | 9.58 (8.74, 10.50) | 9.56 (8.72, 10.48) | 9.57 (8.73, 10.49) | 9.57 (8.73, 10.49) | 9.58 (8.74, 10.50) | 9.56 (8.73, 10.48) |
| Chronic condition (non-specific) | Yes vs. no (ref.) | 4.71 (4.16, 5.34) | 4.82 (4.24, 5.47) | 4.81 (4.24, 5.46) | 4.80 (4.23, 5.45) | 4.82 (4.24, 5.47) | 4.82 (4.24, 5.46) | 4.82 (4.25, 5.47) | 4.82 (4.24, 5.47) |
| Chronic condition (respiratory) | Yes vs. no (ref.) | 0.99 (0.86, 1.13) | 0.98 (0.85, 1.12) | 0.98 (0.85, 1.12) | 0.97 (0.85, 1.12) | 0.98 (0.85, 1.12) | 0.98 (0.85, 1.12) | 0.98 (0.85, 1.12) | 0.98 (0.85, 1.12) |
| IDACI | 1 Most deprived 20% | 1.28 (1.11, 1.49) | 1.45 (1.24, 1.68) | 1.44 (1.24, 1.68) | 1.42 (1.22, 1.65) | 1.45 (1.24, 1.68) | 1.45 (1.24, 1.68) | 1.52 (1.30, 1.77) | 1.50 (1.28, 1.75) |
|  | 2 | 1.35 (1.17, 1.56) | 1.42 (1.22, 1.64) | 1.42 (1.22, 1.64) | 1.41 (1.21, 1.63) | 1.42 (1.22, 1.64) | 1.42 (1.22, 1.64) | 1.45 (1.25, 1.68) | 1.45 (1.25, 1.68) |
|  | 3 | 1.24 (1.06, 1.43) | 1.28 (1.10, 1.48) | 1.27 (1.10, 1.48) | 1.27 (1.09, 1.48) | 1.27 (1.09, 1.48) | 1.27 (1.09, 1.48) | 1.29 (1.11, 1.50) | 1.29 (1.11, 1.51) |
|  | 4 | 1.20 (1.03, 1.40) | 1.21 (1.04, 1.41) | 1.21 (1.04, 1.42) | 1.21 (1.04, 1.42) | 1.21 (1.04, 1.41) | 1.21 (1.04, 1.41) | 1.22 (1.04, 1.42) | 1.21 (1.04, 1.42) |
|  | 5 Least deprived 20% | Reference | Reference | Reference | Reference | Reference | Reference | Reference | Reference |
|  | Missing | 4.61 (0.49, 43.70) | 5.28 (0.53, 52.35) | 5.26 (0.53, 52.08) | 5.46 (0.56, 53.63) | 5.32 (0.54, 52.83) | 5.31 (0.53, 52.76) | 5.54 (0.56, 54.76) | 5.47 (0.55, 54.02) |
| FSM eligible | Yes vs. no (ref.) | 1.75 (1.59, 1.92) | 1.76 (1.60, 1.94) | 1.76 (1.60, 1.94) | 1.76 (1.60, 1.93) | 1.76 (1.60, 1.94) | 1.76 (1.60, 1.94) | 1.77 (1.61, 1.95) | 1.77 (1.61, 1.94) |
| Racial-ethnic group | Asian | 1.36 (1.17, 1.58) | 1.39 (1.19, 1.62) | 1.39 (1.19, 1.62) | 1.37 (1.18, 1.61) | 1.39 (1.19, 1.62) | 1.39 (1.19, 1.62) | 1.40 (1.20, 1.64) | 1.40 (1.20, 1.64) |
|  | Black | 1.64 (1.39, 1.94) | 1.68 (1.41, 2.01) | 1.68 (1.40, 2.00) | 1.67 (1.40, 1.99) | 1.68 (1.41, 2.01) | 1.68 (1.41, 2.01) | 1.71 (1.44, 2.05) | 1.71 (1.43, 2.04) |
|  | Chinese | 2.07 (1.19, 3.61) | 2.18 (1.26, 3.80) | 2.18 (1.25, 3.80) | 2.18 (1.26, 3.80) | 2.19 (1.26, 3.81) | 2.19 (1.26, 3.81) | 2.20 (1.27, 3.83) | 2.20 (1.27, 3.83) |
|  | Mixed | 1.15 (0.98, 1.35) | 1.15 (0.98, 1.35) | 1.15 (0.98, 1.35) | 1.15 (0.98, 1.35) | 1.15 (0.98, 1.35) | 1.15 (0.98, 1.36) | 1.16 (0.98, 1.36) | 1.16 (0.98, 1.36) |
|  | Other | 1.07 (0.74, 1.53) | 1.02 (0.71, 1.48) | 1.02 (0.71, 1.48) | 1.01 (0.70, 1.46) | 1.03 (0.71, 1.48) | 1.03 (0.71, 1.48) | 1.04 (0.72, 1.50) | 1.05 (0.73, 1.51) |
|  | White | Reference | Reference | Reference | Reference | Reference | Reference | Reference | Reference |
|  | Missing | 1.86 (1.06, 3.25) | 1.84 (1.05, 3.21) | 1.83 (1.05, 3.21) | 1.81 (1.04, 3.17) | 1.83 (1.05, 3.21) | 1.83 (1.05, 3.21) | 1.84 (1.06, 3.22) | 1.84 (1.05, 3.22) |
| EAL | No | Reference | Reference | Reference | Reference | Reference | Reference | Reference | Reference |
|  | Yes | 0.99 (0.86, 1.14) | 0.97 (0.85, 1.12) | 0.97 (0.85, 1.12) | 0.98 (0.85, 1.13) | 0.98 (0.85, 1.12) | 0.98 (0.85, 1.12) | 0.98 (0.85, 1.12) | 0.98 (0.85, 1.13) |
|  | Unclear or missing | 1.50 (0.77, 2.89) | 1.52 (0.78, 2.96) | 1.53 (0.79, 2.96) | 1.53 (0.79, 2.96) | 1.52 (0.78, 2.95) | 1.52 (0.78, 2.95) | 1.53 (0.79, 2.97) | 1.52 (0.78, 2.96) |
| Rate of hospitalisation (pre year 1) |  | 1.01 (1.01, 1.01) | 1.01 (1.01, 1.01) | 1.01 (1.01, 1.01) | 1.01 (1.01, 1.01) | 1.01 (1.01, 1.01) | 1.01 (1.01, 1.01) | 1.01 (1.01, 1.01) | 1.01 (1.01, 1.01) |
| Age at Year One start |  | 0.78 (0.68, 0.89) | 0.78 (0.68, 0.89) | 0.78 (0.68, 0.89) | 0.78 (0.68, 0.90) | 0.78 (0.68, 0.89) | 0.78 (0.68, 0.89) | 0.78 (0.68, 0.89) | 0.78 (0.68, 0.89) |
| School governance | Community | Reference | Reference | Reference | Reference | Reference | Reference | Reference | Reference |
|  | Sponsor led academy | 0.63 (0.51, 0.77) | 0.61 (0.49, 0.76) | 0.61 (0.50, 0.76) | 0.62 (0.50, 0.76) | 0.60 (0.49, 0.75) | 0.60 (0.49, 0.75) | 0.61 (0.49, 0.75) | 0.61 (0.49, 0.75) |
|  | Converter led academy | 0.79 (0.70, 0.91) | 0.77 (0.67, 0.89) | 0.77 (0.67, 0.89) | 0.78 (0.68, 0.89) | 0.76 (0.66, 0.87) | 0.76 (0.66, 0.88) | 0.77 (0.67, 0.88) | 0.77 (0.67, 0.89) |
|  | Free school | 0.84 (0.51, 1.37) | 0.80 (0.49, 1.32) | 0.80 (0.49, 1.31) | 0.80 (0.49, 1.31) | 0.80 (0.49, 1.31) | 0.80 (0.49, 1.31) | 0.81 (0.49, 1.32) | 0.81 (0.49, 1.33) |
|  | Voluntary aided | 0.48 (0.42, 0.55) | 0.47 (0.41, 0.54) | 0.47 (0.41, 0.54) | 0.47 (0.41, 0.54) | 0.47 (0.41, 0.54) | 0.47 (0.41, 0.54) | 0.47 (0.41, 0.54) | 0.47 (0.41, 0.54) |
|  | Voluntary controlled | 0.61 (0.52, 0.73) | 0.58 (0.49, 0.69) | 0.58 (0.49, 0.69) | 0.58 (0.49, 0.70) | 0.58 (0.49, 0.69) | 0.58 (0.49, 0.69) | 0.57 (0.48, 0.68) | 0.57 (0.48, 0.68) |
| Missing EYSFSP score | Yes vs. no (ref.) |  |  |  |  |  |  |  |  |
| Standardised EYSFP score |  |  |  |  |  |  |  |  |  |
| _cons |  | 0.01 (0.01, 0.04) | 0.01 (0.00, 0.04) | 0.00 (0.00, 0.00) | 0.00 (0.00, 0.00) | 0.02 (0.00, 0.05) | 0.01 (0.00, 0.04) | 0.00 (0.00, 0.00) | 0.01 (0.00, 0.04) |
| *LA-specific associations* | |  |  |  |  |  |  |  |  |
| Pupil population | Linear term |  |  | 1.00 (1.00, 1.00) |  |  |  |  |  |
|  | Quadratic term |  |  | 1.00 (1.00, 1.00) |  |  |  |  |  |
| Special school attendance | Linear term |  |  |  | 2.37 (1.26, 4.46) |  |  |  |  |
|  | Quadratic term |  |  |  | 0.80 (0.56, 1.16) |  |  |  |  |
| Maintained school attendance | Linear term |  |  |  |  | 1.00 (0.99, 1.00) |  |  |  |
| Academy attendance | Linear term |  |  |  |  |  | 1.00 (1.00, 1.01) |  |  |
| FSM eligible | Linear term |  |  |  |  |  |  | 0.94 (0.91, 0.97) |  |
|  | Quadratic term |  |  |  |  |  |  | 1.00 (1.00, 1.00) |  |
| IDACI mode groups | 1 Most deprived 20% |  |  |  |  |  |  |  | 0.78 (0.64, 0.94) |
|  | 2 |  |  |  |  |  |  |  | 0.86 (0.69, 1.07) |
|  | 3 |  |  |  |  |  |  |  | 0.69 (0.54, 0.88) |
|  | 4 |  |  |  |  |  |  |  | 1.20 (0.96, 1.51) |
|  | 5 Least deprived 20% |  |  |  |  |  |  |  | Reference |
| *LA-general effects* |  |  |  |  |  |  |  |  |  |
| LA variance (95% CI) |  |  | 0.14 (0.10, 0.20) | 0.14 (0.10, 0.20) | 0.11 (0.08, 0.17) | 0.14 (0.10, 0.20) | 0.14 (0.10, 0.20) | 0.12 (0.08, 0.18) | 0.11 (0.08, 0.17) |
| PCV* |  |  |  | 1.35% | 18.60% | 0.20% | 0.14% | 14.15% | 20.20% |
| AUC (95% CI) |  | 0.87 (0.86, 0.87) | 0.88 (0.87, 0.89) | 0.88 (0.87, 0.89) | 0.88 (0.87, 0.89) | 0.88 (0.87, 0.89) | 0.88 (0.87, 0.89) | 0.88 (0.87, 0.89) | 0.88 (0.87, 0.89) |
| AUC change* |  |  | 0.01 | 0.00 | 0.00 | 0.00 | 0.00 | 0.00 | 0.00 |
| ICC % (95% CI) |  |  | 4.07 (2.86, 5.77) | 4.02 (2.81, 5.71) | 3.34 (2.26, 4.91) | 4.06 (2.85, 5.76) | 4.07 (2.85, 5.76) | 3.52 (2.40, 5.12) | 3.28 (2.23, 4.78) |
| *Model parameters/goodness of fit* |  |  |  |  |  |  |  |  |  |
| N |  | 115782 | 115782 | 115782 | 115782 | 115782 | 115782 | 115782 | 115782 |
| Log likelihood |  | -10984.6 | -10908.9 | -10908.4 | -10895.1 | -10908.4 | -10908.5 | -10899.8 | -10897.9 |
| Degrees of freedom |  | 44 | 45 | 47 | 47 | 46 | 46 | 47 | 49 |
| AIC |  | 22057.3 | 21907.8 | 21910.8 | 21884.2 | 21908.7 | 21909.0 | 21893.6 | 21893.9 |
| AIC change* |  |  | -149.5 | 3.0 | -23.6 | 0.9 | 1.2 | -14.2 | -13.9 |
| BIC |  | 22482.3 | 22342.4 | 22364.8 | 22338.2 | 22353.1 | 22353.3 | 22347.6 | 22367.2 |
| BIC change* |  |  | -139.9 | 22.4 | -4.2 | 10.7 | 10.9 | 5.2 | 24.8 |

AIC= Akaike information criterion; AUC = area under the receiving operator characteristic (ROC) curve; BIC = and Bayesian information criterion; CI = confidence interval; cOR = condition odds ratio; EAL = English as an additional language; EHCP = education, health and care plan; EYFSP = Early years foundation stage profile; FSM = free school meals; IDACI = Income deprivation affecting children index; IQR = interquartile range; LA = local authority; PCV=percentage change in variance; SEND=special educational needs and disability; *change in relation to the previous step (i.e. step 1 for step 2 model, step 2 model for step 3 models); Positive values for AUC change and PCV indicate increases in the discimination and variance explained compared with model from the previous step. Negative AIC/BIC change values indicates better model fit.

**Table S5.** Single- and multi-level logistic regression analysis of (a) SEND support vs. no SEND provision, (b) EHCP vs. SEND support and (c) EHCP vs. no SEND provision: children born early term

| **(a) SEND support vs. no SEND provision** |  | **Step one (single level)** | **Step two (multilevel)** | **Step three (multilevel, + pupil headcount)** | **Step three (multilevel, + special school attendance %)** | **Step three (multilevel, + maintained school attendance %)** | **Step three (multilevel, + academy school attendance %)** | **Step three (multilevel, + FSM eligible %)** | **Step three (multilevel, + IDACI mode)** |
| --- | --- | --- | --- | --- | --- | --- | --- | --- | --- |
|  |  | cOR (95% CI) | cOR (95% CI) | cOR (95% CI) | cOR (95% CI) | cOR (95% CI) | cOR (95% CI) | cOR (95% CI) | cOR (95% CI) |
| Child-specific associations |  |  |  |  |  |  |  |  |  |
| Year of birth (1 September to 31 August) | 2003/04 | Reference | Reference | Reference | Reference | Reference | Reference | Reference | Reference |
|  | 2004/05 | 0.92 (0.85, 0.99) | 0.90 (0.83, 0.98) | 0.90 (0.83, 0.98) | 0.90 (0.83, 0.97) | 0.90 (0.83, 0.98) | 0.90 (0.83, 0.98) | 0.91 (0.84, 0.98) | 0.90 (0.83, 0.98) |
|  | 2005/06 | 0.86 (0.79, 0.93) | 0.85 (0.79, 0.92) | 0.85 (0.79, 0.93) | 0.85 (0.79, 0.92) | 0.86 (0.79, 0.93) | 0.86 (0.79, 0.93) | 0.86 (0.79, 0.93) | 0.85 (0.79, 0.92) |
|  | 2006/07 | 0.77 (0.71, 0.84) | 0.78 (0.71, 0.84) | 0.78 (0.72, 0.85) | 0.78 (0.71, 0.84) | 0.79 (0.72, 0.86) | 0.79 (0.73, 0.86) | 0.78 (0.72, 0.85) | 0.78 (0.71, 0.84) |
|  | 2007/08 | 0.66 (0.61, 0.71) | 0.66 (0.61, 0.72) | 0.66 (0.61, 0.72) | 0.65 (0.60, 0.71) | 0.68 (0.62, 0.74) | 0.68 (0.62, 0.74) | 0.66 (0.61, 0.72) | 0.66 (0.61, 0.72) |
|  | 2008/09 | 0.55 (0.51, 0.59) | 0.55 (0.51, 0.59) | 0.55 (0.51, 0.60) | 0.54 (0.50, 0.59) | 0.57 (0.52, 0.62) | 0.57 (0.52, 0.62) | 0.55 (0.50, 0.59) | 0.55 (0.51, 0.60) |
|  | 2009/10 | 0.52 (0.48, 0.56) | 0.52 (0.48, 0.56) | 0.52 (0.48, 0.57) | 0.51 (0.47, 0.55) | 0.54 (0.49, 0.59) | 0.54 (0.50, 0.59) | 0.51 (0.47, 0.55) | 0.52 (0.48, 0.56) |
|  | 2010/11 | 0.51 (0.48, 0.55) | 0.52 (0.48, 0.56) | 0.52 (0.48, 0.57) | 0.51 (0.47, 0.55) | 0.54 (0.49, 0.59) | 0.54 (0.50, 0.59) | 0.50 (0.46, 0.55) | 0.52 (0.48, 0.56) |
|  | 2011/12 | 0.52 (0.48, 0.57) | 0.52 (0.48, 0.56) | 0.53 (0.49, 0.57) | 0.51 (0.47, 0.55) | 0.55 (0.50, 0.61) | 0.56 (0.51, 0.61) | 0.51 (0.47, 0.55) | 0.52 (0.48, 0.56) |
|  | 2012/13 | 0.52 (0.48, 0.56) | 0.52 (0.48, 0.56) | 0.53 (0.49, 0.57) | 0.51 (0.47, 0.55) | 0.56 (0.50, 0.62) | 0.56 (0.51, 0.62) | 0.51 (0.48, 0.56) | 0.52 (0.48, 0.56) |
| Maternal age | Linear term | 0.98 (0.96, 1.01) | 0.98 (0.96, 1.01) | 0.98 (0.96, 1.01) | 0.98 (0.96, 1.01) | 0.98 (0.96, 1.01) | 0.98 (0.96, 1.01) | 0.98 (0.96, 1.01) | 0.98 (0.96, 1.01) |
|  | Quadratic term | 1.00 (1.00, 1.00) | 1.00 (1.00, 1.00) | 1.00 (1.00, 1.00) | 1.00 (1.00, 1.00) | 1.00 (1.00, 1.00) | 1.00 (1.00, 1.00) | 1.00 (1.00, 1.00) | 1.00 (1.00, 1.00) |
|  | Missing | 0.90 (0.61, 1.33) | 0.93 (0.63, 1.37) | 0.93 (0.63, 1.36) | 0.92 (0.63, 1.36) | 0.93 (0.63, 1.37) | 0.93 (0.63, 1.37) | 0.93 (0.63, 1.36) | 0.93 (0.63, 1.36) |
| Gender | Male vs. female (ref.) | 1.72 (1.65, 1.78) | 1.71 (1.65, 1.78) | 1.71 (1.65, 1.78) | 1.71 (1.65, 1.78) | 1.71 (1.65, 1.78) | 1.71 (1.65, 1.78) | 1.71 (1.65, 1.78) | 1.71 (1.65, 1.78) |
| Chronic condition (cancer/blood) | Yes vs. no (ref.) | 1.27 (1.09, 1.49) | 1.25 (1.07, 1.47) | 1.26 (1.07, 1.47) | 1.26 (1.07, 1.47) | 1.26 (1.07, 1.47) | 1.26 (1.07, 1.47) | 1.25 (1.07, 1.47) | 1.25 (1.07, 1.47) |
| Chronic condition (cardiovascular) | Yes vs. no (ref.) | 1.31 (1.13, 1.51) | 1.31 (1.14, 1.52) | 1.32 (1.14, 1.52) | 1.31 (1.13, 1.52) | 1.32 (1.14, 1.52) | 1.32 (1.14, 1.53) | 1.31 (1.13, 1.52) | 1.31 (1.13, 1.52) |
| Chronic condition (chronic infection) | Yes vs. no (ref.) | 1.14 (0.73, 1.78) | 1.16 (0.74, 1.81) | 1.16 (0.74, 1.81) | 1.16 (0.74, 1.81) | 1.16 (0.74, 1.82) | 1.16 (0.74, 1.82) | 1.16 (0.74, 1.81) | 1.16 (0.74, 1.82) |
| Chronic condition (mental health/developmental) | Yes vs. no (ref.) | 3.42 (2.88, 4.05) | 3.45 (2.91, 4.10) | 3.45 (2.91, 4.09) | 3.45 (2.91, 4.10) | 3.45 (2.91, 4.10) | 3.45 (2.91, 4.10) | 3.45 (2.91, 4.10) | 3.45 (2.91, 4.09) |
| Chronic condition (metabolic endocrine) | Yes vs. no (ref.) | 1.04 (0.96, 1.12) | 1.04 (0.96, 1.12) | 1.04 (0.96, 1.12) | 1.04 (0.96, 1.12) | 1.04 (0.96, 1.12) | 1.04 (0.96, 1.12) | 1.04 (0.96, 1.12) | 1.04 (0.96, 1.12) |
| Chronic condition (musculoskeletal skin) | Yes vs. no (ref.) | 1.87 (1.63, 2.14) | 1.89 (1.64, 2.17) | 1.89 (1.64, 2.17) | 1.89 (1.64, 2.17) | 1.89 (1.65, 2.17) | 1.89 (1.65, 2.17) | 1.89 (1.64, 2.17) | 1.89 (1.64, 2.17) |
| Chronic condition (neurological) | Yes vs. no (ref.) | 1.95 (1.81, 2.10) | 1.96 (1.82, 2.12) | 1.96 (1.82, 2.12) | 1.96 (1.82, 2.12) | 1.96 (1.82, 2.12) | 1.96 (1.82, 2.12) | 1.96 (1.82, 2.11) | 1.96 (1.82, 2.11) |
| Chronic condition (non-specific) | Yes vs. no (ref.) | 1.42 (1.26, 1.60) | 1.43 (1.27, 1.61) | 1.43 (1.27, 1.61) | 1.43 (1.27, 1.61) | 1.43 (1.27, 1.61) | 1.43 (1.27, 1.61) | 1.43 (1.27, 1.61) | 1.43 (1.27, 1.61) |
| Chronic condition (respiratory) | Yes vs. no (ref.) | 1.11 (1.02, 1.20) | 1.11 (1.03, 1.20) | 1.11 (1.03, 1.20) | 1.11 (1.03, 1.20) | 1.11 (1.03, 1.20) | 1.11 (1.03, 1.20) | 1.11 (1.03, 1.20) | 1.11 (1.03, 1.20) |
| IDACI | 1 Most deprived 20% | 1.35 (1.26, 1.44) | 1.37 (1.27, 1.47) | 1.37 (1.27, 1.47) | 1.37 (1.27, 1.46) | 1.37 (1.27, 1.47) | 1.37 (1.27, 1.47) | 1.38 (1.28, 1.48) | 1.38 (1.28, 1.48) |
|  | 2 | 1.29 (1.21, 1.38) | 1.31 (1.22, 1.40) | 1.30 (1.22, 1.40) | 1.30 (1.22, 1.40) | 1.31 (1.22, 1.40) | 1.31 (1.22, 1.40) | 1.31 (1.22, 1.40) | 1.31 (1.22, 1.40) |
|  | 3 | 1.22 (1.14, 1.30) | 1.21 (1.13, 1.30) | 1.21 (1.13, 1.30) | 1.21 (1.13, 1.30) | 1.21 (1.13, 1.30) | 1.21 (1.13, 1.30) | 1.21 (1.13, 1.30) | 1.21 (1.13, 1.30) |
|  | 4 | 1.10 (1.03, 1.18) | 1.10 (1.02, 1.18) | 1.10 (1.02, 1.18) | 1.10 (1.02, 1.18) | 1.10 (1.02, 1.18) | 1.10 (1.02, 1.18) | 1.10 (1.02, 1.18) | 1.10 (1.02, 1.18) |
|  | 5 Least deprived 20% | Reference | Reference | Reference | Reference | Reference | Reference | Reference | Reference |
|  | Missing | 0.27 (0.01, 7.06) | 0.27 (0.01, 7.89) | 0.27 (0.01, 7.91) | 0.26 (0.01, 7.99) | 0.27 (0.01, 7.86) | 0.27 (0.01, 7.86) | 0.27 (0.01, 7.94) | 0.27 (0.01, 7.94) |
| FSM eligible | Yes vs. no (ref.) | 1.36 (1.31, 1.42) | 1.36 (1.31, 1.42) | 1.36 (1.31, 1.42) | 1.36 (1.31, 1.42) | 1.36 (1.31, 1.42) | 1.36 (1.31, 1.42) | 1.36 (1.31, 1.42) | 1.36 (1.31, 1.42) |
| Racial-ethnic group | Asian | 0.81 (0.76, 0.87) | 0.80 (0.74, 0.85) | 0.80 (0.74, 0.85) | 0.80 (0.74, 0.85) | 0.80 (0.74, 0.85) | 0.80 (0.74, 0.85) | 0.80 (0.75, 0.86) | 0.80 (0.75, 0.86) |
|  | Black | 1.07 (0.99, 1.15) | 0.96 (0.89, 1.04) | 0.96 (0.89, 1.04) | 0.96 (0.89, 1.04) | 0.96 (0.89, 1.04) | 0.96 (0.89, 1.04) | 0.97 (0.89, 1.05) | 0.97 (0.89, 1.05) |
|  | Chinese | 0.69 (0.51, 0.92) | 0.66 (0.49, 0.88) | 0.66 (0.49, 0.88) | 0.66 (0.49, 0.88) | 0.66 (0.49, 0.88) | 0.66 (0.49, 0.88) | 0.66 (0.49, 0.88) | 0.66 (0.49, 0.88) |
|  | Mixed | 0.95 (0.88, 1.02) | 0.90 (0.83, 0.97) | 0.90 (0.83, 0.96) | 0.90 (0.83, 0.96) | 0.90 (0.83, 0.97) | 0.90 (0.83, 0.97) | 0.90 (0.83, 0.97) | 0.90 (0.83, 0.97) |
|  | Other | 0.85 (0.73, 0.97) | 0.77 (0.67, 0.89) | 0.77 (0.67, 0.89) | 0.77 (0.67, 0.89) | 0.77 (0.67, 0.89) | 0.77 (0.67, 0.89) | 0.77 (0.67, 0.89) | 0.77 (0.67, 0.89) |
|  | White | Reference | Reference | Reference | Reference | Reference | Reference | Reference | Reference |
|  | Missing | 0.84 (0.59, 1.18) | 0.76 (0.54, 1.08) | 0.76 (0.54, 1.08) | 0.77 (0.54, 1.08) | 0.76 (0.54, 1.08) | 0.76 (0.54, 1.08) | 0.76 (0.54, 1.08) | 0.76 (0.54, 1.08) |
| EAL | No | Reference | Reference | Reference | Reference | Reference | Reference | Reference | Reference |
|  | Yes | 0.74 (0.70, 0.79) | 0.71 (0.67, 0.76) | 0.71 (0.67, 0.76) | 0.71 (0.67, 0.76) | 0.71 (0.67, 0.76) | 0.71 (0.67, 0.76) | 0.71 (0.67, 0.76) | 0.71 (0.67, 0.76) |
|  | Unclear or missing | 0.92 (0.67, 1.25) | 0.88 (0.65, 1.20) | 0.88 (0.65, 1.21) | 0.88 (0.65, 1.20) | 0.88 (0.65, 1.21) | 0.88 (0.65, 1.21) | 0.88 (0.65, 1.21) | 0.88 (0.65, 1.21) |
| Rate of hospitalisation (pre year 1) |  | 1.01 (1.00, 1.01) | 1.01 (1.00, 1.01) | 1.01 (1.00, 1.01) | 1.01 (1.00, 1.01) | 1.01 (1.00, 1.01) | 1.01 (1.00, 1.01) | 1.01 (1.00, 1.01) | 1.01 (1.00, 1.01) |
| Age at Year One start |  | 1.20 (1.13, 1.28) | 1.23 (1.15, 1.30) | 1.23 (1.15, 1.30) | 1.23 (1.15, 1.30) | 1.23 (1.15, 1.30) | 1.23 (1.15, 1.30) | 1.23 (1.15, 1.30) | 1.23 (1.15, 1.30) |
| School governance | Community | Reference | Reference | Reference | Reference | Reference | Reference | Reference | Reference |
|  | Sponsor led academy | 0.92 (0.85, 1.00) | 0.93 (0.85, 1.01) | 0.93 (0.86, 1.01) | 0.93 (0.85, 1.01) | 0.94 (0.87, 1.02) | 0.94 (0.87, 1.02) | 0.93 (0.85, 1.01) | 0.93 (0.85, 1.01) |
|  | Converter led academy | 0.94 (0.88, 0.99) | 0.96 (0.90, 1.02) | 0.96 (0.90, 1.02) | 0.96 (0.90, 1.02) | 0.98 (0.91, 1.04) | 0.98 (0.92, 1.04) | 0.96 (0.90, 1.02) | 0.96 (0.90, 1.02) |
|  | Free school | 1.03 (0.80, 1.32) | 1.00 (0.78, 1.29) | 1.00 (0.78, 1.29) | 1.01 (0.78, 1.29) | 1.01 (0.78, 1.30) | 1.01 (0.78, 1.29) | 1.00 (0.78, 1.29) | 1.01 (0.78, 1.29) |
|  | Voluntary aided | 0.91 (0.87, 0.96) | 0.92 (0.88, 0.97) | 0.92 (0.88, 0.97) | 0.92 (0.88, 0.97) | 0.92 (0.88, 0.97) | 0.92 (0.88, 0.97) | 0.93 (0.88, 0.97) | 0.93 (0.88, 0.97) |
|  | Voluntary controlled | 0.87 (0.82, 0.93) | 0.88 (0.82, 0.94) | 0.88 (0.82, 0.94) | 0.88 (0.82, 0.94) | 0.88 (0.82, 0.94) | 0.88 (0.82, 0.94) | 0.88 (0.82, 0.94) | 0.88 (0.82, 0.94) |
| Missing EYSFSP score | Yes vs. no (ref.) | 2.67 (2.34, 3.06) | 2.68 (2.34, 3.08) | 2.68 (2.34, 3.08) | 2.68 (2.34, 3.08) | 2.69 (2.34, 3.08) | 2.69 (2.34, 3.08) | 2.68 (2.34, 3.08) | 2.69 (2.34, 3.08) |
| Standardised EYSFP score |  | 0.24 (0.23, 0.24) | 0.23 (0.23, 0.24) | 0.23 (0.23, 0.24) | 0.23 (0.23, 0.24) | 0.23 (0.23, 0.24) | 0.23 (0.23, 0.24) | 0.23 (0.23, 0.24) | 0.23 (0.23, 0.24) |
| _cons |  | 0.02 (0.01, 0.04) | 0.02 (0.01, 0.03) | 0.02 (0.01, 0.03) | 0.02 (0.01, 0.03) | 0.02 (0.01, 0.03) | 0.02 (0.01, 0.03) | 0.02 (0.01, 0.04) | 0.02 (0.01, 0.03) |
| LA-specific associations | |  |  |  |  |  |  |  |  |
| Pupil population | Linear term |  |  | 1.00 (1.00, 1.00) |  |  |  |  |  |
|  | Quadratic term |  |  | 1.00 (1.00, 1.00) |  |  |  |  |  |
| Special school attendance | Linear term |  |  |  | 1.23 (0.92, 1.66) |  |  |  |  |
|  | Quadratic term |  |  |  | 0.92 (0.77, 1.10) |  |  |  |  |
| Maintained school attendance | Linear term |  |  |  |  | 1.00 (1.00, 1.00) |  |  |  |
| Academy attendance | Linear term |  |  |  |  |  | 1.00 (1.00, 1.00) |  |  |
| FSM eligible | Linear term |  |  |  |  |  |  | 0.99 (0.97, 1.00) |  |
|  | Quadratic term |  |  |  |  |  |  | 1.00 (1.00, 1.00) |  |
| IDACI mode groups | 1 Most deprived 20% |  |  |  |  |  |  |  | 0.90 (0.80, 1.01) |
|  | 2 |  |  |  |  |  |  |  | 0.94 (0.83, 1.06) |
|  | 3 |  |  |  |  |  |  |  | 1.03 (0.89, 1.18) |
|  | 4 |  |  |  |  |  |  |  | 0.96 (0.84, 1.10) |
|  | 5 Least deprived 20% |  |  |  |  |  |  |  | Reference |
| LA-general effects |  |  |  |  |  |  |  |  |  |
| LA variance (95% CI) |  |  | 0.07 (0.06, 0.10) | 0.08 (0.06, 0.10) | 0.07 (0.05, 0.09) | 0.07 (0.05, 0.10) | 0.07 (0.05, 0.10) | 0.07 (0.05, 0.09) | 0.07 (0.05, 0.10) |
| PCV* |  |  |  | -2.91% | 1.84% | 1.18% | 1.57% | 2.60% | 1.37% |
| AUC (95% CI) |  | 0.85 (0.85, 0.85) | 0.85 (0.85, 0.86) | 0.85 (0.85, 0.86) | 0.85 (0.85, 0.86) | 0.85 (0.85, 0.86) | 0.85 (0.85, 0.86) | 0.85 (0.85, 0.86) | 0.85 (0.85, 0.86) |
| AUC change* |  |  | 0.004 | 0.000 | 0.000 | 0.000 | 0.000 | 0.000 | 0.000 |
| ICC % (95% CI) |  |  | 2.17 (1.65, 2.85) | 2.23 (1.70, 2.94) | 2.13 (1.62, 2.80) | 2.15 (1.63, 2.82) | 2.14 (1.63, 2.81) | 2.12 (1.61, 2.78) | 2.14 (1.63, 2.82) |
| Model parameters/goodness of fit |  |  |  |  |  |  |  |  |  |
| N |  | 143017 | 143017 | 143017 | 143017 | 143017 | 143017 | 143017 | 143017 |
| Log likelihood |  | -43549.3 | -43217.9 | -43216.8 | -43216.3 | -43215.7 | -43215.3 | -43216.2 | -43215.0 |
| Degrees of freedom |  | 46 | 47 | 49 | 49 | 48 | 48 | 49 | 51 |
| AIC |  | 87190.5 | 86529.8 | 86531.5 | 86530.6 | 86527.4 | 86526.6 | 86530.5 | 86532.0 |
| AIC change* |  |  | -660.7 | 1.7 | 0.8 | -2.4 | -3.2 | 0.7 | 2.2 |
| BIC |  | 87644.6 | 86993.7 | 87015.2 | 87014.3 | 87001.2 | 87000.4 | 87014.2 | 87035.4 |
| BIC change* |  |  | -650.9 | 21.5 | 20.6 | 7.5 | 6.7 | 20.5 | 41.7 |
| **(b) EHCP vs. SEND support** |  | **Step one (single level** | **Step two (multilevel)** | **Step three (multilevel, + pupil headcount)** | **Step three (multilevel, + special school attendance %)** | **Step three (multilevel, + maintained school attendance %)** | **Step three (multilevel, + academy school attendance %)** | **Step three (multilevel, + FSM eligible %)** | **Step three (multilevel, + IDACI mode)** |
|  |  | cOR (95% CI) | cOR (95% CI) | cOR (95% CI) | cOR (95% CI) | cOR (95% CI) | cOR (95% CI) | cOR (95% CI) | cOR (95% CI) |
| Child-specific associations |  |  |  |  |  |  |  |  |  |
| Year of birth (1 September to 31 August) | 2003/04 | Reference | Reference | Reference | Reference | Reference | Reference | Reference | Reference |
|  | 2004/05 | 1.42 (1.14, 1.76) | 1.43 (1.15, 1.78) | 1.44 (1.16, 1.78) | 1.41 (1.13, 1.75) | 1.43 (1.15, 1.78) | 1.43 (1.15, 1.78) | 1.46 (1.18, 1.82) | 1.43 (1.15, 1.78) |
|  | 2005/06 | 1.19 (0.95, 1.49) | 1.20 (0.96, 1.51) | 1.21 (0.96, 1.52) | 1.20 (0.95, 1.50) | 1.21 (0.96, 1.52) | 1.21 (0.96, 1.52) | 1.24 (0.99, 1.56) | 1.21 (0.96, 1.52) |
|  | 2006/07 | 1.37 (1.10, 1.72) | 1.38 (1.10, 1.74) | 1.38 (1.10, 1.74) | 1.38 (1.10, 1.73) | 1.39 (1.11, 1.75) | 1.40 (1.11, 1.76) | 1.40 (1.12, 1.76) | 1.38 (1.10, 1.74) |
|  | 2007/08 | 1.71 (1.38, 2.11) | 1.72 (1.39, 2.13) | 1.72 (1.39, 2.14) | 1.62 (1.30, 2.01) | 1.74 (1.40, 2.17) | 1.75 (1.40, 2.18) | 1.70 (1.37, 2.11) | 1.74 (1.40, 2.15) |
|  | 2008/09 | 1.65 (1.34, 2.03) | 1.66 (1.35, 2.05) | 1.67 (1.35, 2.06) | 1.52 (1.23, 1.88) | 1.69 (1.36, 2.11) | 1.70 (1.37, 2.12) | 1.61 (1.30, 1.99) | 1.67 (1.35, 2.07) |
|  | 2009/10 | 1.57 (1.27, 1.92) | 1.58 (1.28, 1.95) | 1.59 (1.28, 1.96) | 1.42 (1.15, 1.76) | 1.62 (1.29, 2.02) | 1.63 (1.30, 2.03) | 1.46 (1.18, 1.81) | 1.59 (1.29, 1.97) |
|  | 2010/11 | 1.76 (1.44, 2.16) | 1.75 (1.42, 2.15) | 1.75 (1.42, 2.16) | 1.55 (1.26, 1.92) | 1.80 (1.43, 2.26) | 1.81 (1.44, 2.28) | 1.60 (1.29, 1.98) | 1.76 (1.43, 2.17) |
|  | 2011/12 | 2.00 (1.63, 2.44) | 2.04 (1.67, 2.51) | 2.04 (1.66, 2.51) | 1.76 (1.43, 2.18) | 2.11 (1.66, 2.67) | 2.13 (1.69, 2.69) | 1.86 (1.50, 2.29) | 2.06 (1.68, 2.52) |
|  | 2012/13 | 1.95 (1.60, 2.39) | 1.99 (1.62, 2.45) | 1.99 (1.62, 2.45) | 1.70 (1.38, 2.11) | 2.07 (1.61, 2.64) | 2.09 (1.64, 2.67) | 1.91 (1.55, 2.35) | 2.01 (1.64, 2.47) |
| Maternal age | Linear term | 1.03 (0.97, 1.08) | 1.03 (0.97, 1.09) | 1.03 (0.97, 1.09) | 1.03 (0.97, 1.09) | 1.03 (0.97, 1.09) | 1.03 (0.97, 1.09) | 1.03 (0.97, 1.09) | 1.03 (0.97, 1.08) |
|  | Quadratic term | 1.00 (1.00, 1.00) | 1.00 (1.00, 1.00) | 1.00 (1.00, 1.00) | 1.00 (1.00, 1.00) | 1.00 (1.00, 1.00) | 1.00 (1.00, 1.00) | 1.00 (1.00, 1.00) | 1.00 (1.00, 1.00) |
|  | Missing | 2.50 (0.98, 6.40) | 2.48 (0.95, 6.45) | 2.49 (0.96, 6.48) | 2.60 (1.00, 6.76) | 2.49 (0.96, 6.46) | 2.49 (0.96, 6.47) | 2.46 (0.95, 6.41) | 2.43 (0.94, 6.31) |
| Gender | Male vs. female (ref.) | 1.34 (1.22, 1.47) | 1.34 (1.22, 1.48) | 1.34 (1.22, 1.48) | 1.34 (1.22, 1.48) | 1.34 (1.22, 1.48) | 1.34 (1.22, 1.48) | 1.34 (1.22, 1.48) | 1.34 (1.22, 1.48) |
| Chronic condition (cancer/blood) | Yes vs. no (ref.) | 0.74 (0.57, 0.95) | 0.73 (0.56, 0.94) | 0.73 (0.56, 0.94) | 0.73 (0.56, 0.95) | 0.73 (0.56, 0.94) | 0.73 (0.56, 0.94) | 0.72 (0.56, 0.94) | 0.73 (0.56, 0.94) |
| Chronic condition (cardiovascular) | Yes vs. no (ref.) | 2.27 (1.89, 2.72) | 2.32 (1.93, 2.80) | 2.33 (1.93, 2.80) | 2.33 (1.93, 2.81) | 2.32 (1.93, 2.80) | 2.33 (1.93, 2.80) | 2.32 (1.92, 2.79) | 2.31 (1.92, 2.79) |
| Chronic condition (chronic infection) | Yes vs. no (ref.) | 0.80 (0.38, 1.72) | 0.81 (0.37, 1.78) | 0.80 (0.36, 1.77) | 0.80 (0.37, 1.76) | 0.81 (0.37, 1.79) | 0.81 (0.37, 1.79) | 0.80 (0.36, 1.77) | 0.81 (0.37, 1.78) |
| Chronic condition (mental health/developmental) | Yes vs. no (ref.) | 5.88 (5.13, 6.73) | 6.09 (5.30, 7.00) | 6.10 (5.30, 7.01) | 6.10 (5.31, 7.01) | 6.10 (5.31, 7.01) | 6.10 (5.31, 7.01) | 6.08 (5.29, 6.99) | 6.08 (5.29, 6.99) |
| Chronic condition (metabolic endocrine) | Yes vs. no (ref.) | 1.18 (1.02, 1.37) | 1.17 (1.01, 1.36) | 1.17 (1.01, 1.36) | 1.17 (1.01, 1.36) | 1.17 (1.01, 1.36) | 1.17 (1.01, 1.36) | 1.17 (1.01, 1.36) | 1.17 (1.01, 1.36) |
| Chronic condition (musculoskeletal skin) | Yes vs. no (ref.) | 1.27 (1.04, 1.55) | 1.29 (1.06, 1.58) | 1.29 (1.06, 1.58) | 1.29 (1.06, 1.58) | 1.29 (1.06, 1.58) | 1.29 (1.06, 1.58) | 1.30 (1.06, 1.59) | 1.29 (1.05, 1.58) |
| Chronic condition (neurological) | Yes vs. no (ref.) | 3.32 (2.98, 3.70) | 3.41 (3.05, 3.80) | 3.41 (3.05, 3.80) | 3.41 (3.05, 3.80) | 3.41 (3.05, 3.80) | 3.41 (3.05, 3.80) | 3.41 (3.06, 3.81) | 3.41 (3.06, 3.81) |
| Chronic condition (non-specific) | Yes vs. no (ref.) | 2.65 (2.30, 3.07) | 2.77 (2.39, 3.21) | 2.77 (2.39, 3.21) | 2.77 (2.39, 3.21) | 2.77 (2.39, 3.21) | 2.77 (2.39, 3.21) | 2.78 (2.40, 3.22) | 2.78 (2.40, 3.22) |
| Chronic condition (respiratory) | Yes vs. no (ref.) | 0.80 (0.69, 0.93) | 0.82 (0.70, 0.95) | 0.82 (0.70, 0.95) | 0.82 (0.71, 0.96) | 0.82 (0.70, 0.95) | 0.82 (0.70, 0.95) | 0.82 (0.70, 0.95) | 0.82 (0.70, 0.95) |
| IDACI | 1 Most deprived 20% | 0.63 (0.53, 0.73) | 0.67 (0.57, 0.79) | 0.67 (0.56, 0.79) | 0.66 (0.56, 0.78) | 0.67 (0.57, 0.79) | 0.67 (0.57, 0.79) | 0.70 (0.59, 0.83) | 0.70 (0.59, 0.83) |
|  | 2 | 0.75 (0.64, 0.88) | 0.76 (0.65, 0.90) | 0.76 (0.65, 0.90) | 0.76 (0.64, 0.89) | 0.76 (0.65, 0.90) | 0.76 (0.65, 0.90) | 0.78 (0.66, 0.91) | 0.78 (0.66, 0.92) |
|  | 3 | 0.89 (0.76, 1.05) | 0.90 (0.76, 1.06) | 0.89 (0.76, 1.06) | 0.90 (0.76, 1.06) | 0.90 (0.76, 1.06) | 0.90 (0.76, 1.06) | 0.90 (0.76, 1.07) | 0.91 (0.77, 1.08) |
|  | 4 | 0.92 (0.78, 1.09) | 0.90 (0.76, 1.08) | 0.90 (0.76, 1.08) | 0.91 (0.76, 1.08) | 0.90 (0.76, 1.08) | 0.90 (0.76, 1.08) | 0.91 (0.76, 1.08) | 0.91 (0.76, 1.08) |
|  | 5 Least deprived 20% | Reference | Reference | Reference | Reference | Reference | Reference | Reference | Reference |
|  | Missing | 3.74 (0.19, 72.95) | 4.24 (0.19, 95.17) | 4.12 (0.19, 91.43) | 4.17 (0.15, 114.10) | 4.26 (0.19, 95.32) | 4.27 (0.19, 95.35) | 4.35 (0.21, 90.91) | 4.10 (0.19, 86.52) |
| FSM eligible | Yes vs. no (ref.) | 0.92 (0.84, 1.02) | 0.91 (0.82, 1.01) | 0.91 (0.82, 1.01) | 0.91 (0.82, 1.01) | 0.91 (0.82, 1.01) | 0.91 (0.82, 1.01) | 0.92 (0.83, 1.02) | 0.91 (0.83, 1.01) |
| Racial-ethnic group | Asian | 1.12 (0.95, 1.32) | 1.16 (0.98, 1.37) | 1.16 (0.98, 1.37) | 1.15 (0.97, 1.36) | 1.16 (0.97, 1.37) | 1.16 (0.97, 1.37) | 1.18 (0.99, 1.40) | 1.17 (0.98, 1.38) |
|  | Black | 1.63 (1.37, 1.94) | 1.68 (1.39, 2.02) | 1.67 (1.39, 2.01) | 1.66 (1.38, 2.00) | 1.68 (1.39, 2.02) | 1.68 (1.39, 2.02) | 1.72 (1.43, 2.08) | 1.71 (1.42, 2.05) |
|  | Chinese | 1.38 (0.70, 2.70) | 1.44 (0.73, 2.84) | 1.44 (0.73, 2.84) | 1.45 (0.74, 2.86) | 1.44 (0.73, 2.84) | 1.44 (0.73, 2.84) | 1.44 (0.73, 2.85) | 1.44 (0.73, 2.85) |
|  | Mixed | 1.13 (0.94, 1.35) | 1.15 (0.96, 1.38) | 1.15 (0.96, 1.38) | 1.15 (0.96, 1.38) | 1.15 (0.96, 1.38) | 1.15 (0.96, 1.38) | 1.17 (0.97, 1.40) | 1.16 (0.97, 1.39) |
|  | Other | 1.31 (0.93, 1.84) | 1.30 (0.92, 1.85) | 1.30 (0.92, 1.84) | 1.28 (0.90, 1.81) | 1.30 (0.92, 1.85) | 1.30 (0.92, 1.84) | 1.35 (0.95, 1.91) | 1.34 (0.94, 1.89) |
|  | White | Reference | Reference | Reference | Reference | Reference | Reference | Reference | Reference |
|  | Missing | 1.74 (0.85, 3.56) | 1.70 (0.82, 3.51) | 1.70 (0.82, 3.51) | 1.68 (0.82, 3.47) | 1.70 (0.83, 3.51) | 1.70 (0.83, 3.51) | 1.70 (0.83, 3.51) | 1.70 (0.82, 3.51) |
| EAL | No | Reference | Reference | Reference | Reference | Reference | Reference | Reference | Reference |
|  | Yes | 1.07 (0.92, 1.24) | 1.06 (0.91, 1.23) | 1.06 (0.91, 1.23) | 1.06 (0.91, 1.23) | 1.06 (0.91, 1.23) | 1.06 (0.91, 1.23) | 1.06 (0.91, 1.23) | 1.07 (0.92, 1.24) |
|  | Unclear or missing | 1.08 (0.48, 2.44) | 1.12 (0.49, 2.57) | 1.12 (0.49, 2.57) | 1.12 (0.49, 2.57) | 1.12 (0.49, 2.57) | 1.12 (0.49, 2.57) | 1.11 (0.49, 2.55) | 1.12 (0.49, 2.56) |
| Rate of hospitalisation (pre year 1) |  | 1.00 (1.00, 1.01) | 1.00 (1.00, 1.01) | 1.00 (1.00, 1.01) | 1.00 (1.00, 1.01) | 1.00 (1.00, 1.01) | 1.00 (1.00, 1.01) | 1.00 (1.00, 1.01) | 1.00 (1.00, 1.01) |
| Age at Year One start |  | 1.70 (1.47, 1.97) | 1.72 (1.48, 1.99) | 1.72 (1.48, 1.99) | 1.72 (1.49, 2.00) | 1.72 (1.48, 1.99) | 1.72 (1.48, 1.99) | 1.72 (1.49, 2.00) | 1.72 (1.48, 1.99) |
| School governance | Community | Reference | Reference | Reference | Reference | Reference | Reference | Reference | Reference |
|  | Sponsor led academy | 0.71 (0.57, 0.87) | 0.69 (0.56, 0.86) | 0.69 (0.56, 0.86) | 0.70 (0.57, 0.87) | 0.70 (0.56, 0.87) | 0.70 (0.57, 0.87) | 0.69 (0.56, 0.85) | 0.69 (0.56, 0.85) |
|  | Converter led academy | 0.86 (0.75, 0.99) | 0.84 (0.72, 0.97) | 0.84 (0.72, 0.97) | 0.84 (0.73, 0.98) | 0.85 (0.73, 0.98) | 0.85 (0.73, 0.99) | 0.84 (0.72, 0.97) | 0.84 (0.72, 0.97) |
|  | Free school | 1.03 (0.58, 1.83) | 1.01 (0.56, 1.81) | 1.01 (0.56, 1.80) | 1.04 (0.58, 1.85) | 1.01 (0.57, 1.81) | 1.01 (0.57, 1.81) | 1.00 (0.56, 1.80) | 1.02 (0.57, 1.83) |
|  | Voluntary aided | 0.56 (0.48, 0.64) | 0.53 (0.46, 0.61) | 0.53 (0.45, 0.61) | 0.53 (0.46, 0.61) | 0.53 (0.45, 0.61) | 0.53 (0.45, 0.61) | 0.53 (0.46, 0.62) | 0.53 (0.46, 0.62) |
|  | Voluntary controlled | 0.59 (0.49, 0.71) | 0.57 (0.47, 0.69) | 0.57 (0.47, 0.69) | 0.57 (0.47, 0.70) | 0.57 (0.47, 0.69) | 0.57 (0.47, 0.69) | 0.56 (0.46, 0.68) | 0.56 (0.46, 0.68) |
| Missing EYSFSP score | Yes vs. no (ref.) |  |  |  |  |  |  |  |  |
| Standardised EYSFP score |  |  |  |  |  |  |  |  |  |
| _cons |  | 0.00 (0.00, 0.00) | 0.00 (0.00, 0.00) | 0.00 (0.00, 0.00) | 0.00 (0.00, 0.00) | 0.00 (0.00, 0.00) | 0.00 (0.00, 0.00) | 0.00 (0.00, 0.00) | 0.00 (0.00, 0.00) |
| LA-specific associations | |  |  |  |  |  |  |  |  |
| Pupil population | Linear term |  |  | 1.00 (1.00, 1.00) |  |  |  |  |  |
|  | Quadratic term |  |  | 1.00 (1.00, 1.00) |  |  |  |  |  |
| Special school attendance | Linear term |  |  |  | 2.89 (1.44, 5.80) |  |  |  |  |
|  | Quadratic term |  |  |  | 0.73 (0.49, 1.09) |  |  |  |  |
| Maintained school attendance | Linear term |  |  |  |  | 1.00 (1.00, 1.01) |  |  |  |
| Academy attendance | Linear term |  |  |  |  |  | 1.00 (0.99, 1.00) |  |  |
| FSM eligible | Linear term |  |  |  |  |  |  | 0.98 (0.94, 1.01) |  |
|  | Quadratic term |  |  |  |  |  |  | 1.00 (1.00, 1.00) |  |
| IDACI mode groups | 1 Most deprived 20% |  |  |  |  |  |  |  | 0.73 (0.59, 0.90) |
|  | 2 |  |  |  |  |  |  |  | 0.89 (0.70, 1.13) |
|  | 3 |  |  |  |  |  |  |  | 0.67 (0.51, 0.88) |
|  | 4 |  |  |  |  |  |  |  | 0.98 (0.75, 1.27) |
|  | 5 Least deprived 20% |  |  |  |  |  |  |  | Reference |
| LA-general effects |  |  |  |  |  |  |  |  |  |
| LA variance (95% CI) |  |  | 0.17 (0.11, 0.25) | 0.16 (0.11, 0.24) | 0.14 (0.09, 0.21) | 0.17 (0.11, 0.25) | 0.17 (0.12, 0.25) | 0.17 (0.11, 0.24) | 0.14 (0.09, 0.21) |
| PCV* |  |  |  | 3.75% | 16.26% | -0.20% | -0.31% | 1.69% | 17.19% |
| AUC (95% CI) |  | 0.78 (0.77, 0.79) | 0.80 (0.79, 0.81) | 0.80 (0.79, 0.81) | 0.80 (0.79, 0.81) | 0.80 (0.79, 0.81) | 0.80 (0.79, 0.81) | 0.80 (0.79, 0.81) | 0.80 (0.79, 0.81) |
| AUC change* |  |  | 0.02 | 0.00 | 0.00 | 0.00 | 0.00 | 0.00 | 0.00 |
| ICC % (95% CI) |  |  | 4.86 (3.37, 6.97) | 4.69 (3.22, 6.77) | 4.10 (2.76, 6.07) | 4.87 (3.38, 6.98) | 4.88 (3.38, 6.99) | 4.78 (3.29, 6.91) | 4.06 (2.74, 5.97) |
| Model parameters/goodness of fit |  |  |  |  |  |  |  |  |  |
| N |  | 24111 | 24111 | 24111 | 24111 | 24111 | 24111 | 24111 | 24111 |
| Log likelihood |  | -7570.1 | -7503.5 | -7502.6 | -7489.7 | -7503.4 | -7503.2 | -7497.7 | -7496.1 |
| Degrees of freedom |  | 44 | 45 | 47 | 47 | 46 | 46 | 47 | 49 |
| AIC |  | 15228.1 | 15097.0 | 15099.2 | 15073.4 | 15098.7 | 15098.5 | 15089.5 | 15090.2 |
| AIC change* |  |  | -131.1 | 2.2 | -23.6 | 1.7 | 1.5 | -7.5 | -6.8 |
| BIC |  | 15584.1 | 15461.1 | 15479.4 | 15453.6 | 15470.9 | 15470.6 | 15469.7 | 15486.6 |
| BIC change* |  |  | -123.0 | 18.3 | -7.5 | 9.8 | 9.5 | 8.6 | 25.5 |
| **(c) EHCP vs. no SEND provision** |  | **Step one (single level** | **Step two (multilevel)** | **Step three (multilevel, + pupil headcount)** | **Step three (multilevel, + special school attendance %)** | **Step three (multilevel, + maintained school attendance %)** | **Step three (multilevel, + academy school attendance %)** | **Step three (multilevel, + FSM eligible %)** | **Step three (multilevel, + IDACI mode)** |
|  |  | cOR (95% CI) | cOR (95% CI) | cOR (95% CI) | cOR (95% CI) | cOR (95% CI) | cOR (95% CI) | cOR (95% CI) | cOR (95% CI) |
| Child-specific associations |  |  |  |  |  |  |  |  |  |
| Year of birth (1 September to 31 August) | 2003/04 | Reference | Reference | Reference | Reference | Reference | Reference | Reference | Reference |
|  | 2004/05 | 1.36 (1.09, 1.69) | 1.35 (1.08, 1.68) | 1.35 (1.09, 1.68) | 1.33 (1.07, 1.65) | 1.35 (1.08, 1.68) | 1.35 (1.08, 1.68) | 1.37 (1.10, 1.71) | 1.35 (1.09, 1.68) |
|  | 2005/06 | 1.07 (0.85, 1.34) | 1.07 (0.85, 1.34) | 1.07 (0.85, 1.35) | 1.06 (0.85, 1.34) | 1.07 (0.85, 1.34) | 1.07 (0.85, 1.34) | 1.10 (0.87, 1.38) | 1.07 (0.85, 1.35) |
|  | 2006/07 | 1.14 (0.91, 1.43) | 1.14 (0.90, 1.43) | 1.14 (0.91, 1.43) | 1.13 (0.90, 1.42) | 1.13 (0.90, 1.43) | 1.14 (0.90, 1.43) | 1.15 (0.92, 1.45) | 1.14 (0.90, 1.43) |
|  | 2007/08 | 1.23 (0.99, 1.53) | 1.22 (0.98, 1.51) | 1.23 (0.99, 1.52) | 1.15 (0.92, 1.43) | 1.22 (0.98, 1.52) | 1.22 (0.98, 1.53) | 1.20 (0.97, 1.50) | 1.23 (0.99, 1.53) |
|  | 2008/09 | 1.09 (0.88, 1.34) | 1.08 (0.88, 1.34) | 1.09 (0.88, 1.35) | 0.99 (0.80, 1.23) | 1.08 (0.87, 1.35) | 1.09 (0.87, 1.36) | 1.04 (0.84, 1.29) | 1.09 (0.88, 1.34) |
|  | 2009/10 | 1.03 (0.84, 1.27) | 1.02 (0.83, 1.26) | 1.03 (0.84, 1.27) | 0.92 (0.75, 1.14) | 1.02 (0.82, 1.28) | 1.03 (0.82, 1.28) | 0.94 (0.76, 1.16) | 1.03 (0.83, 1.27) |
|  | 2010/11 | 1.12 (0.91, 1.38) | 1.11 (0.90, 1.37) | 1.12 (0.91, 1.38) | 0.99 (0.80, 1.23) | 1.11 (0.88, 1.39) | 1.12 (0.89, 1.40) | 1.01 (0.81, 1.25) | 1.11 (0.90, 1.37) |
|  | 2011/12 | 1.29 (1.05, 1.58) | 1.29 (1.05, 1.58) | 1.30 (1.06, 1.60) | 1.12 (0.91, 1.38) | 1.28 (1.01, 1.62) | 1.30 (1.03, 1.63) | 1.16 (0.94, 1.44) | 1.29 (1.05, 1.58) |
|  | 2012/13 | 1.30 (1.07, 1.60) | 1.31 (1.07, 1.60) | 1.32 (1.07, 1.62) | 1.12 (0.91, 1.39) | 1.30 (1.02, 1.66) | 1.32 (1.04, 1.68) | 1.24 (1.01, 1.53) | 1.31 (1.07, 1.61) |
| Maternal age | Linear term | 0.97 (0.92, 1.02) | 0.97 (0.91, 1.02) | 0.97 (0.91, 1.02) | 0.97 (0.91, 1.02) | 0.97 (0.91, 1.02) | 0.97 (0.91, 1.02) | 0.97 (0.91, 1.02) | 0.97 (0.91, 1.02) |
|  | Quadratic term | 1.00 (1.00, 1.00) | 1.00 (1.00, 1.00) | 1.00 (1.00, 1.00) | 1.00 (1.00, 1.00) | 1.00 (1.00, 1.00) | 1.00 (1.00, 1.00) | 1.00 (1.00, 1.00) | 1.00 (1.00, 1.00) |
|  | Missing | 1.11 (0.43, 2.88) | 1.01 (0.39, 2.64) | 1.01 (0.39, 2.64) | 1.04 (0.40, 2.70) | 1.01 (0.39, 2.64) | 1.01 (0.39, 2.64) | 1.02 (0.39, 2.65) | 1.01 (0.39, 2.62) |
| Gender | Male vs. female (ref.) | 2.79 (2.54, 3.06) | 2.80 (2.55, 3.07) | 2.80 (2.55, 3.07) | 2.80 (2.55, 3.07) | 2.80 (2.55, 3.07) | 2.80 (2.55, 3.07) | 2.80 (2.55, 3.08) | 2.80 (2.55, 3.07) |
| Chronic condition (cancer/blood) | Yes vs. no (ref.) | 1.05 (0.79, 1.38) | 1.03 (0.78, 1.36) | 1.03 (0.78, 1.36) | 1.02 (0.77, 1.35) | 1.03 (0.78, 1.36) | 1.03 (0.78, 1.36) | 1.03 (0.78, 1.36) | 1.03 (0.78, 1.37) |
| Chronic condition (cardiovascular) | Yes vs. no (ref.) | 3.70 (3.07, 4.46) | 3.74 (3.10, 4.52) | 3.74 (3.10, 4.52) | 3.75 (3.11, 4.53) | 3.74 (3.10, 4.52) | 3.74 (3.10, 4.52) | 3.73 (3.09, 4.51) | 3.73 (3.09, 4.51) |
| Chronic condition (chronic infection) | Yes vs. no (ref.) | 0.54 (0.21, 1.38) | 0.55 (0.21, 1.43) | 0.55 (0.21, 1.43) | 0.56 (0.22, 1.44) | 0.55 (0.21, 1.43) | 0.55 (0.21, 1.43) | 0.54 (0.21, 1.42) | 0.54 (0.21, 1.42) |
| Chronic condition (mental health/developmental) | Yes vs. no (ref.) | 38.34 (32.65, 45.02) | 39.80 (33.80, 46.86) | 39.80 (33.80, 46.87) | 39.68 (33.71, 46.72) | 39.80 (33.80, 46.86) | 39.80 (33.80, 46.87) | 39.71 (33.72, 46.76) | 39.75 (33.76, 46.80) |
| Chronic condition (metabolic endocrine) | Yes vs. no (ref.) | 1.16 (1.00, 1.35) | 1.15 (0.99, 1.34) | 1.15 (0.99, 1.34) | 1.15 (0.99, 1.34) | 1.15 (0.99, 1.34) | 1.15 (0.99, 1.34) | 1.15 (0.99, 1.34) | 1.15 (0.99, 1.34) |
| Chronic condition (musculoskeletal skin) | Yes vs. no (ref.) | 2.42 (1.94, 3.00) | 2.47 (1.98, 3.07) | 2.47 (1.98, 3.07) | 2.45 (1.97, 3.05) | 2.47 (1.98, 3.07) | 2.47 (1.98, 3.07) | 2.48 (1.99, 3.08) | 2.48 (1.99, 3.08) |
| Chronic condition (neurological) | Yes vs. no (ref.) | 8.34 (7.51, 9.26) | 8.48 (7.63, 9.42) | 8.48 (7.63, 9.42) | 8.49 (7.64, 9.44) | 8.48 (7.63, 9.42) | 8.48 (7.63, 9.42) | 8.48 (7.63, 9.43) | 8.48 (7.63, 9.43) |
| Chronic condition (non-specific) | Yes vs. no (ref.) | 5.73 (4.94, 6.64) | 5.89 (5.07, 6.84) | 5.89 (5.07, 6.84) | 5.89 (5.07, 6.84) | 5.89 (5.07, 6.84) | 5.89 (5.07, 6.84) | 5.90 (5.08, 6.86) | 5.89 (5.07, 6.85) |
| Chronic condition (respiratory) | Yes vs. no (ref.) | 0.92 (0.79, 1.08) | 0.92 (0.79, 1.08) | 0.92 (0.79, 1.08) | 0.92 (0.79, 1.08) | 0.92 (0.79, 1.08) | 0.92 (0.79, 1.08) | 0.92 (0.79, 1.08) | 0.92 (0.79, 1.08) |
| IDACI | 1 Most deprived 20% | 1.24 (1.06, 1.45) | 1.36 (1.15, 1.60) | 1.36 (1.15, 1.60) | 1.33 (1.13, 1.57) | 1.36 (1.15, 1.60) | 1.36 (1.15, 1.60) | 1.43 (1.21, 1.69) | 1.42 (1.21, 1.68) |
|  | 2 | 1.32 (1.13, 1.53) | 1.36 (1.16, 1.59) | 1.36 (1.16, 1.59) | 1.35 (1.15, 1.58) | 1.36 (1.16, 1.59) | 1.36 (1.16, 1.59) | 1.39 (1.19, 1.62) | 1.39 (1.19, 1.62) |
|  | 3 | 1.30 (1.11, 1.52) | 1.32 (1.12, 1.54) | 1.32 (1.12, 1.54) | 1.32 (1.12, 1.54) | 1.32 (1.12, 1.54) | 1.32 (1.12, 1.54) | 1.33 (1.13, 1.56) | 1.34 (1.14, 1.57) |
|  | 4 | 1.13 (0.96, 1.33) | 1.12 (0.95, 1.32) | 1.12 (0.95, 1.32) | 1.12 (0.95, 1.32) | 1.12 (0.95, 1.32) | 1.12 (0.95, 1.32) | 1.13 (0.96, 1.33) | 1.13 (0.96, 1.33) |
|  | 5 Least deprived 20% | Reference | Reference | Reference | Reference | Reference | Reference | Reference | Reference |
|  | Missing | 14.41 (1.72, 120.42) | 15.23 (1.78, 130.21) | 15.21 (1.78, 130.01) | 15.79 (1.84, 135.19) | 15.23 (1.78, 130.21) | 15.23 (1.78, 130.21) | 15.44 (1.80, 132.50) | 15.37 (1.79, 131.88) |
| FSM eligible | Yes vs. no (ref.) | 1.75 (1.58, 1.94) | 1.75 (1.58, 1.94) | 1.75 (1.58, 1.94) | 1.74 (1.57, 1.93) | 1.75 (1.58, 1.94) | 1.75 (1.58, 1.94) | 1.77 (1.59, 1.96) | 1.75 (1.58, 1.94) |
| Racial-ethnic group | Asian | 1.02 (0.87, 1.19) | 1.07 (0.91, 1.26) | 1.07 (0.91, 1.26) | 1.05 (0.90, 1.24) | 1.07 (0.91, 1.26) | 1.07 (0.91, 1.26) | 1.09 (0.93, 1.28) | 1.08 (0.92, 1.27) |
|  | Black | 1.56 (1.31, 1.85) | 1.60 (1.34, 1.92) | 1.60 (1.34, 1.91) | 1.59 (1.33, 1.90) | 1.60 (1.34, 1.92) | 1.60 (1.34, 1.92) | 1.65 (1.38, 1.97) | 1.64 (1.37, 1.96) |
|  | Chinese | 1.12 (0.60, 2.07) | 1.17 (0.63, 2.16) | 1.17 (0.63, 2.15) | 1.16 (0.62, 2.14) | 1.17 (0.63, 2.16) | 1.17 (0.63, 2.16) | 1.18 (0.64, 2.18) | 1.17 (0.63, 2.16) |
|  | Mixed | 0.92 (0.77, 1.10) | 0.94 (0.79, 1.13) | 0.94 (0.79, 1.13) | 0.94 (0.78, 1.12) | 0.94 (0.79, 1.13) | 0.94 (0.79, 1.13) | 0.95 (0.80, 1.14) | 0.95 (0.79, 1.14) |
|  | Other | 0.93 (0.66, 1.31) | 0.92 (0.65, 1.31) | 0.92 (0.65, 1.31) | 0.91 (0.64, 1.29) | 0.92 (0.65, 1.31) | 0.92 (0.65, 1.31) | 0.95 (0.67, 1.35) | 0.94 (0.67, 1.34) |
|  | White | Reference | Reference | Reference | Reference | Reference | Reference | Reference | Reference |
|  | Missing | 1.41 (0.75, 2.65) | 1.40 (0.74, 2.63) | 1.40 (0.74, 2.64) | 1.42 (0.75, 2.67) | 1.40 (0.74, 2.63) | 1.40 (0.74, 2.63) | 1.40 (0.74, 2.64) | 1.39 (0.73, 2.62) |
| EAL | No | Reference | Reference | Reference | Reference | Reference | Reference | Reference | Reference |
|  | Yes | 1.15 (1.00, 1.32) | 1.14 (0.99, 1.32) | 1.14 (0.99, 1.32) | 1.15 (1.00, 1.32) | 1.14 (0.99, 1.32) | 1.14 (0.99, 1.32) | 1.14 (0.99, 1.32) | 1.15 (1.00, 1.33) |
|  | Unclear or missing | 1.21 (0.56, 2.63) | 1.18 (0.54, 2.56) | 1.18 (0.54, 2.56) | 1.18 (0.54, 2.57) | 1.18 (0.54, 2.56) | 1.18 (0.54, 2.56) | 1.19 (0.55, 2.58) | 1.18 (0.54, 2.56) |
| Rate of hospitalisation (pre year 1) |  | 1.01 (1.00, 1.01) | 1.01 (1.00, 1.01) | 1.01 (1.00, 1.01) | 1.01 (1.00, 1.01) | 1.01 (1.00, 1.01) | 1.01 (1.00, 1.01) | 1.01 (1.00, 1.01) | 1.01 (1.00, 1.01) |
| Age at Year One start |  | 0.74 (0.64, 0.86) | 0.73 (0.63, 0.85) | 0.73 (0.63, 0.85) | 0.73 (0.63, 0.85) | 0.73 (0.63, 0.85) | 0.73 (0.63, 0.85) | 0.73 (0.63, 0.85) | 0.73 (0.63, 0.85) |
| School governance | Community | Reference | Reference | Reference | Reference | Reference | Reference | Reference | Reference |
|  | Sponsor led academy | 0.69 (0.56, 0.85) | 0.69 (0.56, 0.86) | 0.69 (0.56, 0.86) | 0.70 (0.57, 0.86) | 0.69 (0.56, 0.86) | 0.69 (0.56, 0.86) | 0.69 (0.56, 0.85) | 0.69 (0.56, 0.85) |
|  | Converter led academy | 0.83 (0.72, 0.95) | 0.82 (0.71, 0.94) | 0.82 (0.71, 0.94) | 0.82 (0.71, 0.95) | 0.81 (0.70, 0.94) | 0.82 (0.71, 0.95) | 0.82 (0.71, 0.94) | 0.82 (0.71, 0.94) |
|  | Free school | 0.79 (0.46, 1.36) | 0.78 (0.45, 1.35) | 0.78 (0.45, 1.34) | 0.79 (0.45, 1.36) | 0.78 (0.45, 1.34) | 0.78 (0.45, 1.35) | 0.77 (0.45, 1.34) | 0.79 (0.46, 1.36) |
|  | Voluntary aided | 0.50 (0.43, 0.57) | 0.48 (0.41, 0.55) | 0.48 (0.41, 0.55) | 0.48 (0.41, 0.55) | 0.48 (0.41, 0.55) | 0.48 (0.41, 0.55) | 0.48 (0.41, 0.55) | 0.48 (0.41, 0.55) |
|  | Voluntary controlled | 0.53 (0.44, 0.64) | 0.51 (0.42, 0.62) | 0.51 (0.42, 0.62) | 0.51 (0.42, 0.62) | 0.51 (0.42, 0.62) | 0.51 (0.42, 0.62) | 0.50 (0.41, 0.61) | 0.50 (0.41, 0.61) |
| Missing EYSFSP score | Yes vs. no (ref.) |  |  |  |  |  |  |  |  |
| Standardised EYSFP score |  |  |  |  |  |  |  |  |  |
| _cons |  | 0.01 (0.00, 0.03) | 0.01 (0.00, 0.03) | 0.00 (0.00, 0.00) | 0.00 (0.00, 0.00) | 0.01 (0.00, 0.03) | 0.01 (0.00, 0.03) | 0.00 (0.00, 0.00) | 0.01 (0.00, 0.03) |
| LA-specific associations | |  |  |  |  |  |  |  |  |
| Pupil population | Linear term |  |  | 1.00 (1.00, 1.00) |  |  |  |  |  |
|  | Quadratic term |  |  | 1.00 (1.00, 1.00) |  |  |  |  |  |
| Special school attendance | Linear term |  |  |  | 2.53 (1.30, 4.95) |  |  |  |  |
|  | Quadratic term |  |  |  | 0.78 (0.53, 1.16) |  |  |  |  |
| Maintained school attendance | Linear term |  |  |  |  | 1.00 (1.00, 1.00) |  |  |  |
| Academy attendance | Linear term |  |  |  |  |  | 1.00 (1.00, 1.00) |  |  |
| FSM eligible | Linear term |  |  |  |  |  |  | 0.98 (0.95, 1.01) |  |
|  | Quadratic term |  |  |  |  |  |  | 1.00 (1.00, 1.00) |  |
| IDACI mode groups | 1 Most deprived 20% |  |  |  |  |  |  |  | 0.74 (0.61, 0.90) |
|  | 2 |  |  |  |  |  |  |  | 0.95 (0.76, 1.19) |
|  | 3 |  |  |  |  |  |  |  | 0.76 (0.59, 0.98) |
|  | 4 |  |  |  |  |  |  |  | 1.03 (0.80, 1.32) |
|  | 5 Least deprived 20% |  |  |  |  |  |  |  | Reference |
| LA-general effects |  |  |  |  |  |  |  |  |  |
| LA variance (95% CI) |  |  | 0.14 (0.09, 0.20) | 0.14 (0.09, 0.21) | 0.11 (0.07, 0.17) | 0.14 (0.09, 0.20) | 0.14 (0.09, 0.20) | 0.13 (0.09, 0.20) | 0.11 (0.07, 0.17) |
| PCV* |  |  |  | -0.11% | 17.90% | -0.01% | 0.01% | 2.65% | 17.51% |
| AUC (95% CI) |  | 0.85 (0.84, 0.85) | 0.86 (0.85, 0.87) | 0.86 (0.85, 0.87) | 0.86 (0.85, 0.87) | 0.86 (0.85, 0.87) | 0.86 (0.85, 0.87) | 0.86 (0.85, 0.87) | 0.86 (0.85, 0.87) |
| AUC change* |  |  | 0.02 | 0.00 | 0.00 | 0.00 | 0.00 | 0.00 | 0.00 |
| ICC % (95% CI) |  |  | 3.98 (2.69, 5.85) | 3.98 (2.68, 5.88) | 3.29 (2.13, 5.03) | 3.98 (2.69, 5.85) | 3.98 (2.69, 5.85) | 3.87 (2.59, 5.76) | 3.30 (2.17, 5.00) |
| Model parameters/goodness of fit |  |  |  |  |  |  |  |  |  |
| N |  | 125226 | 125226 | 125226 | 125226 | 125226 | 125226 | 125226 | 125226 |
| Log likelihood |  | -10189.3 | -10136.0 | -10135.8 | -10122.2 | -10136.0 | -10136.0 | -10129.3 | -10128.6 |
| Degrees of freedom |  | 44 | 45 | 47 | 47 | 46 | 46 | 47 | 49 |
| AIC |  | 20466.6 | 20362.0 | 20365.7 | 20338.4 | 20364.0 | 20364.0 | 20352.6 | 20355.3 |
| AIC change* |  |  | -104.6 | 5268.7 | 5241.4 | 5267.0 | 5267.0 | 5255.6 | 5258.3 |
| BIC |  | 20895.0 | 20800.2 | 20823.4 | 20796.1 | 20812.0 | 20812.0 | 20810.2 | 20832.5 |
| BIC change* |  |  | -94.8 | 5362.3 | 5335.0 | 5350.9 | 5350.9 | 5349.1 | 5371.4 |

AIC= Akaike information criterion; AUC = area under the receiving operator characteristic (ROC) curve; BIC = and Bayesian information criterion; CI = confidence interval; cOR = condition odds ratio; EAL = English as an additional language; EHCP = education, health and care plan; EYFSP = Early years foundation stage profile; FSM = free school meals; IDACI = Income deprivation affecting children index; IQR = interquartile range; LA = local authority; PCV=percentage change in variance; SEND=special educational needs and disability; *change in relation to the previous step (i.e. step 1 for step 2 model, step 2 model for step 3 models); Positive values for AUC change and PCV indicate increases in the discimination and variance explained compared with model from the previous step. Negative AIC/BIC change values indicates better model fit.

**Table S6.** Single- and multi-level logistic regression analysis of (a) SEND support vs. no SEND provision, (b) EHCP vs. SEND support and (c) EHCP vs. no SEND provision: children born full-term

| **(a) SEND support vs. no SEND provision** |  | **Step one (single level)** | **Step two (multilevel)** | **Step three (multilevel, + pupil headcount)** | **Step three (multilevel, + special school attendance %)** | **Step three (multilevel, + maintained school attendance %)** | **Step three (multilevel, + academy school attendance %)** | **Step three (multilevel, + FSM eligible %)** | **Step three (multilevel, + IDACI mode)** |
| --- | --- | --- | --- | --- | --- | --- | --- | --- | --- |
|  |  | **cOR (95% CI)** | **cOR (95% CI)** | **cOR (95% CI)** | **cOR (95% CI)** | **cOR (95% CI)** | **cOR (95% CI)** | **cOR (95% CI)** | **cOR (95% CI)** |
| *Child-specific associations* |  |  |  |  |  |  |  |  |  |
| Year of birth (1 September to 31 August) | 2003/04 | Reference | Reference | Reference | Reference | Reference | Reference | Reference | Reference |
|  | 2004/05 | 1.01 (0.93, 1.10) | 1.01 (0.92, 1.10) | 1.01 (0.92, 1.10) | 1.00 (0.92, 1.09) | 1.01 (0.92, 1.10) | 1.01 (0.92, 1.10) | 1.01 (0.93, 1.10) | 1.01 (0.92, 1.10) |
|  | 2005/06 | 0.89 (0.81, 0.97) | 0.88 (0.80, 0.97) | 0.88 (0.81, 0.97) | 0.88 (0.80, 0.96) | 0.89 (0.81, 0.98) | 0.89 (0.81, 0.98) | 0.89 (0.81, 0.97) | 0.88 (0.80, 0.96) |
|  | 2006/07 | 0.82 (0.75, 0.89) | 0.82 (0.75, 0.90) | 0.82 (0.75, 0.90) | 0.82 (0.75, 0.90) | 0.84 (0.77, 0.92) | 0.84 (0.77, 0.92) | 0.82 (0.75, 0.90) | 0.82 (0.75, 0.90) |
|  | 2007/08 | 0.70 (0.64, 0.77) | 0.71 (0.65, 0.77) | 0.71 (0.65, 0.78) | 0.70 (0.64, 0.76) | 0.74 (0.68, 0.81) | 0.74 (0.68, 0.81) | 0.70 (0.64, 0.77) | 0.71 (0.65, 0.77) |
|  | 2008/09 | 0.59 (0.54, 0.64) | 0.58 (0.54, 0.64) | 0.59 (0.54, 0.64) | 0.57 (0.52, 0.62) | 0.62 (0.57, 0.68) | 0.62 (0.57, 0.68) | 0.58 (0.53, 0.63) | 0.59 (0.54, 0.64) |
|  | 2009/10 | 0.53 (0.49, 0.58) | 0.53 (0.49, 0.58) | 0.54 (0.49, 0.59) | 0.52 (0.47, 0.57) | 0.57 (0.52, 0.63) | 0.57 (0.52, 0.63) | 0.52 (0.47, 0.57) | 0.53 (0.49, 0.58) |
|  | 2010/11 | 0.53 (0.49, 0.58) | 0.53 (0.49, 0.58) | 0.54 (0.49, 0.59) | 0.52 (0.47, 0.56) | 0.59 (0.53, 0.65) | 0.59 (0.53, 0.65) | 0.52 (0.47, 0.57) | 0.53 (0.49, 0.58) |
|  | 2011/12 | 0.53 (0.48, 0.58) | 0.53 (0.48, 0.57) | 0.53 (0.49, 0.58) | 0.51 (0.46, 0.56) | 0.59 (0.53, 0.65) | 0.59 (0.53, 0.65) | 0.51 (0.47, 0.56) | 0.53 (0.48, 0.57) |
|  | 2012/13 | 0.57 (0.52, 0.62) | 0.56 (0.51, 0.61) | 0.57 (0.52, 0.62) | 0.54 (0.49, 0.59) | 0.64 (0.57, 0.71) | 0.64 (0.57, 0.71) | 0.55 (0.50, 0.60) | 0.56 (0.51, 0.61) |
| Maternal age | Linear term | 0.99 (0.97, 1.02) | 0.99 (0.96, 1.01) | 0.99 (0.96, 1.01) | 0.99 (0.96, 1.01) | 0.99 (0.96, 1.02) | 0.99 (0.96, 1.02) | 0.99 (0.96, 1.01) | 0.99 (0.96, 1.01) |
|  | Quadratic term | 1.00 (1.00, 1.00) | 1.00 (1.00, 1.00) | 1.00 (1.00, 1.00) | 1.00 (1.00, 1.00) | 1.00 (1.00, 1.00) | 1.00 (1.00, 1.00) | 1.00 (1.00, 1.00) | 1.00 (1.00, 1.00) |
|  | Missing | 0.84 (0.54, 1.30) | 0.83 (0.54, 1.29) | 0.83 (0.54, 1.29) | 0.83 (0.53, 1.29) | 0.84 (0.54, 1.30) | 0.84 (0.54, 1.30) | 0.83 (0.53, 1.29) | 0.83 (0.54, 1.30) |
| Gender | Male vs. female (ref.) | 1.76 (1.69, 1.83) | 1.76 (1.69, 1.83) | 1.76 (1.69, 1.83) | 1.76 (1.69, 1.83) | 1.76 (1.69, 1.83) | 1.76 (1.69, 1.83) | 1.76 (1.69, 1.83) | 1.76 (1.69, 1.83) |
| Chronic condition (cancer/blood) | Yes vs. no (ref.) | 1.18 (0.98, 1.43) | 1.18 (0.97, 1.43) | 1.18 (0.97, 1.43) | 1.18 (0.97, 1.43) | 1.18 (0.97, 1.43) | 1.18 (0.97, 1.43) | 1.18 (0.97, 1.43) | 1.17 (0.97, 1.42) |
| Chronic condition (cardiovascular) | Yes vs. no (ref.) | 1.30 (1.06, 1.58) | 1.28 (1.05, 1.56) | 1.28 (1.05, 1.56) | 1.28 (1.05, 1.56) | 1.28 (1.05, 1.56) | 1.28 (1.05, 1.57) | 1.28 (1.05, 1.56) | 1.28 (1.05, 1.56) |
| Chronic condition (chronic infection) | Yes vs. no (ref.) | 0.90 (0.52, 1.54) | 0.90 (0.53, 1.54) | 0.90 (0.53, 1.54) | 0.90 (0.53, 1.54) | 0.90 (0.53, 1.54) | 0.90 (0.53, 1.54) | 0.91 (0.53, 1.55) | 0.91 (0.53, 1.55) |
| Chronic condition (mental health/developmental) | Yes vs. no (ref.) | 4.34 (3.51, 5.36) | 4.39 (3.55, 5.43) | 4.39 (3.55, 5.43) | 4.39 (3.55, 5.43) | 4.40 (3.56, 5.43) | 4.40 (3.56, 5.43) | 4.39 (3.55, 5.43) | 4.39 (3.55, 5.43) |
| Chronic condition (metabolic endocrine) | Yes vs. no (ref.) | 1.16 (1.06, 1.27) | 1.17 (1.07, 1.28) | 1.17 (1.07, 1.28) | 1.17 (1.07, 1.28) | 1.17 (1.07, 1.28) | 1.17 (1.07, 1.28) | 1.17 (1.07, 1.28) | 1.17 (1.07, 1.28) |
| Chronic condition (musculoskeletal skin) | Yes vs. no (ref.) | 1.68 (1.43, 1.98) | 1.70 (1.44, 2.00) | 1.70 (1.44, 2.00) | 1.70 (1.44, 2.00) | 1.70 (1.45, 2.00) | 1.70 (1.45, 2.00) | 1.70 (1.45, 2.00) | 1.70 (1.44, 2.00) |
| Chronic condition (neurological) | Yes vs. no (ref.) | 1.90 (1.74, 2.07) | 1.90 (1.74, 2.08) | 1.90 (1.74, 2.08) | 1.90 (1.74, 2.08) | 1.90 (1.74, 2.08) | 1.90 (1.74, 2.08) | 1.90 (1.74, 2.08) | 1.90 (1.74, 2.08) |
| Chronic condition (non-specific) | Yes vs. no (ref.) | 1.41 (1.20, 1.64) | 1.40 (1.19, 1.64) | 1.40 (1.19, 1.64) | 1.40 (1.19, 1.64) | 1.40 (1.19, 1.64) | 1.40 (1.19, 1.64) | 1.40 (1.19, 1.64) | 1.40 (1.19, 1.63) |
| Chronic condition (respiratory) | Yes vs. no (ref.) | 1.20 (1.10, 1.31) | 1.20 (1.09, 1.31) | 1.20 (1.09, 1.31) | 1.20 (1.09, 1.31) | 1.20 (1.09, 1.31) | 1.20 (1.09, 1.31) | 1.20 (1.09, 1.31) | 1.20 (1.09, 1.31) |
| IDACI | 1 Most deprived 20% | 1.33 (1.24, 1.43) | 1.35 (1.25, 1.45) | 1.35 (1.25, 1.45) | 1.34 (1.25, 1.45) | 1.35 (1.25, 1.45) | 1.35 (1.25, 1.45) | 1.36 (1.26, 1.46) | 1.36 (1.26, 1.47) |
|  | 2 | 1.21 (1.13, 1.30) | 1.22 (1.14, 1.31) | 1.22 (1.13, 1.31) | 1.22 (1.13, 1.31) | 1.22 (1.14, 1.31) | 1.22 (1.14, 1.31) | 1.22 (1.14, 1.32) | 1.23 (1.14, 1.32) |
|  | 3 | 1.17 (1.09, 1.25) | 1.16 (1.08, 1.25) | 1.16 (1.08, 1.25) | 1.16 (1.08, 1.25) | 1.16 (1.08, 1.25) | 1.16 (1.08, 1.25) | 1.16 (1.08, 1.25) | 1.16 (1.08, 1.25) |
|  | 4 | 1.06 (0.98, 1.14) | 1.06 (0.98, 1.15) | 1.06 (0.98, 1.15) | 1.06 (0.98, 1.15) | 1.06 (0.99, 1.15) | 1.06 (0.99, 1.15) | 1.06 (0.98, 1.15) | 1.06 (0.99, 1.15) |
|  | 5 Least deprived 20% | Reference | Reference | Reference | Reference | Reference | Reference | Reference | Reference |
|  | Missing | 1.00 (0.00, 0.00) | 1.00 (0.00, 0.00) | 1.00 (0.00, 0.00) | 1.00 (0.00, 0.00) | 1.00 (0.00, 0.00) | 1.00 (0.00, 0.00) | 1.00 (0.00, 0.00) | 1.00 (0.00, 0.00) |
| FSM eligible | Yes vs. no (ref.) | 1.34 (1.27, 1.40) | 1.33 (1.27, 1.39) | 1.33 (1.27, 1.39) | 1.33 (1.27, 1.39) | 1.33 (1.27, 1.39) | 1.33 (1.27, 1.39) | 1.33 (1.27, 1.40) | 1.33 (1.27, 1.39) |
| Racial-ethnic group | Asian | 0.85 (0.78, 0.91) | 0.83 (0.76, 0.90) | 0.83 (0.76, 0.90) | 0.83 (0.76, 0.90) | 0.83 (0.76, 0.90) | 0.83 (0.76, 0.90) | 0.83 (0.77, 0.90) | 0.83 (0.77, 0.90) |
|  | Black | 1.15 (1.06, 1.26) | 1.06 (0.97, 1.16) | 1.06 (0.96, 1.16) | 1.06 (0.97, 1.16) | 1.06 (0.96, 1.16) | 1.05 (0.96, 1.15) | 1.06 (0.97, 1.17) | 1.06 (0.97, 1.16) |
|  | Chinese | 0.71 (0.51, 0.99) | 0.72 (0.51, 1.00) | 0.71 (0.51, 1.00) | 0.71 (0.51, 0.99) | 0.71 (0.51, 0.99) | 0.71 (0.51, 0.99) | 0.72 (0.52, 1.00) | 0.72 (0.52, 1.00) |
|  | Mixed | 0.93 (0.85, 1.01) | 0.89 (0.82, 0.97) | 0.89 (0.82, 0.97) | 0.89 (0.82, 0.97) | 0.89 (0.82, 0.97) | 0.89 (0.82, 0.97) | 0.89 (0.82, 0.97) | 0.89 (0.82, 0.97) |
|  | Other | 0.92 (0.79, 1.08) | 0.86 (0.74, 1.01) | 0.86 (0.73, 1.01) | 0.86 (0.73, 1.01) | 0.86 (0.73, 1.01) | 0.86 (0.73, 1.01) | 0.87 (0.74, 1.02) | 0.87 (0.74, 1.01) |
|  | White | Reference | Reference | Reference | Reference | Reference | Reference | Reference | Reference |
|  | Missing | 0.93 (0.66, 1.30) | 0.90 (0.64, 1.26) | 0.90 (0.64, 1.26) | 0.90 (0.64, 1.26) | 0.90 (0.64, 1.26) | 0.90 (0.64, 1.26) | 0.90 (0.64, 1.26) | 0.90 (0.64, 1.26) |
| EAL | No | Reference | Reference | Reference | Reference | Reference | Reference | Reference | Reference |
|  | Yes | 0.71 (0.67, 0.76) | 0.68 (0.64, 0.73) | 0.68 (0.64, 0.73) | 0.68 (0.64, 0.73) | 0.68 (0.64, 0.73) | 0.68 (0.64, 0.73) | 0.68 (0.64, 0.73) | 0.69 (0.64, 0.73) |
|  | Unclear or missing | 0.96 (0.67, 1.39) | 0.93 (0.64, 1.35) | 0.93 (0.64, 1.35) | 0.94 (0.65, 1.35) | 0.93 (0.64, 1.35) | 0.93 (0.64, 1.35) | 0.93 (0.65, 1.35) | 0.93 (0.65, 1.35) |
| Rate of hospitalisation (pre year 1) |  | 1.01 (1.01, 1.02) | 1.01 (1.01, 1.02) | 1.01 (1.01, 1.02) | 1.01 (1.01, 1.02) | 1.01 (1.01, 1.02) | 1.01 (1.01, 1.02) | 1.01 (1.01, 1.02) | 1.01 (1.01, 1.02) |
| Age at Year One start |  | 1.27 (1.19, 1.36) | 1.29 (1.21, 1.38) | 1.29 (1.21, 1.39) | 1.30 (1.21, 1.39) | 1.29 (1.21, 1.39) | 1.29 (1.21, 1.39) | 1.29 (1.21, 1.38) | 1.29 (1.21, 1.38) |
| School governance | Community | Reference | Reference | Reference | Reference | Reference | Reference | Reference | Reference |
|  | Sponsor led academy | 0.93 (0.85, 1.02) | 0.96 (0.87, 1.05) | 0.96 (0.87, 1.05) | 0.96 (0.87, 1.05) | 0.98 (0.89, 1.08) | 0.98 (0.89, 1.08) | 0.95 (0.87, 1.05) | 0.96 (0.87, 1.05) |
|  | Converter led academy | 0.90 (0.84, 0.96) | 0.92 (0.86, 0.99) | 0.92 (0.86, 0.99) | 0.92 (0.86, 0.99) | 0.95 (0.89, 1.02) | 0.95 (0.89, 1.02) | 0.92 (0.86, 0.99) | 0.92 (0.86, 0.99) |
|  | Free school | 0.95 (0.72, 1.24) | 0.90 (0.69, 1.19) | 0.90 (0.69, 1.19) | 0.91 (0.69, 1.19) | 0.91 (0.69, 1.21) | 0.91 (0.69, 1.20) | 0.90 (0.68, 1.19) | 0.91 (0.69, 1.19) |
|  | Voluntary aided | 0.91 (0.86, 0.96) | 0.91 (0.86, 0.96) | 0.91 (0.86, 0.96) | 0.91 (0.86, 0.96) | 0.91 (0.86, 0.96) | 0.91 (0.86, 0.96) | 0.91 (0.86, 0.97) | 0.91 (0.86, 0.96) |
|  | Voluntary controlled | 0.88 (0.81, 0.94) | 0.88 (0.82, 0.95) | 0.88 (0.82, 0.95) | 0.88 (0.82, 0.95) | 0.88 (0.82, 0.95) | 0.88 (0.82, 0.95) | 0.88 (0.82, 0.95) | 0.88 (0.82, 0.95) |
| Missing EYSFSP score | Yes vs. no (ref.) | 2.27 (1.94, 2.67) | 2.26 (1.92, 2.65) | 2.26 (1.92, 2.65) | 2.26 (1.92, 2.65) | 2.26 (1.92, 2.65) | 2.26 (1.92, 2.65) | 2.26 (1.93, 2.65) | 2.26 (1.92, 2.65) |
| Standardised EYSFP score |  | 0.22 (0.22, 0.23) | 0.22 (0.21, 0.22) | 0.22 (0.21, 0.22) | 0.22 (0.21, 0.22) | 0.22 (0.21, 0.22) | 0.22 (0.21, 0.22) | 0.22 (0.21, 0.22) | 0.22 (0.21, 0.22) |
| _cons |  | 0.01 (0.01, 0.02) | 0.01 (0.01, 0.02) | 0.01 (0.01, 0.02) | 0.01 (0.01, 0.02) | 0.01 (0.00, 0.01) | 0.01 (0.01, 0.02) | 0.01 (0.01, 0.02) | 0.01 (0.01, 0.02) |
| *LA-specific associations* | |  |  |  |  |  |  |  |  |
| Pupil population | Linear term |  |  | 1.00 (1.00, 1.00) |  |  |  |  |  |
|  | Quadratic term |  |  | 1.00 (1.00, 1.00) |  |  |  |  |  |
| Special school attendance | Linear term |  |  |  | 1.28 (0.93, 1.76) |  |  |  |  |
|  | Quadratic term |  |  |  | 0.93 (0.77, 1.12) |  |  |  |  |
| Maintained school attendance | Linear term |  |  |  |  | 1.00 (1.00, 1.01) |  |  |  |
| Academy attendance | Linear term |  |  |  |  |  | 1.00 (0.99, 1.00) |  |  |
| FSM eligible | Linear term |  |  |  |  |  |  | 1.00 (0.98, 1.01) |  |
|  | Quadratic term |  |  |  |  |  |  | 1.00 (1.00, 1.00) |  |
| IDACI mode groups | 1 Most deprived 20% |  |  |  |  |  |  |  | 0.87 (0.78, 0.98) |
|  | 2 |  |  |  |  |  |  |  | 0.96 (0.84, 1.09) |
|  | 3 |  |  |  |  |  |  |  | 0.95 (0.82, 1.10) |
|  | 4 |  |  |  |  |  |  |  | 0.87 (0.76, 1.00) |
|  | 5 Least deprived 20% |  |  |  |  |  |  |  | Reference |
| *LA-general effects* |  |  |  |  |  |  |  |  |  |
| LA variance (95% CI) |  |  | 0.07 (0.05, 0.09) | 0.07 (0.05, 0.09) | 0.07 (0.05, 0.09) | 0.07 (0.05, 0.09) | 0.07 (0.05, 0.09) | 0.07 (0.05, 0.09) | 0.07 (0.05, 0.09) |
| PCV* |  |  |  | -2.27% | 2.05% | 0.81% | 1.54% | 1.03% | -0.92% |
| AUC (95% CI) |  | 0.86 (0.85, 0.86) | 0.86 (0.86, 0.86) | 0.86 (0.86, 0.86) | 0.86 (0.86, 0.86) | 0.86 (0.86, 0.86) | 0.86 (0.86, 0.86) | 0.86 (0.86, 0.86) | 0.86 (0.86, 0.86) |
| AUC change* |  |  | 0.004 | 0.000 | 0.000 | 0.000 | 0.000 | 0.000 | 0.000 |
| ICC % (95% CI) |  |  | 2.03 (1.53, 2.70) | 2.08 (1.56, 2.76) | 1.99 (1.50, 2.65) | 2.02 (1.52, 2.68) | 2.00 (1.51, 2.66) | 2.01 (1.51, 2.68) | 2.05 (1.54, 2.73) |
| *Model parameters/goodness of fit* |  |  |  |  |  |  |  |  |  |
| N |  | 140866 | 140866 | 140866 | 140866 | 140866 | 140866 | 140866 | 140866 |
| Log likelihood |  | -37394.4 | -37160.3 | -37159.6 | -37157.1 | -37153.2 | -37152.3 | -37158.4 | -37156.2 |
| Degrees of freedom |  | 45 | 46 | 48 | 48 | 47 | 47 | 48 | 50 |
| AIC |  | 74878.8 | 74412.5 | 74415.1 | 74410.3 | 74400.4 | 74398.7 | 74412.8 | 74412.4 |
| AIC change* |  |  | -466.3 | 2.6 | -2.2 | -12.1 | -13.8 | 0.3 | -0.1 |
| BIC |  | 75322.3 | 74865.9 | 74888.2 | 74883.4 | 74863.6 | 74861.9 | 74885.8 | 74905.2 |
| BIC change* |  |  | -456.4 | 22.3 | 17.5 | -2.3 | -4.0 | 19.9 | 39.3 |
| **(b) EHCP vs. SEND support** |  | **Step one (single level** | **Step two (multilevel)** | **Step three (multilevel, + pupil headcount)** | **Step three (multilevel, + special school attendance %)** | **Step three (multilevel, + maintained school attendance %)** | **Step three (multilevel, + academy school attendance %)** | **Step three (multilevel, + FSM eligible %)** | **Step three (multilevel, + IDACI mode)** |
|  |  | cOR (95% CI) | cOR (95% CI) | cOR (95% CI) | cOR (95% CI) | cOR (95% CI) | cOR (95% CI) | cOR (95% CI) | cOR (95% CI) |
| Child-specific associations |  |  |  |  |  |  |  |  |  |
| Year of birth (1 September to 31 August) | 2003/04 | Reference | Reference | Reference | Reference | Reference | Reference | Reference | Reference |
|  | 2004/05 | 0.96 (0.74, 1.25) | 0.96 (0.74, 1.25) | 0.96 (0.74, 1.25) | 0.95 (0.73, 1.24) | 0.96 (0.74, 1.25) | 0.96 (0.74, 1.25) | 0.99 (0.76, 1.29) | 0.96 (0.74, 1.25) |
|  | 2005/06 | 1.20 (0.93, 1.55) | 1.20 (0.93, 1.56) | 1.21 (0.94, 1.57) | 1.21 (0.93, 1.56) | 1.21 (0.93, 1.57) | 1.21 (0.93, 1.57) | 1.26 (0.97, 1.64) | 1.22 (0.94, 1.57) |
|  | 2006/07 | 1.13 (0.87, 1.47) | 1.13 (0.87, 1.48) | 1.14 (0.87, 1.48) | 1.13 (0.86, 1.47) | 1.14 (0.87, 1.49) | 1.14 (0.87, 1.49) | 1.17 (0.90, 1.53) | 1.13 (0.87, 1.48) |
|  | 2007/08 | 1.26 (0.98, 1.61) | 1.26 (0.98, 1.61) | 1.26 (0.98, 1.62) | 1.21 (0.94, 1.56) | 1.27 (0.98, 1.64) | 1.27 (0.98, 1.64) | 1.28 (1.00, 1.65) | 1.27 (0.99, 1.63) |
|  | 2008/09 | 1.38 (1.09, 1.75) | 1.40 (1.10, 1.79) | 1.41 (1.11, 1.79) | 1.33 (1.04, 1.70) | 1.43 (1.11, 1.84) | 1.43 (1.11, 1.84) | 1.40 (1.10, 1.78) | 1.42 (1.12, 1.81) |
|  | 2009/10 | 1.27 (1.00, 1.62) | 1.29 (1.01, 1.64) | 1.29 (1.01, 1.64) | 1.20 (0.94, 1.53) | 1.31 (1.01, 1.70) | 1.31 (1.01, 1.70) | 1.20 (0.93, 1.53) | 1.30 (1.02, 1.66) |
|  | 2010/11 | 1.50 (1.19, 1.90) | 1.53 (1.20, 1.93) | 1.52 (1.20, 1.94) | 1.41 (1.11, 1.79) | 1.56 (1.20, 2.03) | 1.56 (1.21, 2.03) | 1.41 (1.10, 1.79) | 1.54 (1.22, 1.95) |
|  | 2011/12 | 1.62 (1.29, 2.05) | 1.64 (1.29, 2.08) | 1.64 (1.29, 2.08) | 1.47 (1.15, 1.88) | 1.69 (1.29, 2.21) | 1.69 (1.29, 2.21) | 1.50 (1.18, 1.92) | 1.66 (1.31, 2.10) |
|  | 2012/13 | 1.83 (1.45, 2.30) | 1.86 (1.47, 2.36) | 1.86 (1.47, 2.35) | 1.65 (1.29, 2.11) | 1.93 (1.46, 2.55) | 1.93 (1.46, 2.54) | 1.84 (1.45, 2.33) | 1.89 (1.49, 2.39) |
| Maternal age | Linear term | 1.03 (0.97, 1.10) | 1.04 (0.97, 1.11) | 1.04 (0.97, 1.11) | 1.04 (0.97, 1.11) | 1.04 (0.97, 1.11) | 1.04 (0.97, 1.11) | 1.04 (0.97, 1.11) | 1.04 (0.97, 1.11) |
|  | Quadratic term | 1.00 (1.00, 1.00) | 1.00 (1.00, 1.00) | 1.00 (1.00, 1.00) | 1.00 (1.00, 1.00) | 1.00 (1.00, 1.00) | 1.00 (1.00, 1.00) | 1.00 (1.00, 1.00) | 1.00 (1.00, 1.00) |
|  | Missing | 1.17 (0.33, 4.14) | 1.08 (0.30, 3.88) | 1.06 (0.29, 3.82) | 1.08 (0.30, 3.89) | 1.08 (0.30, 3.88) | 1.08 (0.30, 3.88) | 1.06 (0.29, 3.82) | 1.08 (0.30, 3.88) |
| Gender | Male vs. female (ref.) | 1.40 (1.25, 1.57) | 1.40 (1.25, 1.57) | 1.40 (1.25, 1.57) | 1.40 (1.25, 1.57) | 1.40 (1.25, 1.57) | 1.40 (1.25, 1.57) | 1.40 (1.25, 1.58) | 1.40 (1.25, 1.57) |
| Chronic condition (cancer/blood) | Yes vs. no (ref.) | 0.90 (0.64, 1.27) | 0.93 (0.66, 1.31) | 0.93 (0.66, 1.31) | 0.92 (0.65, 1.30) | 0.93 (0.66, 1.31) | 0.93 (0.66, 1.31) | 0.92 (0.65, 1.30) | 0.93 (0.66, 1.32) |
| Chronic condition (cardiovascular) | Yes vs. no (ref.) | 1.74 (1.30, 2.32) | 1.76 (1.31, 2.36) | 1.77 (1.32, 2.37) | 1.76 (1.31, 2.37) | 1.76 (1.31, 2.37) | 1.76 (1.31, 2.37) | 1.76 (1.31, 2.37) | 1.76 (1.31, 2.36) |
| Chronic condition (chronic infection) | Yes vs. no (ref.) | 1.23 (0.50, 3.00) | 1.38 (0.56, 3.37) | 1.38 (0.56, 3.38) | 1.38 (0.57, 3.37) | 1.37 (0.56, 3.36) | 1.37 (0.56, 3.36) | 1.35 (0.55, 3.31) | 1.34 (0.55, 3.28) |
| Chronic condition (mental health/developmental) | Yes vs. no (ref.) | 6.65 (5.60, 7.90) | 6.91 (5.79, 8.23) | 6.91 (5.79, 8.23) | 6.90 (5.78, 8.22) | 6.91 (5.80, 8.24) | 6.91 (5.80, 8.24) | 6.96 (5.83, 8.29) | 6.89 (5.78, 8.21) |
| Chronic condition (metabolic endocrine) | Yes vs. no (ref.) | 1.12 (0.92, 1.36) | 1.12 (0.92, 1.36) | 1.12 (0.92, 1.36) | 1.11 (0.91, 1.35) | 1.12 (0.92, 1.36) | 1.12 (0.92, 1.36) | 1.12 (0.92, 1.36) | 1.12 (0.92, 1.36) |
| Chronic condition (musculoskeletal skin) | Yes vs. no (ref.) | 1.41 (1.07, 1.87) | 1.43 (1.07, 1.90) | 1.43 (1.07, 1.90) | 1.44 (1.08, 1.91) | 1.43 (1.07, 1.90) | 1.43 (1.07, 1.90) | 1.43 (1.08, 1.90) | 1.43 (1.08, 1.90) |
| Chronic condition (neurological) | Yes vs. no (ref.) | 2.64 (2.29, 3.03) | 2.71 (2.35, 3.13) | 2.71 (2.35, 3.13) | 2.72 (2.36, 3.14) | 2.71 (2.35, 3.13) | 2.72 (2.35, 3.13) | 2.71 (2.35, 3.13) | 2.72 (2.35, 3.14) |
| Chronic condition (non-specific) | Yes vs. no (ref.) | 3.62 (2.98, 4.40) | 3.72 (3.05, 4.54) | 3.73 (3.05, 4.55) | 3.74 (3.06, 4.57) | 3.72 (3.05, 4.55) | 3.72 (3.05, 4.55) | 3.71 (3.04, 4.53) | 3.72 (3.05, 4.54) |
| Chronic condition (respiratory) | Yes vs. no (ref.) | 0.74 (0.61, 0.90) | 0.73 (0.60, 0.90) | 0.73 (0.60, 0.90) | 0.73 (0.60, 0.90) | 0.73 (0.60, 0.90) | 0.73 (0.60, 0.90) | 0.73 (0.60, 0.90) | 0.73 (0.60, 0.90) |
| IDACI | 1 Most deprived 20% | 0.59 (0.49, 0.70) | 0.64 (0.53, 0.77) | 0.63 (0.52, 0.76) | 0.63 (0.52, 0.76) | 0.64 (0.53, 0.77) | 0.64 (0.53, 0.77) | 0.68 (0.56, 0.83) | 0.68 (0.56, 0.83) |
|  | 2 | 0.75 (0.63, 0.89) | 0.77 (0.65, 0.93) | 0.77 (0.64, 0.92) | 0.77 (0.64, 0.92) | 0.77 (0.65, 0.93) | 0.77 (0.65, 0.93) | 0.80 (0.67, 0.97) | 0.80 (0.67, 0.96) |
|  | 3 | 0.76 (0.63, 0.92) | 0.77 (0.64, 0.93) | 0.77 (0.64, 0.93) | 0.77 (0.64, 0.93) | 0.77 (0.64, 0.93) | 0.77 (0.64, 0.93) | 0.79 (0.65, 0.95) | 0.79 (0.65, 0.95) |
|  | 4 | 0.90 (0.74, 1.08) | 0.90 (0.74, 1.09) | 0.90 (0.74, 1.09) | 0.91 (0.75, 1.10) | 0.90 (0.74, 1.09) | 0.90 (0.74, 1.09) | 0.91 (0.75, 1.10) | 0.91 (0.75, 1.10) |
|  | 5 Least deprived 20% | Reference | Reference | Reference | Reference | Reference | Reference | Reference | Reference |
|  | Missing | 1.00 (0.00, 0.00) | 1.00 (0.00, 0.00) | 1.00 (0.00, 0.00) | 1.00 (0.00, 0.00) | 1.00 (0.00, 0.00) | 1.00 (0.00, 0.00) | 1.00 (0.00, 0.00) | 1.00 (0.00, 0.00) |
| FSM eligible | Yes vs. no (ref.) | 1.04 (0.93, 1.17) | 1.03 (0.92, 1.16) | 1.03 (0.92, 1.16) | 1.03 (0.92, 1.16) | 1.03 (0.92, 1.16) | 1.03 (0.92, 1.16) | 1.04 (0.92, 1.17) | 1.04 (0.92, 1.17) |
| Racial-ethnic group | Asian | 1.15 (0.94, 1.40) | 1.17 (0.95, 1.44) | 1.17 (0.95, 1.44) | 1.17 (0.94, 1.44) | 1.17 (0.95, 1.44) | 1.17 (0.95, 1.44) | 1.19 (0.96, 1.47) | 1.20 (0.97, 1.48) |
|  | Black | 1.85 (1.52, 2.25) | 1.93 (1.57, 2.38) | 1.92 (1.56, 2.36) | 1.93 (1.57, 2.38) | 1.93 (1.56, 2.37) | 1.93 (1.56, 2.37) | 1.98 (1.61, 2.44) | 2.01 (1.63, 2.47) |
|  | Chinese | 1.92 (0.98, 3.78) | 1.97 (0.99, 3.91) | 1.96 (0.99, 3.90) | 1.96 (0.99, 3.89) | 1.97 (0.99, 3.91) | 1.97 (0.99, 3.91) | 2.00 (1.01, 3.96) | 1.98 (1.00, 3.94) |
|  | Mixed | 0.93 (0.74, 1.18) | 0.93 (0.73, 1.18) | 0.92 (0.72, 1.17) | 0.92 (0.72, 1.17) | 0.93 (0.73, 1.18) | 0.93 (0.73, 1.18) | 0.93 (0.73, 1.19) | 0.94 (0.74, 1.20) |
|  | Other | 1.06 (0.71, 1.59) | 1.06 (0.70, 1.59) | 1.05 (0.69, 1.58) | 1.04 (0.69, 1.57) | 1.05 (0.70, 1.59) | 1.05 (0.70, 1.59) | 1.07 (0.71, 1.62) | 1.10 (0.73, 1.66) |
|  | White | Reference | Reference | Reference | Reference | Reference | Reference | Reference | Reference |
|  | Missing | 1.28 (0.57, 2.90) | 1.30 (0.56, 2.98) | 1.29 (0.56, 2.96) | 1.30 (0.56, 2.99) | 1.30 (0.56, 2.98) | 1.30 (0.56, 2.98) | 1.32 (0.58, 3.02) | 1.31 (0.57, 3.02) |
| EAL | No | Reference | Reference | Reference | Reference | Reference | Reference | Reference | Reference |
|  | Yes | 1.10 (0.93, 1.31) | 1.08 (0.91, 1.29) | 1.08 (0.91, 1.29) | 1.08 (0.91, 1.29) | 1.08 (0.91, 1.29) | 1.08 (0.91, 1.29) | 1.08 (0.91, 1.29) | 1.10 (0.92, 1.31) |
|  | Unclear or missing | 1.04 (0.42, 2.53) | 0.98 (0.40, 2.44) | 0.98 (0.39, 2.43) | 0.99 (0.40, 2.46) | 0.98 (0.40, 2.44) | 0.98 (0.40, 2.44) | 0.99 (0.40, 2.45) | 0.99 (0.40, 2.45) |
| Rate of hospitalisation (pre year 1) |  | 1.00 (1.00, 1.01) | 1.00 (1.00, 1.01) | 1.00 (1.00, 1.01) | 1.00 (1.00, 1.01) | 1.00 (1.00, 1.01) | 1.00 (1.00, 1.01) | 1.00 (1.00, 1.01) | 1.00 (1.00, 1.01) |
| Age at Year One start |  | 1.76 (1.49, 2.09) | 1.80 (1.51, 2.13) | 1.79 (1.51, 2.13) | 1.80 (1.51, 2.13) | 1.80 (1.51, 2.13) | 1.80 (1.51, 2.13) | 1.80 (1.51, 2.13) | 1.80 (1.51, 2.13) |
| School governance | Community | Reference | Reference | Reference | Reference | Reference | Reference | Reference | Reference |
|  | Sponsor led academy | 0.63 (0.49, 0.82) | 0.62 (0.48, 0.81) | 0.62 (0.48, 0.81) | 0.63 (0.48, 0.81) | 0.63 (0.48, 0.81) | 0.63 (0.48, 0.81) | 0.62 (0.48, 0.80) | 0.62 (0.48, 0.80) |
|  | Converter led academy | 0.90 (0.77, 1.06) | 0.89 (0.75, 1.05) | 0.89 (0.75, 1.05) | 0.90 (0.76, 1.06) | 0.90 (0.75, 1.07) | 0.90 (0.75, 1.07) | 0.89 (0.75, 1.05) | 0.89 (0.75, 1.05) |
|  | Free school | 0.83 (0.40, 1.71) | 0.83 (0.39, 1.72) | 0.83 (0.40, 1.72) | 0.83 (0.40, 1.74) | 0.83 (0.40, 1.73) | 0.83 (0.39, 1.73) | 0.82 (0.39, 1.71) | 0.83 (0.40, 1.73) |
|  | Voluntary aided | 0.56 (0.47, 0.67) | 0.55 (0.46, 0.66) | 0.55 (0.46, 0.66) | 0.55 (0.46, 0.66) | 0.55 (0.46, 0.66) | 0.55 (0.46, 0.66) | 0.56 (0.47, 0.66) | 0.56 (0.47, 0.66) |
|  | Voluntary controlled | 0.59 (0.47, 0.75) | 0.59 (0.47, 0.75) | 0.59 (0.47, 0.75) | 0.59 (0.47, 0.75) | 0.59 (0.47, 0.75) | 0.59 (0.47, 0.75) | 0.58 (0.46, 0.73) | 0.58 (0.45, 0.73) |
| Missing EYSFSP score | Yes vs. no (ref.) |  |  |  |  |  |  |  |  |
| Standardised EYSFP score |  |  |  |  |  |  |  |  |  |
| _cons |  | 0.00 (0.00, 0.00) | 0.00 (0.00, 0.00) | 0.00 (0.00, 0.00) | 0.00 (0.00, 0.00) | 0.00 (0.00, 0.00) | 0.00 (0.00, 0.00) | 0.00 (0.00, 0.00) | 0.00 (0.00, 0.00) |
| LA-specific associations | |  |  |  |  |  |  |  |  |
| Pupil population | Linear term |  |  | 1.00 (1.00, 1.00) |  |  |  |  |  |
|  | Quadratic term |  |  | 1.00 (1.00, 1.00) |  |  |  |  |  |
| Special school attendance | Linear term |  |  |  | 1.18 (0.55, 2.51) |  |  |  |  |
|  | Quadratic term |  |  |  | 1.16 (0.74, 1.81) |  |  |  |  |
| Maintained school attendance | Linear term |  |  |  |  | 1.00 (1.00, 1.01) |  |  |  |
| Academy attendance | Linear term |  |  |  |  |  | 1.00 (0.99, 1.00) |  |  |
| FSM eligible | Linear term |  |  |  |  |  |  | 0.93 (0.89, 0.96) |  |
|  | Quadratic term |  |  |  |  |  |  | 1.00 (1.00, 1.00) |  |
| IDACI mode groups | 1 Most deprived 20% |  |  |  |  |  |  |  | 0.67 (0.54, 0.84) |
|  | 2 |  |  |  |  |  |  |  | 0.84 (0.66, 1.08) |
|  | 3 |  |  |  |  |  |  |  | 0.73 (0.55, 0.96) |
|  | 4 |  |  |  |  |  |  |  | 0.98 (0.74, 1.29) |
|  | 5 Least deprived 20% |  |  |  |  |  |  |  | Reference |
| LA-general effects |  |  |  |  |  |  |  |  |  |
| LA variance (95% CI) |  |  | 0.15 (0.09, 0.23) | 0.14 (0.09, 0.22) | 0.14 (0.09, 0.22) | 0.15 (0.09, 0.23) | 0.15 (0.09, 0.23) | 0.12 (0.07, 0.19) | 0.12 (0.07, 0.19) |
| PCV* |  |  |  | 3.25% | 4.11% | -0.08% | -0.11% | 17.60% | 19.06% |
| AUC (95% CI) |  | 0.75 (0.74, 0.76) | 0.78 (0.77, 0.79) | 0.78 (0.77, 0.79) | 0.78 (0.77, 0.79) | 0.78 (0.77, 0.79) | 0.78 (0.77, 0.79) | 0.78 (0.76, 0.79) | 0.78 (0.76, 0.79) |
| AUC change* |  |  | 0.02 | 0.00 | 0.00 | 0.00 | 0.00 | 0.00 | 0.00 |
| ICC % (95% CI) |  |  | 4.22 (2.76, 6.41) | 4.09 (2.66, 6.25) | 4.06 (2.62, 6.25) | 4.23 (2.77, 6.41) | 4.23 (2.77, 6.41) | 3.51 (2.21, 5.53) | 3.45 (2.15, 5.48) |
| Model parameters/goodness of fit |  |  |  |  |  |  |  |  |  |
| N |  | 18983 | 18983 | 18983 | 18983 | 18983 | 18983 | 18983 | 18983 |
| Log likelihood |  | -5581.2 | -5545.3 | -5544.3 | -5539.3 | -5545.2 | -5545.2 | -5536.0 | -5537.5 |
| Degrees of freedom |  | 43 | 44 | 46 | 46 | 45 | 45 | 46 | 48 |
| AIC |  | 11248.4 | 11178.6 | 11180.7 | 11170.6 | 11180.4 | 11180.4 | 11164.0 | 11171.1 |
| AIC change* |  |  | -69.8 | 2.1 | -8.0 | 1.8 | 1.8 | -14.6 | -7.5 |
| BIC |  | 11586.0 | 11524.0 | 11541.8 | 11531.8 | 11533.7 | 11533.7 | 11525.2 | 11548.0 |
| BIC change* |  |  | -62.0 | 17.8 | 7.8 | 9.7 | 9.7 | 1.2 | 24.0 |
| **(c) EHCP vs. no SEND provision** |  | **Step one (single level** | **Step two (multilevel)** | **Step three (multilevel, + pupil headcount)** | **Step three (multilevel, + special school attendance %)** | **Step three (multilevel, + maintained school attendance %)** | **Step three (multilevel, + academy school attendance %)** | **Step three (multilevel, + FSM eligible %)** | **Step three (multilevel, + IDACI mode)** |
|  |  | **cOR (95% CI)** | **cOR (95% CI)** | **cOR (95% CI)** | **cOR (95% CI)** | **cOR (95% CI)** | **cOR (95% CI)** | **cOR (95% CI)** | **cOR (95% CI)** |
| *Child-specific associations* |  |  |  |  |  |  |  |  |  |
| Year of birth (1 September to 31 August) | 2003/04 | Reference | Reference | Reference | Reference | Reference | Reference | Reference | Reference |
|  | 2004/05 | 0.90 (0.70, 1.17) | 0.90 (0.69, 1.17) | 0.90 (0.70, 1.17) | 0.89 (0.69, 1.16) | 0.90 (0.69, 1.17) | 0.90 (0.69, 1.17) | 0.93 (0.72, 1.20) | 0.90 (0.70, 1.17) |
|  | 2005/06 | 1.06 (0.82, 1.36) | 1.06 (0.83, 1.37) | 1.07 (0.83, 1.37) | 1.06 (0.82, 1.36) | 1.07 (0.83, 1.38) | 1.07 (0.83, 1.38) | 1.11 (0.86, 1.43) | 1.07 (0.83, 1.38) |
|  | 2006/07 | 0.92 (0.71, 1.20) | 0.92 (0.71, 1.20) | 0.92 (0.71, 1.20) | 0.91 (0.70, 1.19) | 0.94 (0.72, 1.22) | 0.94 (0.72, 1.22) | 0.95 (0.74, 1.24) | 0.93 (0.71, 1.20) |
|  | 2007/08 | 1.00 (0.78, 1.27) | 1.00 (0.78, 1.28) | 1.00 (0.78, 1.28) | 0.95 (0.75, 1.22) | 1.02 (0.80, 1.31) | 1.02 (0.80, 1.32) | 1.01 (0.79, 1.29) | 1.01 (0.79, 1.29) |
|  | 2008/09 | 0.99 (0.78, 1.25) | 0.99 (0.78, 1.25) | 0.99 (0.78, 1.25) | 0.92 (0.73, 1.17) | 1.02 (0.80, 1.31) | 1.02 (0.80, 1.31) | 0.97 (0.77, 1.23) | 1.00 (0.79, 1.26) |
|  | 2009/10 | 0.85 (0.68, 1.08) | 0.85 (0.67, 1.08) | 0.85 (0.67, 1.08) | 0.78 (0.62, 1.00) | 0.89 (0.69, 1.14) | 0.89 (0.69, 1.14) | 0.78 (0.61, 0.99) | 0.86 (0.68, 1.09) |
|  | 2010/11 | 0.93 (0.74, 1.17) | 0.93 (0.74, 1.17) | 0.93 (0.73, 1.17) | 0.84 (0.67, 1.07) | 0.98 (0.76, 1.26) | 0.98 (0.76, 1.26) | 0.84 (0.66, 1.06) | 0.93 (0.74, 1.18) |
|  | 2011/12 | 1.05 (0.84, 1.32) | 1.06 (0.84, 1.33) | 1.05 (0.83, 1.33) | 0.93 (0.73, 1.18) | 1.12 (0.86, 1.46) | 1.12 (0.87, 1.46) | 0.95 (0.75, 1.20) | 1.07 (0.85, 1.34) |
|  | 2012/13 | 1.14 (0.91, 1.43) | 1.15 (0.92, 1.45) | 1.15 (0.91, 1.44) | 1.01 (0.79, 1.28) | 1.23 (0.94, 1.61) | 1.24 (0.95, 1.61) | 1.12 (0.89, 1.41) | 1.16 (0.93, 1.46) |
| Maternal age | Linear term | 0.94 (0.88, 1.00) | 0.93 (0.87, 1.00) | 0.93 (0.87, 1.00) | 0.93 (0.87, 1.00) | 0.93 (0.87, 1.00) | 0.93 (0.87, 1.00) | 0.93 (0.87, 1.00) | 0.93 (0.87, 1.00) |
|  | Quadratic term | 1.00 (1.00, 1.00) | 1.00 (1.00, 1.00) | 1.00 (1.00, 1.00) | 1.00 (1.00, 1.00) | 1.00 (1.00, 1.00) | 1.00 (1.00, 1.00) | 1.00 (1.00, 1.00) | 1.00 (1.00, 1.00) |
|  | Missing | 0.24 (0.07, 0.84) | 0.21 (0.06, 0.75) | 0.21 (0.06, 0.75) | 0.21 (0.06, 0.76) | 0.21 (0.06, 0.76) | 0.21 (0.06, 0.76) | 0.20 (0.06, 0.73) | 0.21 (0.06, 0.74) |
| Gender | Male vs. female (ref.) | 3.16 (2.83, 3.52) | 3.17 (2.84, 3.54) | 3.17 (2.84, 3.54) | 3.18 (2.85, 3.55) | 3.17 (2.84, 3.54) | 3.17 (2.84, 3.54) | 3.17 (2.84, 3.54) | 3.17 (2.84, 3.54) |
| Chronic condition (cancer/blood) | Yes vs. no (ref.) | 0.86 (0.59, 1.26) | 0.85 (0.58, 1.25) | 0.86 (0.59, 1.25) | 0.86 (0.59, 1.25) | 0.85 (0.58, 1.25) | 0.85 (0.58, 1.25) | 0.84 (0.58, 1.23) | 0.85 (0.58, 1.25) |
| Chronic condition (cardiovascular) | Yes vs. no (ref.) | 3.10 (2.32, 4.14) | 3.10 (2.31, 4.15) | 3.10 (2.32, 4.16) | 3.11 (2.32, 4.17) | 3.11 (2.32, 4.16) | 3.11 (2.32, 4.16) | 3.10 (2.32, 4.16) | 3.12 (2.33, 4.18) |
| Chronic condition (chronic infection) | Yes vs. no (ref.) | 1.07 (0.37, 3.07) | 1.24 (0.45, 3.45) | 1.24 (0.45, 3.45) | 1.29 (0.47, 3.56) | 1.23 (0.44, 3.43) | 1.23 (0.44, 3.42) | 1.23 (0.44, 3.43) | 1.23 (0.44, 3.44) |
| Chronic condition (mental health/developmental) | Yes vs. no (ref.) | 50.94 (41.84, 62.02) | 51.46 (42.15, 62.83) | 51.44 (42.14, 62.81) | 51.55 (42.22, 62.95) | 51.49 (42.18, 62.87) | 51.50 (42.18, 62.88) | 51.50 (42.18, 62.87) | 51.22 (41.95, 62.55) |
| Chronic condition (metabolic endocrine) | Yes vs. no (ref.) | 1.33 (1.10, 1.61) | 1.32 (1.09, 1.61) | 1.32 (1.09, 1.61) | 1.32 (1.09, 1.61) | 1.32 (1.09, 1.61) | 1.32 (1.09, 1.61) | 1.33 (1.09, 1.61) | 1.33 (1.09, 1.61) |
| Chronic condition (musculoskeletal skin) | Yes vs. no (ref.) | 2.37 (1.77, 3.17) | 2.39 (1.78, 3.20) | 2.39 (1.78, 3.20) | 2.39 (1.78, 3.20) | 2.39 (1.78, 3.20) | 2.39 (1.78, 3.20) | 2.40 (1.79, 3.21) | 2.39 (1.78, 3.20) |
| Chronic condition (neurological) | Yes vs. no (ref.) | 5.86 (5.10, 6.73) | 5.95 (5.18, 6.84) | 5.95 (5.18, 6.84) | 5.96 (5.19, 6.85) | 5.95 (5.18, 6.84) | 5.95 (5.18, 6.84) | 5.96 (5.19, 6.85) | 5.96 (5.19, 6.85) |
| Chronic condition (non-specific) | Yes vs. no (ref.) | 7.40 (6.10, 8.96) | 7.63 (6.29, 9.26) | 7.63 (6.29, 9.26) | 7.62 (6.27, 9.25) | 7.63 (6.28, 9.26) | 7.63 (6.28, 9.26) | 7.64 (6.29, 9.28) | 7.64 (6.29, 9.27) |
| Chronic condition (respiratory) | Yes vs. no (ref.) | 0.94 (0.76, 1.15) | 0.93 (0.75, 1.14) | 0.93 (0.75, 1.14) | 0.93 (0.75, 1.14) | 0.93 (0.75, 1.14) | 0.93 (0.75, 1.14) | 0.93 (0.75, 1.14) | 0.93 (0.75, 1.14) |
| IDACI | 1 Most deprived 20% | 1.11 (0.93, 1.32) | 1.24 (1.03, 1.49) | 1.24 (1.03, 1.49) | 1.22 (1.01, 1.46) | 1.24 (1.03, 1.49) | 1.24 (1.03, 1.49) | 1.34 (1.12, 1.62) | 1.35 (1.12, 1.63) |
|  | 2 | 1.25 (1.05, 1.48) | 1.31 (1.10, 1.55) | 1.31 (1.10, 1.55) | 1.30 (1.09, 1.54) | 1.31 (1.10, 1.56) | 1.31 (1.10, 1.56) | 1.36 (1.14, 1.62) | 1.36 (1.15, 1.62) |
|  | 3 | 1.06 (0.89, 1.27) | 1.10 (0.92, 1.32) | 1.10 (0.92, 1.32) | 1.11 (0.92, 1.32) | 1.11 (0.92, 1.32) | 1.11 (0.92, 1.32) | 1.12 (0.94, 1.34) | 1.13 (0.95, 1.36) |
|  | 4 | 1.00 (0.83, 1.20) | 1.01 (0.84, 1.21) | 1.01 (0.84, 1.21) | 1.01 (0.84, 1.22) | 1.01 (0.84, 1.21) | 1.01 (0.84, 1.21) | 1.02 (0.85, 1.23) | 1.02 (0.85, 1.23) |
|  | 5 Least deprived 20% | Reference | Reference | Reference | Reference | Reference | Reference | Reference | Reference |
|  | Missing | 16.49 (4.57, 59.53) | 19.60 (5.28, 72.81) | 19.43 (5.23, 72.14) | 20.70 (5.56, 77.02) | 19.64 (5.29, 72.98) | 19.65 (5.29, 73.02) | 22.00 (5.91, 81.87) | 22.22 (5.98, 82.61) |
| FSM eligible | Yes vs. no (ref.) | 2.01 (1.78, 2.27) | 2.02 (1.79, 2.29) | 2.02 (1.79, 2.28) | 2.02 (1.79, 2.28) | 2.02 (1.79, 2.29) | 2.02 (1.79, 2.29) | 2.05 (1.82, 2.32) | 2.04 (1.81, 2.31) |
| Racial-ethnic group | Asian | 1.16 (0.96, 1.40) | 1.18 (0.97, 1.44) | 1.18 (0.97, 1.44) | 1.17 (0.97, 1.43) | 1.18 (0.97, 1.43) | 1.18 (0.97, 1.43) | 1.20 (0.99, 1.46) | 1.21 (1.00, 1.47) |
|  | Black | 2.01 (1.66, 2.43) | 2.09 (1.71, 2.56) | 2.09 (1.71, 2.55) | 2.08 (1.70, 2.54) | 2.09 (1.71, 2.55) | 2.09 (1.71, 2.55) | 2.16 (1.77, 2.64) | 2.18 (1.78, 2.66) |
|  | Chinese | 1.67 (0.88, 3.14) | 1.71 (0.91, 3.23) | 1.71 (0.91, 3.23) | 1.71 (0.91, 3.23) | 1.71 (0.90, 3.23) | 1.71 (0.90, 3.22) | 1.74 (0.92, 3.29) | 1.74 (0.92, 3.29) |
|  | Mixed | 0.81 (0.65, 1.02) | 0.82 (0.65, 1.03) | 0.82 (0.65, 1.03) | 0.82 (0.65, 1.03) | 0.82 (0.65, 1.03) | 0.82 (0.65, 1.03) | 0.83 (0.66, 1.04) | 0.84 (0.66, 1.05) |
|  | Other | 1.09 (0.74, 1.62) | 1.09 (0.73, 1.62) | 1.09 (0.73, 1.61) | 1.08 (0.72, 1.60) | 1.09 (0.73, 1.62) | 1.09 (0.73, 1.61) | 1.12 (0.75, 1.67) | 1.15 (0.77, 1.70) |
|  | White | Reference | Reference | Reference | Reference | Reference | Reference | Reference | Reference |
|  | Missing | 1.11 (0.49, 2.48) | 1.13 (0.50, 2.54) | 1.13 (0.50, 2.54) | 1.13 (0.50, 2.55) | 1.13 (0.50, 2.54) | 1.13 (0.50, 2.54) | 1.13 (0.50, 2.54) | 1.14 (0.50, 2.56) |
| EAL | No | Reference | Reference | Reference | Reference | Reference | Reference | Reference | Reference |
|  | Yes | 1.07 (0.91, 1.26) | 1.05 (0.89, 1.24) | 1.05 (0.89, 1.24) | 1.05 (0.89, 1.24) | 1.05 (0.89, 1.24) | 1.05 (0.89, 1.24) | 1.05 (0.89, 1.24) | 1.07 (0.90, 1.26) |
|  | Unclear or missing | 0.86 (0.32, 2.28) | 0.90 (0.34, 2.40) | 0.90 (0.34, 2.39) | 0.90 (0.34, 2.41) | 0.90 (0.34, 2.40) | 0.90 (0.34, 2.40) | 0.90 (0.34, 2.41) | 0.89 (0.33, 2.40) |
| Rate of hospitalisation (pre year 1) |  | 1.01 (1.00, 1.02) | 1.01 (1.00, 1.02) | 1.01 (1.00, 1.02) | 1.01 (1.00, 1.02) | 1.01 (1.00, 1.02) | 1.01 (1.00, 1.02) | 1.01 (1.00, 1.02) | 1.01 (1.00, 1.02) |
| Age at Year One start |  | 0.80 (0.68, 0.95) | 0.80 (0.68, 0.94) | 0.80 (0.68, 0.94) | 0.80 (0.68, 0.95) | 0.80 (0.68, 0.94) | 0.80 (0.68, 0.94) | 0.80 (0.68, 0.94) | 0.80 (0.68, 0.94) |
| School governance | Community | Reference | Reference | Reference | Reference | Reference | Reference | Reference | Reference |
|  | Sponsor led academy | 0.66 (0.51, 0.85) | 0.65 (0.50, 0.84) | 0.65 (0.50, 0.84) | 0.66 (0.51, 0.85) | 0.66 (0.51, 0.86) | 0.66 (0.51, 0.86) | 0.64 (0.50, 0.83) | 0.64 (0.50, 0.83) |
|  | Converter led academy | 0.76 (0.65, 0.89) | 0.76 (0.65, 0.89) | 0.76 (0.65, 0.90) | 0.77 (0.65, 0.90) | 0.78 (0.66, 0.92) | 0.78 (0.66, 0.92) | 0.76 (0.64, 0.89) | 0.76 (0.64, 0.89) |
|  | Free school | 0.65 (0.33, 1.29) | 0.64 (0.32, 1.26) | 0.64 (0.33, 1.27) | 0.64 (0.33, 1.26) | 0.64 (0.33, 1.27) | 0.64 (0.33, 1.27) | 0.64 (0.32, 1.26) | 0.65 (0.33, 1.28) |
|  | Voluntary aided | 0.49 (0.42, 0.58) | 0.48 (0.41, 0.57) | 0.48 (0.41, 0.57) | 0.48 (0.41, 0.57) | 0.48 (0.41, 0.57) | 0.48 (0.41, 0.57) | 0.49 (0.41, 0.58) | 0.49 (0.41, 0.57) |
|  | Voluntary controlled | 0.49 (0.39, 0.62) | 0.49 (0.39, 0.61) | 0.49 (0.39, 0.61) | 0.49 (0.39, 0.62) | 0.49 (0.39, 0.61) | 0.49 (0.39, 0.61) | 0.48 (0.38, 0.60) | 0.47 (0.38, 0.60) |
| Missing EYSFSP score | Yes vs. no (ref.) |  |  |  |  |  |  |  |  |
| Standardised EYSFP score |  |  |  |  |  |  |  |  |  |
| _cons |  | 0.01 (0.00, 0.04) | 0.01 (0.00, 0.04) | 0.00 (0.00, 0.00) | 0.00 (0.00, 0.00) | 0.01 (0.00, 0.03) | 0.01 (0.00, 0.04) | 0.00 (0.00, 0.00) | 0.01 (0.00, 0.04) |
| *LA-specific associations* | |  |  |  |  |  |  |  |  |
| Pupil population | Linear term |  |  | 1.00 (1.00, 1.00) |  |  |  |  |  |
|  | Quadratic term |  |  | 1.00 (1.00, 1.00) |  |  |  |  |  |
| Special school attendance | Linear term |  |  |  | 1.09 (0.53, 2.26) |  |  |  |  |
|  | Quadratic term |  |  |  | 1.27 (0.83, 1.94) |  |  |  |  |
| Maintained school attendance | Linear term |  |  |  |  | 1.00 (1.00, 1.01) |  |  |  |
| Academy attendance | Linear term |  |  |  |  |  | 1.00 (0.99, 1.00) |  |  |
| FSM eligible | Linear term |  |  |  |  |  |  | 0.93 (0.90, 0.97) |  |
|  | Quadratic term |  |  |  |  |  |  | 1.00 (1.00, 1.00) |  |
| IDACI mode groups | 1 Most deprived 20% |  |  |  |  |  |  |  | 0.64 (0.52, 0.78) |
|  | 2 |  |  |  |  |  |  |  | 0.86 (0.68, 1.08) |
|  | 3 |  |  |  |  |  |  |  | 0.70 (0.54, 0.92) |
|  | 4 |  |  |  |  |  |  |  | 0.96 (0.74, 1.24) |
|  | 5 Least deprived 20% |  |  |  |  |  |  |  | Reference |
| *LA-general effects* |  |  |  |  |  |  |  |  |  |
| LA variance (95% CI) |  |  | 0.13 (0.08, 0.21) | 0.13 (0.08, 0.20) | 0.12 (0.08, 0.20) | 0.13 (0.08, 0.21) | 0.13 (0.08, 0.21) | 0.10 (0.06, 0.17) | 0.10 (0.06, 0.17) |
| PCV* |  |  |  | 2.16% | 3.61% | 0.07% | 0.05% | 21.89% | 23.82% |
| AUC (95% CI) |  | 0.82 (0.81, 0.83) | 0.84 (0.83, 0.85) | 0.84 (0.83, 0.85) | 0.84 (0.83, 0.85) | 0.84 (0.83, 0.85) | 0.84 (0.83, 0.85) | 0.84 (0.83, 0.85) | 0.84 (0.83, 0.85) |
| AUC change* |  |  | 0.02 | 0.00 | 0.00 | 0.00 | 0.00 | 0.00 | 0.00 |
| ICC % (95% CI) |  |  | 3.79 (2.42, 5.88) | 3.71 (2.36, 5.80) | 3.66 (2.28, 5.81) | 3.79 (2.42, 5.88) | 3.79 (2.42, 5.88) | 2.98 (1.80, 4.91) | 2.91 (1.75, 4.82) |
| *Model parameters/goodness of fit* |  |  |  |  |  |  |  |  |  |
| N |  | 126093 | 126093 | 126093 | 126093 | 126093 | 126093 | 126093 | 126093 |
| Log likelihood |  | -8015.6 | -7982.8 | -7982.5 | -7973.5 | -7982.4 | -7982.3 | -7972.4 | -7971.4 |
| Degrees of freedom |  | 44 | 45 | 47 | 47 | 46 | 46 | 47 | 49 |
| AIC |  | 16119.2 | 16055.6 | 16059.0 | 16041.0 | 16056.7 | 16056.6 | 16038.9 | 16040.8 |
| AIC change* |  |  | -63.6 | 4880.4 | 4862.4 | 4878.1 | 4878.0 | 4860.3 | 4862.2 |
| BIC |  | 16547.9 | 16494.1 | 16517.0 | 16499.0 | 16505.0 | 16504.8 | 16496.9 | 16518.3 |
| BIC change* |  |  | -53.8 | 4993.0 | 4975.0 | 4981.0 | 4980.8 | 4972.9 | 4994.3 |

AIC= Akaike information criterion; AUC = area under the receiving operator characteristic (ROC) curve; BIC = and Bayesian information criterion; CI = confidence interval; cOR = condition odds ratio; EAL = English as an additional language; EHCP = education, health and care plan; EYFSP = Early years foundation stage profile; FSM = free school meals; IDACI = Income deprivation affecting children index; IQR = interquartile range; LA = local authority; PCV=percentage change in variance; SEND=special educational needs and disability; *change in relation to the previous step (i.e. step 1 for step 2 model, step 2 model for step 3 models); Positive values for AUC change and PCV indicate increases in the discimination and variance explained compared with model from the previous step. Negative AIC/BIC change values indicates better model fit.


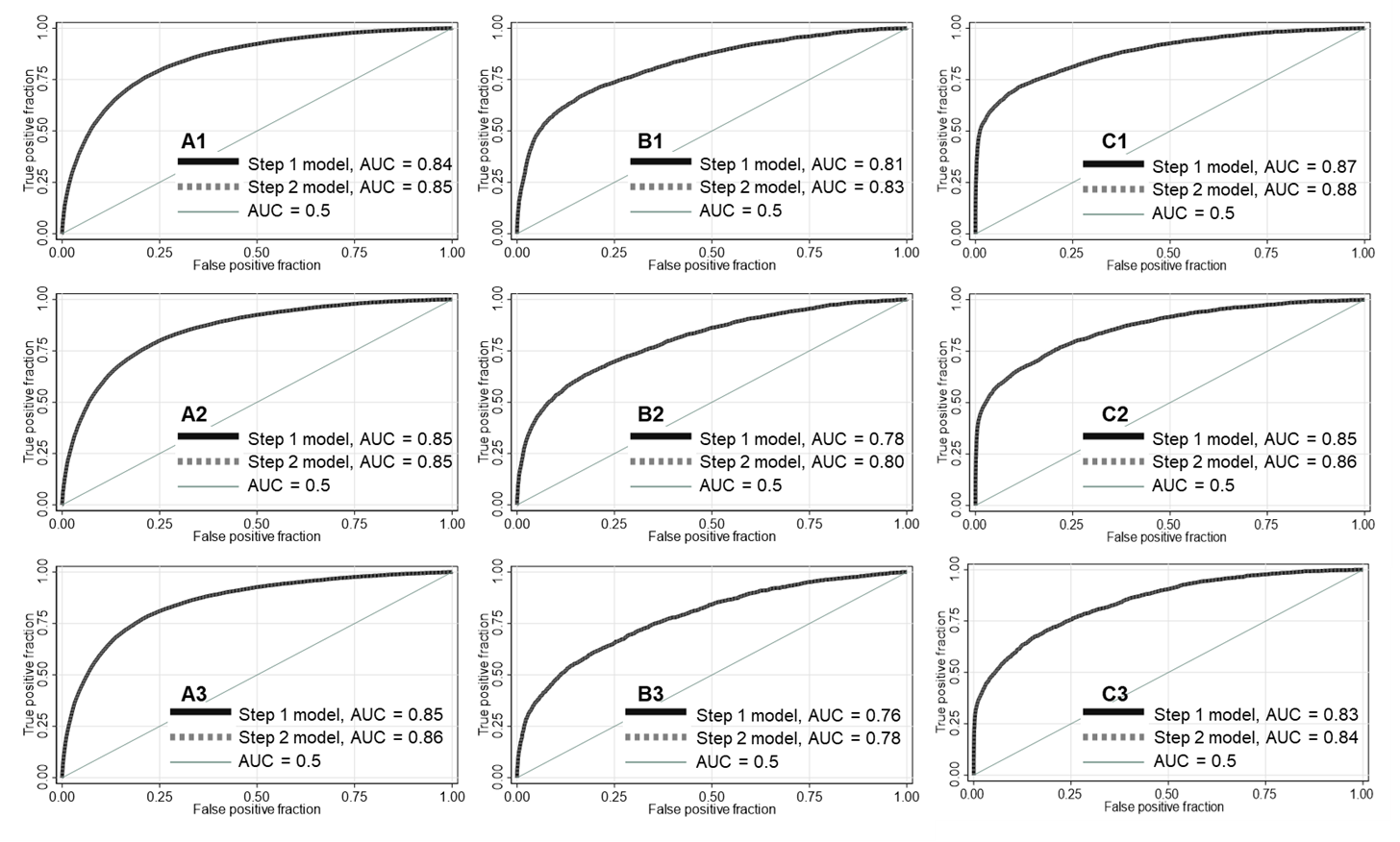


**Figure S5.** Receiving operator characteristic curves for step 1 (single level) and step 2 (multilevel) logistic regression models of (A) SEND support vs. no SEND provision, (B) EHCP vs. SEND support and (C) EHCP vs. no SEND provision: stratified by gestational age groups (1) late preterm, (2) early term, and (3) full term. AUC = area under the receiving operator characteristic curve; EHCP = education, health and care plan; SEND = special educational needs and disability

**Table S7.** LA-general effects: SEND support vs. no SEND provision – main and sensitivity analyses combined

|  | **Step one (single level** | **Step two (multilevel)** | **Step three (multilevel, + pupil headcount)** | **Step three (multilevel, + special school attendance %)** | **Step three (multilevel, + maintained school attendance %)** | **Step three (multilevel, + academy school attendance %)** | **Step three (multilevel, + FSM eligible %)** | **Step three (multilevel, + IDACI mode)** |
| --- | --- | --- | --- | --- | --- | --- | --- | --- |
| *Late preterm: SEND support vs. no SEND provision* |  |  |  |  |  |  |  |  |
| **Main analysis** |  |  |  |  |  |  |  |  |
| LA variance (95% CI) |  | 0.08 (0.06, 0.10) | 0.08 (0.06, 0.10) | 0.08 (0.06, 0.10) | 0.08 (0.06, 0.10) | 0.08 (0.06, 0.10) | 0.07 (0.06, 0.10) | 0.08 (0.06, 0.11) |
| PCV* |  |  | -0.90% | 0.31% | 0.77% | 1.13% | 1.75% | -5.26% |
| AUC (95% CI) | 0.84 (0.84, 0.85) | 0.85 (0.85, 0.85) | 0.85 (0.85, 0.85) | 0.85 (0.85, 0.85) | 0.85 (0.85, 0.85) | 0.85 (0.85, 0.85) | 0.85 (0.85, 0.85) | 0.85 (0.85, 0.85) |
| AUC change* |  | 0.004 | 0.000 | 0.000 | 0.000 | 0.000 | 0.000 | 0.000 |
| ICC % (95% CI) |  | 2.26 (1.73, 2.95) | 2.28 (1.74, 2.98) | 2.26 (1.72, 2.95) | 2.24 (1.72, 2.93) | 2.24 (1.71, 2.92) | 2.22 (1.70, 2.90) | 2.38 (1.81, 3.12) |
| **Sensitivity 1: 2008/09-2012/13 births** |  |  |  |  |  |  |  |  |
| LA variance (95% CI) |  | 0.09 (0.07, 0.12) | 0.09 (0.07, 0.12) | 0.09 (0.07, 0.12) | 0.09 (0.06, 0.12) | 0.09 (0.06, 0.12) | 0.09 (0.07, 0.12) | 0.09 (0.07, 0.12) |
| PCV* |  |  | 0.79% | -0.49% | 2.89% | 3.48% | 0.80% | -1.13% |
| AUC (95% CI) | 0.85 (0.85, 0.85) | 0.85 (0.85, 0.86) | 0.85 (0.85, 0.86) | 0.85 (0.85, 0.86) | 0.85 (0.85, 0.86) | 0.85 (0.85, 0.86) | 0.85 (0.85, 0.86) | 0.85 (0.85, 0.86) |
| AUC change* |  | 0.005 | 0.000 | 0.000 | 0.000 | 0.000 | 0.000 | 0.000 |
| ICC % (95% CI) |  | 2.63 (1.97, 3.51) | 2.61 (1.95, 3.50) | 2.65 (1.98, 3.54) | 2.56 (1.91, 3.42) | 2.54 (1.90, 3.40) | 2.61 (1.95, 3.49) | 2.66 (1.98, 3.56) |
| **Sensitivity 2: Minimal adjustment set** |  |  |  |  |  |  |  |  |
| LA variance (95% CI) |  | 0.03 (0.02, 0.04) | 0.03 (0.02, 0.04) | 0.03 (0.02, 0.04) | 0.03 (0.02, 0.04) | 0.03 (0.02, 0.04) | 0.02 (0.01, 0.03) | 0.02 (0.02, 0.03) |
| PCV* |  |  | -0.10% | 5.54% | -0.80% | -0.65% | 40.28% | 31.65% |
| AUC (95% CI) | 0.57 (0.57, 0.58) | 0.59 (0.59, 0.60) | 0.59 (0.59, 0.60) | 0.59 (0.59, 0.60) | 0.59 (0.59, 0.60) | 0.59 (0.59, 0.60) | 0.59 (0.59, 0.60) | 0.59 (0.59, 0.60) |
| AUC change* |  | 0.020 | 0.000 | 0.000 | 0.000 | 0.000 | 0.000 | 0.000 |
| ICC % (95% CI) |  | 0.97 (0.72, 1.32) | 0.98 (0.72, 1.32) | 0.92 (0.67, 1.26) | 0.98 (0.72, 1.33) | 0.98 (0.72, 1.33) | 0.58 (0.42, 0.82) | 0.67 (0.48, 0.94) |
| *Early term: SEND support vs. no SEND provision* |  |  |  |  |  |  |  |  |
| **Main analysis** |  | 0.07 (0.06, 0.10) | 0.08 (0.06, 0.10) | 0.07 (0.05, 0.09) | 0.07 (0.05, 0.10) | 0.07 (0.05, 0.10) | 0.07 (0.05, 0.09) | 0.07 (0.05, 0.10) |
| LA variance (95% CI) |  |  | -2.91% | 1.84% | 1.18% | 1.57% | 2.60% | 1.37% |
| PCV* | 0.85 (0.85, 0.85) | 0.85 (0.85, 0.86) | 0.85 (0.85, 0.86) | 0.85 (0.85, 0.86) | 0.85 (0.85, 0.86) | 0.85 (0.85, 0.86) | 0.85 (0.85, 0.86) | 0.85 (0.85, 0.86) |
| AUC (95% CI) |  | 0.004 | 0.000 | 0.000 | 0.000 | 0.000 | 0.000 | 0.000 |
| AUC change* |  | 2.17 (1.65, 2.85) | 2.23 (1.70, 2.94) | 2.13 (1.62, 2.80) | 2.15 (1.63, 2.82) | 2.14 (1.63, 2.81) | 2.12 (1.61, 2.78) | 2.14 (1.63, 2.82) |
| ICC % (95% CI) |  | 0.07 (0.06, 0.10) | 0.08 (0.06, 0.10) | 0.07 (0.05, 0.09) | 0.07 (0.05, 0.10) | 0.07 (0.05, 0.10) | 0.07 (0.05, 0.09) | 0.07 (0.05, 0.10) |
| **Sensitivity 1: 2008/09-2012/13 births** |  |  |  |  |  |  |  |  |
| LA variance (95% CI) |  | 0.09 (0.06, 0.12) | 0.09 (0.06, 0.12) | 0.09 (0.06, 0.12) | 0.09 (0.06, 0.12) | 0.09 (0.06, 0.12) | 0.09 (0.06, 0.12) | 0.09 (0.06, 0.12) |
| PCV* |  |  | -0.30% | 0.53% | 1.55% | 2.08% | 0.95% | 0.30% |
| AUC (95% CI) | 0.85 (0.85, 0.86) | 0.86 (0.85, 0.86) | 0.86 (0.85, 0.86) | 0.86 (0.85, 0.86) | 0.86 (0.85, 0.86) | 0.86 (0.85, 0.86) | 0.86 (0.85, 0.86) | 0.86 (0.85, 0.86) |
| AUC change* |  | 0.005 | 0.000 | 0.000 | 0.000 | 0.000 | 0.000 | 0.000 |
| ICC % (95% CI) |  | 2.60 (1.93, 3.50) | 2.61 (1.93, 3.52) | 2.59 (1.92, 3.49) | 2.57 (1.90, 3.46) | 2.55 (1.89, 3.44) | 2.58 (1.91, 3.48) | 2.60 (1.93, 3.49) |
| **Sensitivity 2: Minimal adjustment set** |  |  |  |  |  |  |  |  |
| LA variance (95% CI) |  | 0.03 (0.02, 0.04) | 0.03 (0.02, 0.04) | 0.03 (0.02, 0.04) | 0.03 (0.02, 0.04) | 0.03 (0.02, 0.04) | 0.02 (0.01, 0.03) | 0.02 (0.02, 0.03) |
| PCV* |  |  | -3.35% | 3.82% | 0.05% | 0.10% | 32.75% | 28.60% |
| AUC (95% CI) | 0.58 (0.57, 0.58) | 0.60 (0.59, 0.60) | 0.60 (0.59, 0.60) | 0.60 (0.59, 0.60) | 0.60 (0.59, 0.60) | 0.60 (0.59, 0.60) | 0.60 (0.59, 0.60) | 0.59 (0.59, 0.60) |
| AUC change* |  | 0.019 | 0.000 | 0.000 | 0.000 | 0.000 | 0.000 | 0.000 |
| ICC % (95% CI) |  | 0.92 (0.68, 1.25) | 0.96 (0.70, 1.30) | 0.89 (0.65, 1.21) | 0.92 (0.68, 1.25) | 0.92 (0.68, 1.25) | 0.62 (0.44, 0.88) | 0.66 (0.47, 0.92) |
| **Sensitivity 3: Different random sample** |  |  |  |  |  |  |  |  |
| LA variance (95% CI) |  | 0.08 (0.06, 0.10) | 0.09 (0.07, 0.11) | 0.08 (0.06, 0.11) | 0.08 (0.06, 0.10) | 0.08 (0.06, 0.10) | 0.08 (0.06, 0.10) | 0.08 (0.06, 0.11) |
| PCV* |  |  | -8.01% | -0.11% | 1.61% | 2.02% | 0.88% | -1.57% |
| AUC (95% CI) | 0.85 (0.85, 0.85) | 0.85 (0.85, 0.86) | 0.85 (0.85, 0.86) | 0.85 (0.85, 0.86) | 0.85 (0.85, 0.86) | 0.85 (0.85, 0.86) | 0.85 (0.85, 0.86) | 0.85 (0.85, 0.86) |
| AUC change* |  | 0.004 | 0.000 | 0.000 | 0.000 | 0.000 | 0.000 | 0.000 |
| ICC % (95% CI) |  | 2.36 (1.80, 3.09) | 2.54 (1.94, 3.33) | 2.36 (1.80, 3.09) | 2.32 (1.77, 3.04) | 2.31 (1.76, 3.03) | 2.34 (1.78, 3.07) | 2.40 (1.82, 3.14) |
| *Full-term: SEND support vs. no SEND provision* |  |  |  |  |  |  |  |  |
| **Main analysis** |  |  |  |  |  |  |  |  |
| LA variance (95% CI) |  | 0.07 (0.05, 0.09) | 0.07 (0.05, 0.09) | 0.07 (0.05, 0.09) | 0.07 (0.05, 0.09) | 0.07 (0.05, 0.09) | 0.07 (0.05, 0.09) | 0.07 (0.05, 0.09) |
| PCV* |  |  | -2.27% | 2.05% | 0.81% | 1.54% | 1.03% | -0.92% |
| AUC (95% CI) | 0.86 (0.85, 0.86) | 0.86 (0.86, 0.86) | 0.86 (0.86, 0.86) | 0.86 (0.86, 0.86) | 0.86 (0.86, 0.86) | 0.86 (0.86, 0.86) | 0.86 (0.86, 0.86) | 0.86 (0.86, 0.86) |
| AUC change* |  | 0.004 | 0.000 | 0.000 | 0.000 | 0.000 | 0.000 | 0.000 |
| ICC % (95% CI) |  | 2.03 (1.53, 2.70) | 2.08 (1.56, 2.76) | 1.99 (1.50, 2.65) | 2.02 (1.52, 2.68) | 2.00 (1.51, 2.66) | 2.01 (1.51, 2.68) | 2.05 (1.54, 2.73) |
| **Sensitivity 1: 2008/09-2012/13 births** |  |  |  |  |  |  |  |  |
| LA variance (95% CI) |  | 0.09 (0.06, 0.12) | 0.09 (0.06, 0.12) | 0.09 (0.06, 0.12) | 0.09 (0.06, 0.12) | 0.09 (0.06, 0.12) | 0.09 (0.06, 0.12) | 0.09 (0.06, 0.12) |
| PCV* |  |  | -0.30% | 0.53% | 1.55% | 2.08% | 0.95% | 0.30% |
| AUC (95% CI) | 0.85 (0.85, 0.86) | 0.86 (0.85, 0.86) | 0.86 (0.85, 0.86) | 0.86 (0.85, 0.86) | 0.86 (0.85, 0.86) | 0.86 (0.85, 0.86) | 0.86 (0.85, 0.86) | 0.86 (0.85, 0.86) |
| AUC change* |  | 0.005 | 0.000 | 0.000 | 0.000 | 0.000 | 0.000 | 0.000 |
| ICC % (95% CI) |  | 2.60 (1.93, 3.50) | 2.61 (1.93, 3.52) | 2.59 (1.92, 3.49) | 2.57 (1.90, 3.46) | 2.55 (1.89, 3.44) | 2.58 (1.91, 3.48) | 2.60 (1.93, 3.49) |
| **Sensitivity 2: Minimal adjustment set** |  |  |  |  |  |  |  |  |
| LA variance (95% CI) |  | 0.03 (0.02, 0.05) | 0.03 (0.02, 0.05) | 0.03 (0.02, 0.04) | 0.03 (0.02, 0.05) | 0.03 (0.02, 0.05) | 0.02 (0.02, 0.03) | 0.02 (0.02, 0.03) |
| PCV* |  |  | 0.63% | 7.65% | 0.24% | 1.05% | 34.50% | 30.13% |
| AUC (95% CI) | 0.59 (0.59, 0.59) | 0.61 (0.60, 0.61) | 0.61 (0.60, 0.61) | 0.61 (0.60, 0.61) | 0.61 (0.60, 0.61) | 0.61 (0.60, 0.61) | 0.61 (0.60, 0.61) | 0.61 (0.60, 0.61) |
| AUC change* |  | 0.018 | 0.000 | 0.000 | 0.000 | 0.000 | 0.000 | 0.000 |
| ICC % (95% CI) |  | 1.01 (0.74, 1.38) | 1.00 (0.73, 1.37) | 0.93 (0.67, 1.28) | 1.00 (0.73, 1.38) | 1.00 (0.73, 1.37) | 0.66 (0.46, 0.95) | 0.71 (0.49, 1.01) |
| **Sensitivity 3: Different random sample** |  |  |  |  |  |  |  |  |
| LA variance (95% CI) |  | 0.07 (0.06, 0.10) | 0.08 (0.06, 0.10) | 0.07 (0.05, 0.10) | 0.07 (0.06, 0.10) | 0.07 (0.06, 0.10) | 0.07 (0.05, 0.10) | 0.08 (0.06, 0.10) |
| PCV* |  |  | -1.27% | 1.23% | -0.33% | -0.09% | 2.52% | -1.88% |
| AUC (95% CI) | 0.85 (0.85, 0.86) | 0.86 (0.85, 0.86) | 0.86 (0.85, 0.86) | 0.86 (0.85, 0.86) | 0.86 (0.85, 0.86) | 0.86 (0.85, 0.86) | 0.86 (0.85, 0.86) | 0.86 (0.85, 0.86) |
| AUC change* |  | 0.004 | 0.000 | 0.000 | 0.000 | 0.000 | 0.000 | 0.000 |
| ICC % (95% CI) |  | 2.20 (1.66, 2.92) | 2.23 (1.68, 2.96) | 2.18 (1.64, 2.89) | 2.21 (1.67, 2.93) | 2.21 (1.66, 2.92) | 2.15 (1.62, 2.85) | 2.24 (1.68, 2.98) |

AUC = area under the receiving operator characteristic curve; CI = confidence interval; ICC = intraclass correlation coefficients; LA = local authority; SEND = special educational needs and disability; PCV=percentage change in variance; SEND=special educational needs and disability; *change in relation to the previous step (i.e. step 1 for step 2 model, step 2 model for step 3 models); Positive values for AUC change and PCV indicate increases in the discrimination and variance explained compared with model from the previous step

**Table S8.** LA-general effects: EHCP vs. SEND provision – main and sensitivity analyses combined

|  | **Step one (multilevel)** | **Step two (multilevel)** | **Step three (multilevel, + pupil headcount)** | **Step three (multilevel, + special school attendance %)** | **Step three (multilevel, + maintained school attendance %)** | **Step three (multilevel, + academy school attendance %)** | **Step three (multilevel, + FSM eligible %)** | **Step three (multilevel, + IDACI mode)** |
| --- | --- | --- | --- | --- | --- | --- | --- | --- |
| *Late preterm: EHCP vs. SEND support* |  |  |  |  |  |  |  |  |
| **Main analysis** |  |  |  |  |  |  |  |  |
| LA variance (95% CI) |  | 0.20 (0.15, 0.28) | 0.20 (0.14, 0.28) | 0.17 (0.12, 0.24) | 0.20 (0.15, 0.28) | 0.20 (0.15, 0.28) | 0.19 (0.14, 0.27) | 0.18 (0.12, 0.25) |
| PCV* |  |  | 3.38% | 17.81% | 0.48% | 0.27% | 5.30% | 14.11% |
| AUC (95% CI) | 0.81 (0.80, 0.81) | 0.83 (0.82, 0.83) | 0.83 (0.82, 0.83) | 0.83 (0.82, 0.83) | 0.83 (0.82, 0.83) | 0.83 (0.82, 0.83) | 0.83 (0.82, 0.83) | 0.83 (0.82, 0.83) |
| AUC change* |  | 0.02 | 0.00 | 0.00 | 0.00 | 0.00 | 0.00 | 0.00 |
| ICC % (95% CI) |  | 5.84 (4.26, 7.97) | 5.66 (4.11, 7.74) | 4.85 (3.45, 6.79) | 5.82 (4.24, 7.94) | 5.83 (4.25, 7.95) | 5.55 (4.00, 7.65) | 5.06 (3.63, 7.02) |
| **Sensitivity 1: 2008/09-2012/13 births** |  |  |  |  |  |  |  |  |
| LA variance (95% CI) |  | 0.22 (0.15, 0.32) | 0.21 (0.14, 0.30) | 0.19 (0.13, 0.28) | 0.22 (0.15, 0.32) | 0.22 (0.15, 0.32) | 0.20 (0.14, 0.30) | 0.19 (0.13, 0.28) |
| PCV* |  |  | 6.87% | 14.07% | 0.49% | 0.30% | 7.86% | 14.58% |
| AUC (95% CI) | 0.80 (0.78, 0.81) | 0.82 (0.81, 0.83) | 0.82 (0.81, 0.83) | 0.82 (0.81, 0.83) | 0.82 (0.81, 0.83) | 0.82 (0.81, 0.83) | 0.82 (0.81, 0.83) | 0.82 (0.81, 0.83) |
| AUC change* |  | 0.022 | 0.000 | -0.001 | 0.000 | 0.000 | 0.000 | -0.002 |
| ICC % (95% CI) |  | 6.31 (4.48, 8.83) | 5.90 (4.14, 8.35) | 5.47 (3.79, 7.84) | 6.28 (4.45, 8.80) | 6.30 (4.46, 8.82) | 5.85 (4.09, 8.29) | 5.44 (3.77, 7.79) |
| **Sensitivity 2: Minimal adjustment set** |  |  |  |  |  |  |  |  |
| LA variance (95% CI) |  | 0.09 (0.06, 0.13) | 0.09 (0.06, 0.13) | 0.07 (0.05, 0.11) | 0.09 (0.06, 0.13) | 0.09 (0.06, 0.13) | 0.08 (0.05, 0.12) | 0.07 (0.05, 0.11) |
| PCV* |  |  | 1.16% | 16.73% | 0.24% | 0.13% | 10.18% | 18.95% |
| AUC (95% CI) | 0.59 (0.59, 0.60) | 0.63 (0.62, 0.64) | 0.63 (0.62, 0.64) | 0.63 (0.62, 0.64) | 0.63 (0.62, 0.64) | 0.63 (0.62, 0.64) | 0.63 (0.62, 0.64) | 0.63 (0.62, 0.64) |
| AUC change* |  | 0.038 | 0.000 | 0.000 | 0.000 | 0.000 | 0.000 | -0.001 |
| ICC % (95% CI) |  | 2.63 (1.81, 3.81) | 2.60 (1.79, 3.77) | 2.20 (1.47, 3.29) | 2.63 (1.81, 3.80) | 2.63 (1.81, 3.81) | 2.37 (1.59, 3.51) | 2.14 (1.43, 3.21) |
| *Early term: EHCP vs. SEND support* |  |  |  |  |  |  |  |  |
| **Main analysis** |  |  |  |  |  |  |  |  |
| LA variance (95% CI) |  | 0.17 (0.11, 0.25) | 0.16 (0.11, 0.24) | 0.14 (0.09, 0.21) | 0.17 (0.11, 0.25) | 0.17 (0.12, 0.25) | 0.17 (0.11, 0.24) | 0.14 (0.09, 0.21) |
| PCV* |  |  | 3.75% | 16.26% | -0.20% | -0.31% | 1.69% | 17.19% |
| AUC (95% CI) | 0.78 (0.77, 0.79) | 0.80 (0.79, 0.81) | 0.80 (0.79, 0.81) | 0.80 (0.79, 0.81) | 0.80 (0.79, 0.81) | 0.80 (0.79, 0.81) | 0.80 (0.79, 0.81) | 0.80 (0.79, 0.81) |
| AUC change* |  | 0.02 | 0.00 | 0.00 | 0.00 | 0.00 | 0.00 | 0.00 |
| ICC % (95% CI) |  | 4.86 (3.37, 6.97) | 4.69 (3.22, 6.77) | 4.10 (2.76, 6.07) | 4.87 (3.38, 6.98) | 4.88 (3.38, 6.99) | 4.78 (3.29, 6.91) | 4.06 (2.74, 5.97) |
| **Sensitivity 1: 2008/09-2012/13 births** |  |  |  |  |  |  |  |  |
| LA variance (95% CI) |  | 0.21 (0.14, 0.31) | 0.20 (0.13, 0.30) | 0.17 (0.11, 0.27) | 0.21 (0.14, 0.31) | 0.21 (0.14, 0.31) | 0.20 (0.13, 0.31) | 0.17 (0.11, 0.27) |
| PCV* |  |  | 4.86% | 17.13% | 0.24% | 0.30% | 2.70% | 16.47% |
| AUC (95% CI) | 0.77 (0.75, 0.78) | 0.79 (0.78, 0.81) | 0.79 (0.78, 0.81) | 0.79 (0.78, 0.81) | 0.79 (0.78, 0.81) | 0.79 (0.78, 0.81) | 0.79 (0.78, 0.81) | 0.79 (0.78, 0.81) |
| AUC change* |  | 0.022 | 0.000 | -0.001 | 0.000 | 0.000 | 0.000 | -0.002 |
| ICC % (95% CI) |  | 5.96 (4.05, 8.69) | 5.68 (3.82, 8.38) | 4.99 (3.27, 7.54) | 5.94 (4.04, 8.67) | 5.94 (4.03, 8.67) | 5.81 (3.90, 8.55) | 5.02 (3.33, 7.52) |
| **Sensitivity 2: Minimal adjustment set** |  |  |  |  |  |  |  |  |
| LA variance (95% CI) |  | 0.10 (0.07, 0.15) | 0.10 (0.07, 0.15) | 0.09 (0.06, 0.14) | 0.10 (0.07, 0.15) | 0.10 (0.07, 0.15) | 0.10 (0.06, 0.15) | 0.08 (0.05, 0.13) |
| PCV* |  |  | 1.78% | 13.59% | 0.59% | 0.33% | 2.53% | 20.00% |
| AUC (95% CI) | 0.60 (0.58, 0.61) | 0.63 (0.62, 0.65) | 0.63 (0.62, 0.64) | 0.64 (0.63, 0.65) | 0.63 (0.62, 0.65) | 0.63 (0.62, 0.65) | 0.64 (0.63, 0.65) | 0.63 (0.62, 0.64) |
| AUC change* |  | 0.040 | 0.000 | 0.001 | 0.000 | 0.000 | 0.001 | -0.001 |
| ICC % (95% CI) |  | 2.98 (1.99, 4.43) | 2.93 (1.95, 4.38) | 2.58 (1.68, 3.96) | 2.96 (1.98, 4.41) | 2.97 (1.98, 4.42) | 2.91 (1.92, 4.38) | 2.40 (1.55, 3.69) |
| **Sensitivity 3: Different random sample** |  |  |  |  |  |  |  |  |
| LA variance (95% CI) |  | 0.20 (0.14, 0.28) | 0.19 (0.13, 0.27) | 0.17 (0.12, 0.25) | 0.20 (0.14, 0.28) | 0.20 (0.14, 0.28) | 0.19 (0.13, 0.28) | 0.18 (0.13, 0.27) |
| PCV* |  |  | 3.44% | 12.03% | 0.01% | 0.05% | 2.50% | 6.12% |
| AUC (95% CI) | 0.78 (0.77, 0.79) | 0.81 (0.80, 0.81) | 0.81 (0.80, 0.81) | 0.80 (0.80, 0.81) | 0.81 (0.80, 0.81) | 0.81 (0.80, 0.81) | 0.81 (0.80, 0.81) | 0.81 (0.80, 0.81) |
| AUC change* |  | 0.02 | 0.00 | 0.00 | 0.00 | 0.00 | 0.00 | 0.00 |
| ICC % (95% CI) |  | 5.61 (3.98, 7.85) | 5.42 (3.83, 7.64) | 4.97 (3.45, 7.10) | 5.61 (3.98, 7.85) | 5.60 (3.98, 7.85) | 5.47 (3.86, 7.72) | 5.28 (3.71, 7.47) |
| *Full-term: EHCP vs. SEND support* |  |  |  |  |  |  |  |  |
| **Main analysis** |  |  |  |  |  |  |  |  |
| LA variance (95% CI) |  | 0.15 (0.09, 0.23) | 0.14 (0.09, 0.22) | 0.14 (0.09, 0.22) | 0.15 (0.09, 0.23) | 0.15 (0.09, 0.23) | 0.12 (0.07, 0.19) | 0.12 (0.07, 0.19) |
| PCV* |  |  | 3.25% | 4.11% | -0.08% | -0.11% | 17.60% | 19.06% |
| AUC (95% CI) | 0.75 (0.74, 0.76) | 0.78 (0.77, 0.79) | 0.78 (0.77, 0.79) | 0.78 (0.77, 0.79) | 0.78 (0.77, 0.79) | 0.78 (0.77, 0.79) | 0.78 (0.76, 0.79) | 0.78 (0.76, 0.79) |
| AUC change* |  | 0.02 | 0.00 | 0.00 | 0.00 | 0.00 | 0.00 | 0.00 |
| ICC % (95% CI) |  | 4.22 (2.76, 6.41) | 4.09 (2.66, 6.25) | 4.06 (2.62, 6.25) | 4.23 (2.77, 6.41) | 4.23 (2.77, 6.41) | 3.51 (2.21, 5.53) | 3.45 (2.15, 5.48) |
| **Sensitivity 1: 2008/09-2012/13 births** |  |  |  |  |  |  |  |  |
| LA variance (95% CI) |  | 0.21 (0.14, 0.31) | 0.20 (0.13, 0.30) | 0.17 (0.11, 0.27) | 0.21 (0.14, 0.31) | 0.21 (0.14, 0.31) | 0.20 (0.13, 0.31) | 0.17 (0.11, 0.27) |
| PCV* |  |  | 4.86% | 17.13% | 0.24% | 0.30% | 2.70% | 16.47% |
| AUC (95% CI) | 0.77 (0.75, 0.78) | 0.79 (0.78, 0.81) | 0.79 (0.78, 0.81) | 0.79 (0.78, 0.81) | 0.79 (0.78, 0.81) | 0.79 (0.78, 0.81) | 0.79 (0.78, 0.81) | 0.79 (0.78, 0.81) |
| AUC change* |  | 0.022 | 0.000 | -0.001 | 0.000 | 0.000 | 0.000 | -0.002 |
| ICC % (95% CI) |  | 5.96 (4.05, 8.69) | 5.68 (3.82, 8.38) | 4.99 (3.27, 7.54) | 5.94 (4.04, 8.67) | 5.94 (4.03, 8.67) | 5.81 (3.90, 8.55) | 5.02 (3.33, 7.52) |
| **Sensitivity 2: Minimal adjustment set** |  |  |  |  |  |  |  |  |
| LA variance (95% CI) |  | 0.11 (0.07, 0.17) | 0.11 (0.07, 0.17) | 0.11 (0.07, 0.17) | 0.11 (0.07, 0.17) | 0.11 (0.07, 0.17) | 0.09 (0.05, 0.14) | 0.09 (0.05, 0.14) |
| PCV* |  |  | 2.45% | 3.42% | 0.00% | 0.00% | 19.82% | 21.89% |
| AUC (95% CI) | 0.59 (0.58, 0.60) | 0.64 (0.63, 0.65) | 0.64 (0.63, 0.65) | 0.64 (0.63, 0.65) | 0.64 (0.63, 0.65) | 0.64 (0.63, 0.65) | 0.64 (0.63, 0.65) | 0.64 (0.63, 0.65) |
| AUC change* |  | 0.049 | 0.000 | 0.000 | 0.000 | 0.000 | -0.001 | -0.002 |
| ICC % (95% CI) |  | 3.23 (2.08, 4.98) | 3.15 (2.01, 4.90) | 3.12 (1.98, 4.88) | 3.23 (2.08, 4.98) | 3.23 (2.08, 4.98) | 2.60 (1.60, 4.20) | 2.54 (1.54, 4.16) |
| **Sensitivity 3: Different random sample** |  |  |  |  |  |  |  |  |
| LA variance (95% CI) |  | 0.16 (0.11, 0.24) | 0.15 (0.10, 0.23) | 0.15 (0.10, 0.23) | 0.16 (0.11, 0.24) | 0.16 (0.11, 0.24) | 0.15 (0.10, 0.23) | 0.14 (0.09, 0.22) |
| PCV* |  |  | 5.09% | 6.60% | -0.26% | -0.21% | 6.66% | 13.72% |
| AUC (95% CI) | 0.76 (0.75, 0.77) | 0.79 (0.77, 0.80) | 0.79 (0.77, 0.80) | 0.79 (0.78, 0.80) | 0.79 (0.77, 0.80) | 0.79 (0.77, 0.80) | 0.79 (0.77, 0.80) | 0.79 (0.77, 0.80) |
| AUC change* |  | 0.02 | 0.00 | 0.00 | 0.00 | 0.00 | 0.00 | 0.00 |
| ICC % (95% CI) |  | 4.68 (3.16, 6.88) | 4.45 (2.97, 6.63) | 4.38 (2.91, 6.56) | 4.69 (3.16, 6.90) | 4.69 (3.16, 6.90) | 4.38 (2.91, 6.55) | 4.06 (2.67, 6.14) |

AUC = area under the receiving operator characteristic curve; CI = confidence interval; EHCP = education, health and care plan; ICC = intraclass correlation coefficients; LA = local authority; SEND = special educational needs and disability; PCV=percentage change in variance; SEND=special educational needs and disability; *change in relation to the previous step (i.e. step 1 for step 2 model, step 2 model for step 3 models); Positive values for AUC change and PCV indicate increases in the discrimination and variance explained compared with model from the previous step

**Table S9.** LA-general effects: EHCP vs. no SEND provision – main and sensitivity analyses combined

|  | **Step one (multilevel)** | **Step two (multilevel)** | **Step three (multilevel, + pupil headcount)** | **Step three (multilevel, + special school attendance %)** | **Step three (multilevel, + maintained school attendance %)** | **Step three (multilevel, + academy school attendance %)** | **Step three (multilevel, + FSM eligible %)** | **Step three (multilevel, + IDACI mode)** |
| --- | --- | --- | --- | --- | --- | --- | --- | --- |
| *Late preterm: EHCP vs. no SEND provision* |  |  |  |  |  |  |  |  |
| **Main analysis** |  |  |  |  |  |  |  |  |
| LA variance (95% CI) |  | 0.14 (0.10, 0.20) | 0.14 (0.10, 0.20) | 0.11 (0.08, 0.17) | 0.14 (0.10, 0.20) | 0.14 (0.10, 0.20) | 0.12 (0.08, 0.18) | 0.11 (0.08, 0.17) |
| PCV* |  |  | 1.35% | 18.60% | 0.20% | 0.14% | 14.15% | 20.20% |
| AUC (95% CI) | 0.87 (0.86, 0.87) | 0.88 (0.87, 0.89) | 0.88 (0.87, 0.89) | 0.88 (0.87, 0.89) | 0.88 (0.87, 0.89) | 0.88 (0.87, 0.89) | 0.88 (0.87, 0.89) | 0.88 (0.87, 0.89) |
| AUC change* |  | 0.01 | 0.00 | 0.00 | 0.00 | 0.00 | 0.00 | 0.00 |
| ICC % (95% CI) |  | 4.07 (2.86, 5.77) | 4.02 (2.81, 5.71) | 3.34 (2.26, 4.91) | 4.06 (2.85, 5.76) | 4.07 (2.85, 5.76) | 3.52 (2.40, 5.12) | 3.28 (2.23, 4.78) |
| **Sensitivity 1: 2008/09-2012/13 births** |  |  |  |  |  |  |  |  |
| LA variance (95% CI) |  | 0.22 (0.15, 0.32) | 0.21 (0.14, 0.30) | 0.19 (0.13, 0.28) | 0.22 (0.15, 0.32) | 0.22 (0.15, 0.32) | 0.20 (0.14, 0.30) | 0.19 (0.13, 0.28) |
| PCV* |  |  | 6.87% | 14.07% | 0.49% | 0.30% | 7.86% | 14.58% |
| AUC (95% CI) | 0.80 (0.78, 0.81) | 0.82 (0.81, 0.83) | 0.82 (0.81, 0.83) | 0.82 (0.81, 0.83) | 0.82 (0.81, 0.83) | 0.82 (0.81, 0.83) | 0.82 (0.81, 0.83) | 0.82 (0.81, 0.83) |
| AUC change* |  | 0.022 | 0.000 | -0.001 | 0.000 | 0.000 | 0.000 | -0.002 |
| ICC % (95% CI) |  | 6.31 (4.48, 8.83) | 5.90 (4.14, 8.35) | 5.47 (3.79, 7.84) | 6.28 (4.45, 8.80) | 6.30 (4.46, 8.82) | 5.85 (4.09, 8.29) | 5.44 (3.77, 7.79) |
| **Sensitivity 2: Minimal adjustment set** |  |  |  |  |  |  |  |  |
| LA variance (95% CI) |  | 0.08 (0.05, 0.11) | 0.07 (0.05, 0.11) | 0.05 (0.03, 0.07) | 0.08 (0.05, 0.11) | 0.08 (0.05, 0.11) | 0.07 (0.05, 0.11) | 0.07 (0.04, 0.10) |
| PCV* |  |  | 4.94% | 39.61% | 0.01% | -0.03% | 7.04% | 11.42% |
| AUC (95% CI) | 0.54 (0.53, 0.55) | 0.60 (0.59, 0.61) | 0.60 (0.59, 0.61) | 0.60 (0.59, 0.60) | 0.60 (0.59, 0.61) | 0.60 (0.59, 0.61) | 0.60 (0.59, 0.61) | 0.60 (0.59, 0.61) |
| AUC change* |  | 0.059 | 0.000 | -0.003 | 0.000 | 0.000 | 0.000 | 0.000 |
| ICC % (95% CI) |  | 2.25 (1.53, 3.30) | 2.14 (1.45, 3.16) | 1.37 (0.84, 2.22) | 2.25 (1.53, 3.30) | 2.25 (1.53, 3.30) | 2.09 (1.40, 3.12) | 2.00 (1.34, 2.98) |
| *Early term: EHCP vs. no SEND provision* |  |  |  |  |  |  |  |  |
| **Main analysis** |  |  |  |  |  |  |  |  |
| LA variance (95% CI) |  | 0.14 (0.09, 0.20) | 0.14 (0.09, 0.21) | 0.11 (0.07, 0.17) | 0.14 (0.09, 0.20) | 0.14 (0.09, 0.20) | 0.13 (0.09, 0.20) | 0.11 (0.07, 0.17) |
| PCV* |  |  | -0.11% | 17.90% | -0.01% | 0.01% | 2.65% | 17.51% |
| AUC (95% CI) | 0.85 (0.84, 0.85) | 0.86 (0.85, 0.87) | 0.86 (0.85, 0.87) | 0.86 (0.85, 0.87) | 0.86 (0.85, 0.87) | 0.86 (0.85, 0.87) | 0.86 (0.85, 0.87) | 0.86 (0.85, 0.87) |
| AUC change* |  | 0.02 | 0.00 | 0.00 | 0.00 | 0.00 | 0.00 | 0.00 |
| ICC % (95% CI) |  | 3.98 (2.69, 5.85) | 3.98 (2.68, 5.88) | 3.29 (2.13, 5.03) | 3.98 (2.69, 5.85) | 3.98 (2.69, 5.85) | 3.87 (2.59, 5.76) | 3.30 (2.17, 5.00) |
| **Sensitivity 1: 2008/09-2012/13 births** |  |  |  |  |  |  |  |  |
| LA variance (95% CI) |  | 0.15 (0.10, 0.24) | 0.14 (0.09, 0.23) | 0.12 (0.07, 0.20) | 0.15 (0.10, 0.24) | 0.15 (0.10, 0.24) | 0.15 (0.10, 0.24) | 0.13 (0.08, 0.21) |
| PCV* |  |  | 5.66% | 22.73% | 0.17% | 0.29% | 0.15% | 14.48% |
| AUC (95% CI) | 0.84 (0.83, 0.85) | 0.86 (0.85, 0.87) | 0.86 (0.85, 0.87) | 0.86 (0.85, 0.87) | 0.86 (0.85, 0.87) | 0.86 (0.85, 0.87) | 0.86 (0.85, 0.87) | 0.86 (0.85, 0.87) |
| AUC change* |  | 0.011 | 0.000 | -0.001 | 0.000 | 0.000 | 0.000 | -0.001 |
| ICC % (95% CI) |  | 4.46 (2.87, 6.86) | 4.22 (2.68, 6.58) | 3.48 (2.11, 5.68) | 4.45 (2.86, 6.85) | 4.45 (2.86, 6.85) | 4.45 (2.84, 6.90) | 3.84 (2.41, 6.06) |
| **Sensitivity 2: Minimal adjustment set** |  |  |  |  |  |  |  |  |
| LA variance (95% CI) |  | 0.09 (0.06, 0.13) | 0.09 (0.06, 0.13) | 0.06 (0.04, 0.10) | 0.09 (0.06, 0.13) | 0.09 (0.06, 0.13) | 0.09 (0.06, 0.14) | 0.08 (0.05, 0.12) |
| PCV* |  |  | -0.08% | 26.83% | 0.28% | 0.10% | -3.31% | 7.80% |
| AUC (95% CI) | 0.55 (0.54, 0.56) | 0.60 (0.59, 0.61) | 0.60 (0.59, 0.61) | 0.60 (0.59, 0.61) | 0.60 (0.59, 0.61) | 0.60 (0.59, 0.61) | 0.60 (0.59, 0.61) | 0.60 (0.59, 0.61) |
| AUC change* |  | 0.057 | 0.000 | -0.001 | 0.000 | 0.000 | 0.001 | 0.000 |
| ICC % (95% CI) |  | 2.58 (1.71, 3.87) | 2.58 (1.70, 3.90) | 1.90 (1.18, 3.04) | 2.57 (1.71, 3.86) | 2.58 (1.71, 3.87) | 2.66 (1.76, 4.01) | 2.38 (1.56, 3.62) |
| **Sensitivity 3: Different random sample** |  |  |  |  |  |  |  |  |
| LA variance (95% CI) |  | 0.18 (0.12, 0.26) | 0.18 (0.12, 0.26) | 0.16 (0.10, 0.23) | 0.18 (0.12, 0.26) | 0.18 (0.12, 0.26) | 0.17 (0.12, 0.25) | 0.16 (0.11, 0.23) |
| PCV* |  |  | -1.94% | 12.06% | 0.12% | 0.31% | 4.18% | 9.56% |
| AUC (95% CI) | 0.85 (0.84, 0.86) | 0.87 (0.86, 0.87) | 0.87 (0.86, 0.87) | 0.87 (0.86, 0.87) | 0.87 (0.86, 0.87) | 0.87 (0.86, 0.87) | 0.87 (0.86, 0.87) | 0.87 (0.86, 0.87) |
| AUC change* |  | 0.01 | 0.00 | 0.00 | 0.00 | 0.00 | 0.00 | 0.00 |
| ICC % (95% CI) |  | 5.10 (3.57, 7.24) | 5.19 (3.63, 7.38) | 4.51 (3.09, 6.55) | 5.09 (3.56, 7.23) | 5.08 (3.56, 7.22) | 4.90 (3.39, 7.02) | 4.63 (3.20, 6.66) |
| *Full-term: EHCP vs. no SEND provision* |  |  |  |  |  |  |  |  |
| **Main analysis** |  |  |  |  |  |  |  |  |
| LA variance (95% CI) |  | 0.13 (0.08, 0.21) | 0.13 (0.08, 0.20) | 0.12 (0.08, 0.20) | 0.13 (0.08, 0.21) | 0.13 (0.08, 0.21) | 0.10 (0.06, 0.17) | 0.10 (0.06, 0.17) |
| PCV* |  |  | 2.16% | 3.61% | 0.07% | 0.05% | 21.89% | 23.82% |
| AUC (95% CI) | 0.82 (0.81, 0.83) | 0.84 (0.83, 0.85) | 0.84 (0.83, 0.85) | 0.84 (0.83, 0.85) | 0.84 (0.83, 0.85) | 0.84 (0.83, 0.85) | 0.84 (0.83, 0.85) | 0.84 (0.83, 0.85) |
| AUC change* |  | 0.02 | 0.00 | 0.00 | 0.00 | 0.00 | 0.00 | 0.00 |
| ICC % (95% CI) |  | 3.79 (2.42, 5.88) | 3.71 (2.36, 5.80) | 3.66 (2.28, 5.81) | 3.79 (2.42, 5.88) | 3.79 (2.42, 5.88) | 2.98 (1.80, 4.91) | 2.91 (1.75, 4.82) |
| **Sensitivity 1: 2008/09-2012/13 births** |  |  |  |  |  |  |  |  |
| LA variance (95% CI) |  | 0.15 (0.10, 0.24) | 0.14 (0.09, 0.23) | 0.12 (0.07, 0.20) | 0.15 (0.10, 0.24) | 0.15 (0.10, 0.24) | 0.15 (0.10, 0.24) | 0.13 (0.08, 0.21) |
| PCV* |  |  | 5.66% | 22.73% | 0.17% | 0.29% | 0.15% | 14.48% |
| AUC (95% CI) | 0.84 (0.83, 0.85) | 0.86 (0.85, 0.87) | 0.86 (0.85, 0.87) | 0.86 (0.85, 0.87) | 0.86 (0.85, 0.87) | 0.86 (0.85, 0.87) | 0.86 (0.85, 0.87) | 0.86 (0.85, 0.87) |
| AUC change* |  | 0.011 | 0.000 | -0.001 | 0.000 | 0.000 | 0.000 | -0.001 |
| ICC % (95% CI) |  | 4.46 (2.87, 6.86) | 4.22 (2.68, 6.58) | 3.48 (2.11, 5.68) | 4.45 (2.86, 6.85) | 4.45 (2.86, 6.85) | 4.45 (2.84, 6.90) | 3.84 (2.41, 6.06) |
| **Sensitivity 2: Minimal adjustment set** |  |  |  |  |  |  |  |  |
| LA variance (95% CI) |  | 0.09 (0.06, 0.15) | 0.09 (0.06, 0.14) | 0.08 (0.05, 0.13) | 0.09 (0.06, 0.15) | 0.09 (0.06, 0.15) | 0.09 (0.05, 0.14) | 0.09 (0.05, 0.14) |
| PCV* |  |  | 5.29% | 17.59% | 0.79% | 1.19% | 7.87% | 7.95% |
| AUC (95% CI) | 0.54 (0.53, 0.56) | 0.61 (0.60, 0.62) | 0.61 (0.60, 0.62) | 0.61 (0.60, 0.62) | 0.61 (0.60, 0.62) | 0.61 (0.60, 0.62) | 0.61 (0.60, 0.62) | 0.61 (0.60, 0.62) |
| AUC change* |  | 0.068 | 0.000 | 0.000 | 0.000 | 0.000 | -0.001 | -0.002 |
| ICC % (95% CI) |  | 2.80 (1.80, 4.34) | 2.66 (1.68, 4.17) | 2.32 (1.41, 3.79) | 2.78 (1.78, 4.31) | 2.77 (1.77, 4.29) | 2.59 (1.63, 4.08) | 2.58 (1.62, 4.10) |
| **Sensitivity 3: Different random sample** |  |  |  |  |  |  |  |  |
| LA variance (95% CI) |  | 0.10 (0.06, 0.16) | 0.10 (0.06, 0.16) | 0.09 (0.05, 0.15) | 0.10 (0.06, 0.16) | 0.10 (0.06, 0.16) | 0.09 (0.05, 0.15) | 0.08 (0.04, 0.14) |
| PCV* |  |  | 2.53% | 10.57% | -0.09% | -0.02% | 12.86% | 22.39% |
| AUC (95% CI) | 0.83 (0.82, 0.84) | 0.84 (0.83, 0.85) | 0.84 (0.83, 0.85) | 0.84 (0.83, 0.85) | 0.84 (0.83, 0.85) | 0.84 (0.83, 0.85) | 0.84 (0.83, 0.85) | 0.84 (0.83, 0.85) |
| AUC change* |  | 0.01 | 0.00 | 0.00 | 0.00 | 0.00 | 0.00 | 0.00 |
| ICC % (95% CI) |  | 2.95 (1.83, 4.71) | 2.88 (1.76, 4.65) | 2.65 (1.58, 4.39) | 2.95 (1.83, 4.72) | 2.95 (1.83, 4.71) | 2.58 (1.54, 4.30) | 2.30 (1.34, 3.94) |

AUC = area under the receiving operator characteristic curve; CI = confidence interval; EHCP = education, health and care plan; ICC = intraclass correlation coefficients; LA = local authority; SEND = special educational needs and disability; PCV=percentage change in variance; SEND=special educational needs and disability; *change in relation to the previous step (i.e. step 1 for step 2 model, step 2 model for step 3 models); Positive values for AUC change and PCV indicate increases in the discrimination and variance explained compared with model from the previous step
